# RecQ helicases in the malaria parasite *Plasmodium falciparum* affect genome stability, gene expression patterns and DNA replication dynamics

**DOI:** 10.1371/journal.pgen.1007490

**Published:** 2018-07-02

**Authors:** Antoine Claessens, Lynne M. Harris, Slavica Stanojcic, Lia Chappell, Adam Stanton, Nada Kuk, Pamela Veneziano-Broccia, Yvon Sterkers, Julian C. Rayner, Catherine J. Merrick

**Affiliations:** 1 London School of Hygiene and Tropical Medicine, London, United Kingdom; 2 Medical Research Council Unit The Gambia, Fajara, Banjul, The Gambia; 3 Centre for Applied Entomology and Parasitology, Faculty of Natural Sciences, Keele University, Keele, Staffordshire, United Kingdom; 4 University of Montpellier, Faculty of Medicine, Laboratory of Parasitology-Mycology, Montpellier, France; 5 Malaria Programme, Wellcome Sanger Institute, Hinxton, Cambridge, United Kingdom; 6 School of Computing and Mathematics, Faculty of Natural Sciences, Keele University, Keele, Staffordshire, United Kingdom; 7 CNRS 5290 - IRD 224 - University of Montpellier (UMR “MiVEGEC”), Montpellier, France; 8 University Hospital Centre (CHU), Department of Parasitology-Mycology, Montpellier, France; 9 Department of Pathology, Cambridge University, Cambridge, United Kingdom; University of Virginia, UNITED STATES

## Abstract

The malaria parasite *Plasmodium falciparum* has evolved an unusual genome structure. The majority of the genome is relatively stable, with mutation rates similar to most eukaryotic species. However, some regions are very unstable with high recombination rates, driving the generation of new immune evasion-associated *var* genes. The molecular factors controlling the inconsistent stability of this genome are not known. Here we studied the roles of the two putative RecQ helicases in *P*. *falciparum*, *Pf*BLM and *Pf*WRN. When *Pf*WRN was knocked down, recombination rates increased four-fold, generating chromosomal abnormalities, a high rate of chimeric *var* genes and many microindels, particularly in known ‘fragile sites’. This is the first identification of a gene involved in suppressing recombination and maintaining genome stability in *Plasmodium*. By contrast, no change in mutation rate appeared when the second RecQ helicase, *Pf*BLM, was mutated. At the transcriptional level, however, both helicases evidently modulate the transcription of large cohorts of genes, with several hundred genes—including a large proportion of *var*s—showing deregulated expression in each RecQ mutant. Aberrant processing of stalled replication forks is a possible mechanism underlying elevated mutation rates and this was assessed by measuring DNA replication dynamics in the RecQ mutant lines. Replication forks moved slowly and stalled at elevated rates in both mutants, confirming that RecQ helicases are required for efficient DNA replication. Overall, this work identifies the *Plasmodium* RecQ helicases as major players in DNA replication, antigenic diversification and genome stability in the most lethal human malaria parasite, with important implications for genome evolution in this pathogen.

## Author summary

Human malaria is caused by *Plasmodium* parasites, with most of the mortality (almost half a million deaths each year) being caused by one species, *Plasmodium falciparum*. This parasite has an unusual genome: it is exceptionally biased towards A and T nucleotides rather than G and C, and it contains specific areas rich in hypervariable virulence-associated genes which evolve very rapidly to produce new variants. This evolution is probably vital for the parasite to evade the human immune system and maintain chronic infections, but how it is controlled at a molecular level remains unknown. We have identified a helicase in the parasite with a huge influence on genome stability and the rate of genome evolution. It appears to function by unwinding various unusual DNA structures, and if this fails then the genome becomes unstable. In addition, the transcription of many genes whose DNA tends to form secondary structures is affected, and DNA replication is impeded. If this helicase was expressed variably in different parasite strains infecting humans, it could influence the parasites’ ability to grow and replicate efficiently, and also, crucially, its ability to evolve and thus evade the human immune system.

## Introduction

Protozoan *Plasmodium* parasites are the causative agents of human malaria, a disease responsible for widespread morbidity and almost half a million deaths each year [1]. Most malaria deaths are caused by the species *P*. *falciparum*, although four other *Plasmodium* species also infect humans.

*P*. *falciparum* has one of the most highly A/T-biased genomes ever sequenced, at ~81% A/T [2]. This is maintained by a high mutational bias towards G/C to A/T transitions [3] and the resultant genome contains a preponderance of A/T repeat tracts [4]. These are prone to form structures such as hairpins and slipped-strand pairing, and they can expand and contract readily, producing a genome that is very prone to micro-indels [3]. Simple A/T repeats can also promote the duplication of whole genes via homologous recombination (HR) between repeats—a mechanism that favours genome evolution via gene duplication followed by functional diversification and/or selection [5, 6]. This is specifically implicated in the evolution of drug resistance (e.g. amplification of the multi-drug resistance gene *PfMDR1*). As expected given the A/T bias, guanine-rich motifs are contrastingly very scarce. Only ~80 putative G-quadruplex (G4) forming sequences (non-double-helical structures that require four closely-spaced tracts of at least three guanines to form [7]) are found outside the intrinsically guanine-rich telomeres in *P*. *falciparum* [8, 9]. Both hairpins and G4s in DNA are implicated in stalling RNA and DNA polymerases, and in promoting recombination events via DNA breakage at stalled replication forks [10, 11].

*Plasmodium* species repair DNA breaks primarily by homologous recombination because they lack a conventional non-homologous end joining pathway [12]. The *Plasmodium* genome is, however, haploid (except for a brief diploid stage during sexual reproduction in the mosquito vector), so templates for HR are limited. The natural occurrence of HR during mitotic growth of haploid *P*. *falciparum* parasites in human erythrocytes has been quantified, showing that it occurs almost exclusively in subtelomeric regions and in chromosome-internal hypervariable regions. Both these regions contain genes from multi-gene families that encode variant surface antigens, such as *var* genes [13, 14]. *Var* genes encode a virulence factor called *P*. *falciparum* Erythrocyte Membrane Protein 1 (PfEMP1) that is expressed on the surface of parasite-infected erythrocytes [15–17], and *P*. *falciparum* can maintain chronic infections via antigenic switching of this factor.

*Var* recombination events usually generate new, functional chimeric genes [13, 14]: in terms of genome evolution they are thus distinct from the gene duplications that can be selected under drug pressure. *Var* recombination appears to be selectively neutral in *in vitro* culture, but *in vivo* it could diversify the repertoire of antigens and enhance the parasite’s capacity for immune evasion. The *var* recombination breakpoints that occur during *in vitro* culture are spatially associated with potential helix-disrupting structures including hairpins [18] and G4s [9], suggesting that HR is initiated when replication forks stall at such structures. In fact, the observation that sequences with the potential to form G4s are strongly clustered around *var* genes [9] suggests that such rare sequences may actually be maintained for this purpose, conferring an evolutionary advantage in *var* gene diversification.

In model systems, non-canonical DNA structures including G4s are targeted by ‘RecQ’ helicases, which suppress the stalling of replication forks and hence recombination events [19], as well as modulating transcription through non-canonical DNA structures [20]. We therefore investigated whether either recombination or transcription in the *P*. *falciparum* genome could be affected by the two putative RecQ helicases encoded by this parasite, *Pf*WRN and *Pf*BLM [8, 9, 21, 22]. Characterisation of RecQ mutant lines revealed that these helicases have widespread and profound effects on genome recombination and diversification of the virulence gene repertoire, as well as on transcription of large cohorts of genes. We also measured DNA replication dynamics at a single-molecule level via DNA combing [23], revealing that the RecQ helicases affect the speed at which replication forks move and the rate at which they stall, and thus providing a mechanistic explanation for the genome stability and transcriptional phenotypes. Overall, this work identifies the *P*. *falciparum* RecQ helicases as major players in DNA replication, antigenic diversification and genome stability in the most important human malaria parasite.

## Results

### The two RecQ helicases in *P*. *falciparum* influence parasite growth

**Fig 1:**
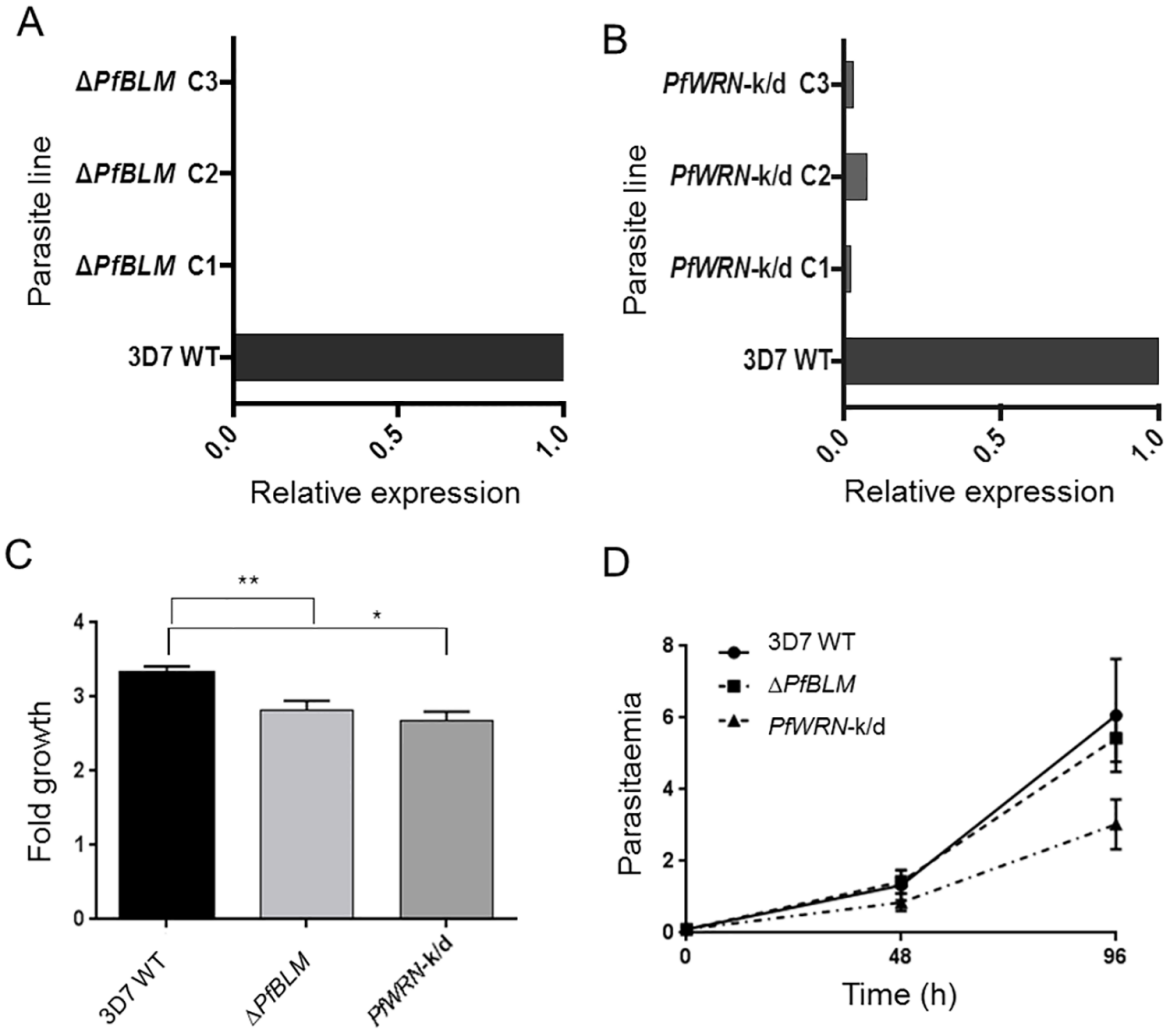
Loss of RecQ helicases affects parasite growth. (A) *PfBLM* expression in a panel of Δ*PfBLM* clones compared to wildtype 3D7 parasites. *PfBLM* transcript levels were determined by real-time PCR analysis using primer pair *PfBLM_F/R*. In each clone, relative expression of *PfBLM* was calculated by comparison to the control gene seryl-tRNA-synthetase. In the wildtype 3D7 line this relative expression level was set to 1 and *PfBLM* expression in each clone was then expressed as a fraction of this level. PCR was carried out in technical duplicate and the complete lack of *PfBLM* expression in Δ*PfBLM* clones was confirmed by agarose gel electrophoresis of PCR reaction products. (B) *PfWRN* expression in a panel of *PfWRN*-k/d clones compared to wildtype 3D7 parasites. Primer pair *PfWRNjp2F/R*, which spans a region of the *PfWRN* locus that should be absent if gene replacement via double homologous recombination had occurred, was used to detect *PfWRN* expression. Seryl-tRNA-synthetase and fructose bisphosphate aldolase were used as control genes for normalisation, with *PfWRN* expression being calculated by comparison to the mean expression level of these two genes. As in (A), relative *PfWRN* expression was then set to 1 in the wildtype 3D7 control and expression in each clone was expressed as a fraction of this level. PCR was carried out in technical duplicate and the low level of *PfWRN* expression in *PfWRN*-k/d clones was confirmed by agarose gel electrophoresis. (C) Parasite growth in the Δ*PfBLM*, *PfWRN*-k/d and parent lines, assessed by a standard 48h growth assay. Mean of three biological replicates, each conducted in technical triplicate, is shown; error bars are standard error of the mean and statistical significance was determined using one-tailed t-tests (*, p<0.05; **, p<0.01). (D) Parasite growth counted at 48h intervals over two growth cycles in the Δ*PfBLM*, *PfWRN*-k/d and parent lines, after seeding parasites at 0.1% parasitaemia. Mean growth in three biological replicates, each conducted in technical triplicate, is shown; error bars are standard error of the mean.

To investigate the roles of RecQ helicases in *P*. *falciparum*, the two genes that encode *Pf*BLM and *Pf*WRN were targeted for gene replacement via double homologous recombination. *PfBLM* was successfully knocked out, as shown by Southern blotting of the disrupted locus (S1A Fig). The absence of a full-length transcript in the mutant line was confirmed by RT-PCR (Fig 1A) and RNA-Seq (S1B Fig). In the case of *PfWRN*, gene knockout did not occur as predicted; the endogenous locus was disrupted (S2A Fig) but Southern blotting suggested that a two-plasmid concatamer had integrated into this locus via a single recombination event at the 5’ gene-targeting sequence: this was confirmed by whole-genome sequencing (S2B Fig) and RNA-Seq (S2C Fig). The integration produced a promoter-less copy of the gene, truncated at the 5’ end, but the rest of the *PfWRN* transcript was still expressed at a low level (<7% of wildtype, as detected by RT-PCR, Fig 1B), and this truncated transcript could potentially produce a protein with residual helicase function. This line, termed ‘*PfWRN-*k/d’, therefore represents a >90% knockdown, at least at the RNA level, rather than a genuine knockout of *Pf*WRN. Notably, several rounds of unsuccessful negative selection were conducted before this parasite line was obtained, perhaps because complete loss of the *Pf*WRN helicase is lethal for blood-stage *P*. *falciparum* parasites. In keeping with this hypothesis, survival of the *PfWRN*-disruptant that was eventually obtained would require the thymidine kinase negative selection marker to be largely silenced (as supported by RNA-Seq data in S2C Fig). Overall, this complex genetic event implies a strong selection pressure against complete functional inactivation of *Pf*WRN.

Both of the RecQ mutant lines showed slight growth defects and in the case of *PfWRN-*k/d the defect was significant in two independent assays (Fig 1C and 1D). Parasites in both lines appeared morphologically normal and had normal cell cycle dynamics (S3A Fig); the growth defects may be partially attributable to slight reductions in the numbers of merozoites formed per schizont (S3B and S3C Fig). These phenotypes corroborate results from the PlasmoGEM project, in which growth phenotypes were generated for genome-wide gene knockouts in the rodent malaria model species *P*. *berghei* [24]. Genetic modification in *P*. *berghei* is more efficient than in *P*. *falciparum*, allowing the creation of mutants in moderately deleterious genes, and mutants in both RecQ homologues were indeed successfully obtained in this screen (S3D Fig). Deletion of the *BLM* homologue in *P*. *berghei* affected parasite growth only slightly (average growth in 3 mice was 87% relative to wild-type: too marginal to be statistically significant with a 95% confidence interval of 0.7–1.05), whereas deletion of the *WRN* homologue led to a significantly slow growth phenotype at an average of 71% relative to wild-type (95% C.I. 0.56–0.86).

### Disruption of the *Pf*WRN helicase increases the indel mutation rate, generating extra chimeric var genes but affecting genome stability

**Fig 2:**
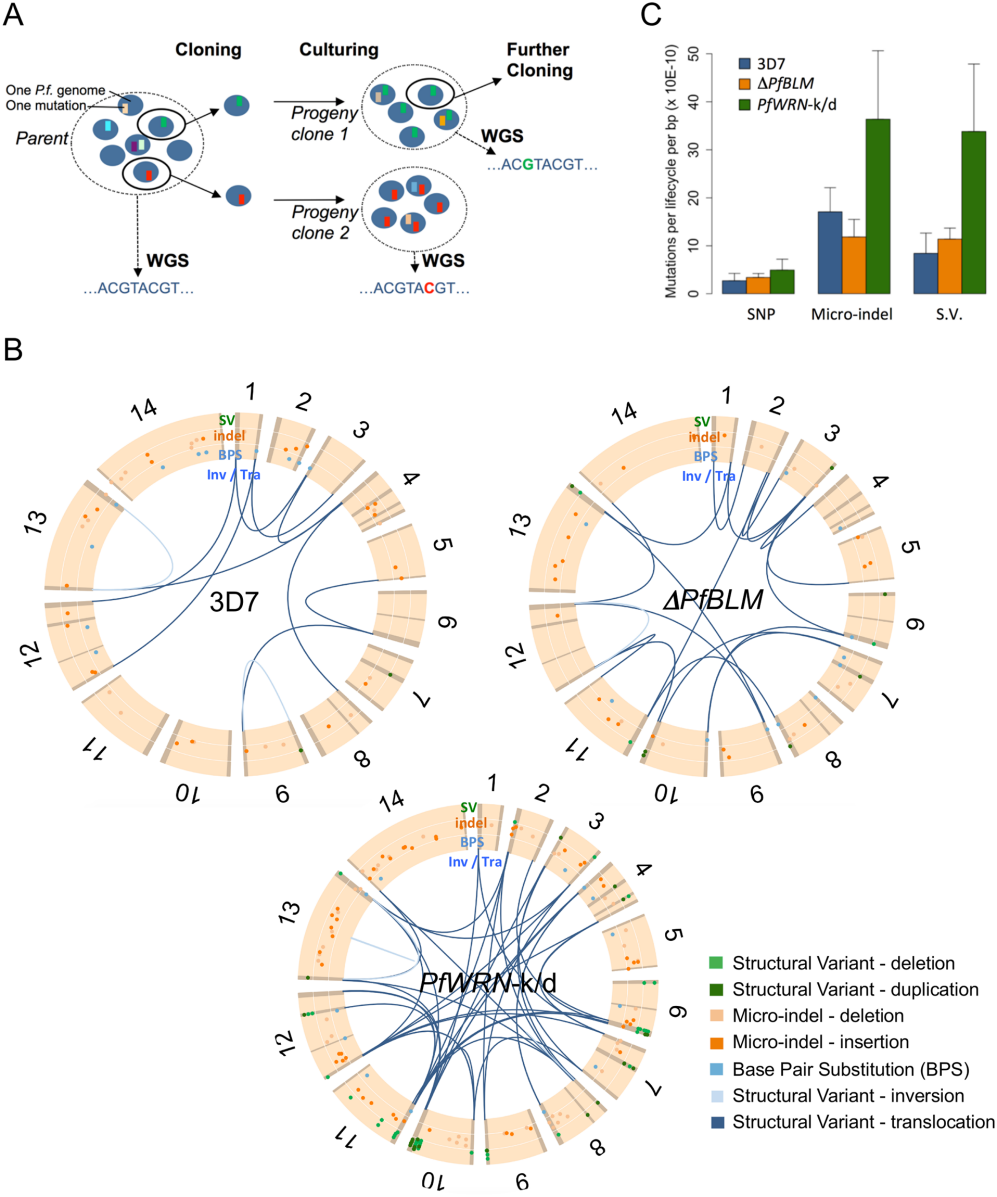
Clone trees reveal a large increase in micro-indels and SV mutations in *PfWRN*-k/d line. (A) A clone tree involves regularly cloning out individual parasites, then growing up and sequencing the bulked-up clones, in order to identify potential *de novo* mutations. Here each blue disk represents a single parasite genome, a coloured square is a *de novo* mutation. Cloning by limiting dilution randomly selects one individual parasite, possibly with one or more mutations. Bulking up the culture and sequencing will reveal the new mutation. Note that mutations observed in a progeny sample had occurred within the parental generation, at some point before the clonal dilution. (B) Chromosomal locations of all *de novo* mutations: BPS (or SNP), micro-indels and Structural Variants (SV). The 14 *P*. *falciparum* chromosomes are represented with hypervariable regions (subtelomeric and internal) in darker shades. SNPs and micro-indels are scattered throughout the genome, while SVs are found in hypervariable regions. (C) Mutation rate in 3D7, Δ*PfBLM* and *PfWRN*-k/d lines. The micro-indel and SV rates increased by 2.3 and 4.2 fold in *PfWRN*-k/d lines compared to their wild-type counterpart, respectively.

To investigate whether disrupting the RecQ helicases could affect the rate or pattern of genomic mutations, ‘clone trees’ were constructed in both the mutant lines as well as in their wildtype parent. A clone tree can track the accumulation of mutations over long periods of *in vitro* growth, via repeated cloning and whole genome sequencing of successive generations of clones [13] (Fig 2A). The total numbers of clones sequenced and their cumulative days in culture were: 27 clones and 1324 days for the parent 3D7 line; 36 clones and 1860 days for Δ*PfBLM;* 20 clones and 2183 days for *PfWRN-*k/d (S4 Fig). Libraries were made without a PCR step to avoid any bias, yet the sequencing was deep enough to cover an average of 95% of the 3D7 reference genome with 10 or more reads (S1 Spreadsheet, Sheet1).

We investigated the following events in all three clone trees: base pair substitutions (BPS, the mutation origin of single nucleotide polymorphisms, SNP), micro-indels (insertions or deletions, typically shorter than 15bp, found in microsatellite regions) and structural variants (deletions, duplications, inversions, all greater than 300bp, and translocations, which are recombinations between non-homologous chromosomes) (Fig 2B).

BPS showed an excess of G:C to A:T substitutions (S1 Spreadsheet, Sheet2), as previously demonstrated [3]. The mean substitution rate was increased by 1.8 fold in *PfWRN-*k/d compared to 3D7 (*P* = 0.040 by weighted t-test); there was no difference in the Δ*PfBLM* line.

As shown in previous studies, micro-indels were consistently detected within repetitive regions across the entire genome (S1 Spreadsheet, Sheet3; S5 Fig). There was a significant 2.3 fold increase of micro-indels in the *PfWRN*-k/d line compared to 3D7 (P < 0.007 by weighted t-test), while micro-indels were slightly decreased in the Δ*PfBLM* line. There was no difference in the chromosomal location or lengths of the micro-indels identified across all 3 lines (S6 Fig). However, the proportion of micro-indels occurring in homorepeats ([A]^n^ or [T]^n^) tripled in *PfWRN*-k/d (P < 0.0006, chi-square test) (S6G Fig). Therefore, the disruption of the *WRN* gene led to poor replication reliability in microsatellite regions, suggesting that *Pf*WRN has a role in preventing DNA polymerase slippage, particularly in homorepeat regions.

Structural Variants (SVs) were also drastically increased (4.2 fold) in the *PfWRN*-k/d line compared to 3D7 (P < 2.8E-08 by weighted t-test), with again no significant change in the Δ*PfBLM* line. The vast majority of SVs were restricted to hypervariable regions (subtelomeric or internal) which contain highly polymorphic genes such as *var* genes. As previously observed, in all three lines, the breakpoints occurred precisely within a short sequence (median 15bp) termed an ‘identity block’ with 100% identity between the two recombining *var* genes. Ectopic recombinations occurring within such genes create chimeric sequences. The recombination generally involves *var* domains of the same class and the breakpoint is located in a short region of higher homology, keeping the same gene architecture and keeping the sequence in frame [13]. However, in *PfWRN*-k/d we identified two examples of a group A *var* gene recombining with a group B gene, leading to abnormal, presumably non-functional, chimeras (S1 Spreadsheet, Sheet4; S7 Fig). This had never been observed in any *in vitro* study of recombination dynamics [3, 13], and is predicted to be very rare in the wild [25]. Other abnormalities uniquely found in the *PfWRN*-k/d clone tree included: (a) a recombination, with two cross-over events, between a subtelomeric *var* and an internal *var* gene (S8 Fig); (b) an inversion of the mirror-image sequence of the invasion-related genes *RH2a* and *RH2b* (S9 Fig); (c) insertion/duplication hotspots within the liver stage antigen *LSA1* gene and the gametocyte specific *Pf11-1* gene (S10 Fig). The latter gene is extremely repetitive and is known to be in a fragile region [26], but in that gene alone we identified 8 unique mutations in 19 *PfWRN*-k/d subclones [S10B Fig], versus none in any other clone tree published so far. In conclusion, the lack of a fully functional WRN helicase led to a large increase in the structural variant mutation rate, and also a wider range of chromosomal abnormalities.

### Indels and structural variants are associated with DNA sequences that can form secondary structures

The data described in Fig 2 were further analysed by assessing the relationship between mutation events and putative DNA secondary structures, such as might be targeted by RecQ helicases. First, we calculated the average distance between mutation events and putative G-quadruplex forming sequences (PQSs—the guanine rich motifs that have the potential to form G4s). We have previously reported that recombination events in wildtype parasites are highly associated with PQSs [9], and this association held for recombination events leading to SVs in all three parasite lines analysed here (Table 1). Indeed, the median distance between a breakpoint and a PQS in wildtype 3D7 parasites was almost identical in our previous analysis and in this new dataset (16.4kb and 17.1kb). In the *PfWRN*-k/d line, however, the median PQS-to-breakpoint distance was 50% longer at 26.1kb. Unlike SVs, indels and SNPs were not spatially associated with PQSs, suggesting that G4s are not an initiating event for indel or SNP mutations.

**Table 1:** Analysis of association between mutation events and PQSs.

Mutation type	Parasite line	Number	Mean distance from PQS (kb)	Median distance from PQS (kb)	PQS association?	Different from 3D7 WT?
S.V.	3D7 WT	44	29.6	17.1, C.I. 12.3–27.5	Y	
	Δ*PfBLM*	77	68.2	13.6, C.I. 9.0–22.2	Y	N
	*PfWRN*-k/d	298	84.5	26.1, C.I. 18.9–33.3	Y	Y
indel	3D7 WT	43	292.4	181.0, C.I. 98.7–310.7	N	
	Δ*PfBLM*	32	236.0	177.7, C.I. 59.0–288.9	N	N
	*PfWRN*-k/d	96	322.1	227.7, C.I. 144.7–298.1	N	N
SNP	3D7 WT	11	280.3	89.0, C.I. 2.6–923.3	N	
	Δ*PfBLM*	10	144.5	58.2, C.I. 2.1–265.3	N	N
	*PfWRN*-k/d	13	215.9	71.6, C.I. 4.3–416.7	N	N
Null simulation		1x10^6^	301.0	180.4		

For each type of mutation in each parasite line, the table shows mean and median distances from the nearest PQS (calculated as in Stanton *et al*. 2016 [9]). Mutations are significantly associated with PQSs if the median falls outside the 95% confidence interval (C.I.) for the null dataset, which comprises distances between PQSs and random simulated breakpoints [9]. Medians rather than means are used because of the strong skew in these non-normally-distributed datasets.

Micro-indels consistently occurred in regions with significantly high tandem repeats (TR) or regions of low sequence complexity (LCR)–as expected if they originate from polymerase stalling or stuttering at mispaired DNA—and this association was the same in the parent line and the RecQ mutants (Table 2). The breakpoints of SVs tended to occur in high-TR regions, suggesting that SVs can have the same mechanistic origin as micro-indels, i.e. impeded polymerase movement through repetitive DNA. However, this relationship was weaker for SVs than for micro-indels and reached significance only in the largest dataset (SVs from *PfWRN*-k/d). SNPs, as expected, were largely unrelated to the TR/LCR environment (and since all SNP datasets were very small, any bias in SNP occurrence in wildtype versus mutant parasites may not be meaningful). Overall, micro-indels were strongly associated with repeat-rich DNA; SVs were weakly associated with repeat-rich DNA and strongly associated with PQSs, and SNPs were not associated with such DNA at all.

**Table 2:** Analysis of association between mutation events and tandem-repeat or low-complexity DNA regions.

Mutation type	Parasite line	Number	Mean TR content (%)	Different from whole genome?	Different from 3D7 WT?	Mean LCR content (%)	Different from whole genome?	Different from 3D7 WT?
S.V.	3D7 WT	44	17.9	N (p = 0.229)		19.3	Marginal (p = 0.014)	
	Δ*PfBLM*	77	19.1	N (p = 0.068)	N (p = 0.848)	13.6	Y (p < 0.001)	N (p = 0.195)
	*PfWRN*-k/d	298	17.8	Y (p < 0.001)	N (p = 0.976)	12.1	Y (p < 0.001)	N (p = 0.053)
indel	3D7 WT	43	19.3	Y (p = 0.009)		40.0	Y (p = 0.006)	
	Δ*PfBLM*	32	21.2	Y (p = 0.006)	N (p = 0.653)	37.6	Y (p < 0.001)	N (p = 0.577)
	*PfWRN*-k/d	96	15.8	Y (p < 0.001)	N (p = 0.219)	38.8	Y (p < 0.001)	N (p = 0.700)
SNP	3D7 WT	11	20.9	N (p = 0.152)		32.8	N (p = 0.517)	
	Δ*P*fBLM	10	20.4	N (p = 0.484)	N (p = 0.967)	9.6	Y (p = 0.003)	Marginal (p = 0.013)
	*PfWRN*-k/d	13	12.8	N (p = 0.819)	N (p = 0.248)	18.3	Marginal (p = 0.021)	N (p = 0.088)
Whole genome		23287	12			28.1		
ORFs only		5602	7.8			19.5		

For each type of mutation in each parasite line, the table shows the mean percentage of tandem-repeat (TR) or low-complexity (LCR) DNA in the 1kb surrounding each mutation. Significant differences between these percentages and the whole-genome average (calculated from 1kb windows across the whole genome) were assessed by 2-tailed t-test. The average TR/LCR content of all genomic ORFs was also calculated, for comparison with the whole-genome average, and as expected ORFS contained a lower percentage of TRs/LCRs.

### RecQ helicases influence G-quadruplex-related phenotypes in *P*. *falciparum*

All the recombination events that generated SVs, including those so strikingly abundant in the *PfWRN*-k/d line, were associated with PQSs, and furthermore the PQS-to-breakpoint distance was specifically extended in the *PfWRN*-k/d line (Table 1), suggesting that DNA processing at or around G4s is altered in this mutant. The link between G4s and RecQ helicases was followed up by measuring two additional G4-related phenotypes in the RecQ mutant lines: sensitivity to G4-stabilising drugs and telomere maintenance. Drug sensitivity was measured because we have previously reported than G4-stabilizing drugs can affect the growth of wildtype parasites [27], while telomeres were measured because the great majority of PQSs in the *P*. *falciparum* genome are found in the inherently G-rich telomere repeats [8].

G4s were stabilized using a matched pair of drugs, 5,10,15,20-tetra-(N-methyl-4-pyridyl)porphine (TMPyP4) [28] and its structural analogue TMPyP2, which has a markedly lower G4-binding affinity than TMPyP4 [29]. Wildtype 3D7 parasites are more sensitive to TMPyP4 than TMPyP2, suggesting that disruption of G4 metabolism, rather than any off-target drug effect, can inhibit healthy parasite growth [27]. When these two drugs were tested on the RecQ mutant lines, both lines were more sensitive than their wildtype parent: Δ*PfBLM* showed significantly increased sensitivity to both drugs, while *PfWRN*-k/d showed a trend towards increased sensitivity (S11A Fig). In both mutants the stronger G4-binding analogue, TMPyP4, remained more toxic than TMPyP2.

To assess the effect of RecQ helicases on telomere maintenance, telomere restriction fragment length Southern blotting was used. Telomeres in several Δ*PfBLM* clones were slightly but consistently longer than those of the parent line (S11B Fig), suggesting that telomere ‘set-point’ maintenance was subtly disrupted. Telomere lengths in *PfWRN-*k/d clones did not show any consistent change (S11C Fig).

### Disruption of RecQ helicases affects genome-wide transcriptional patterns

**Fig 3:**
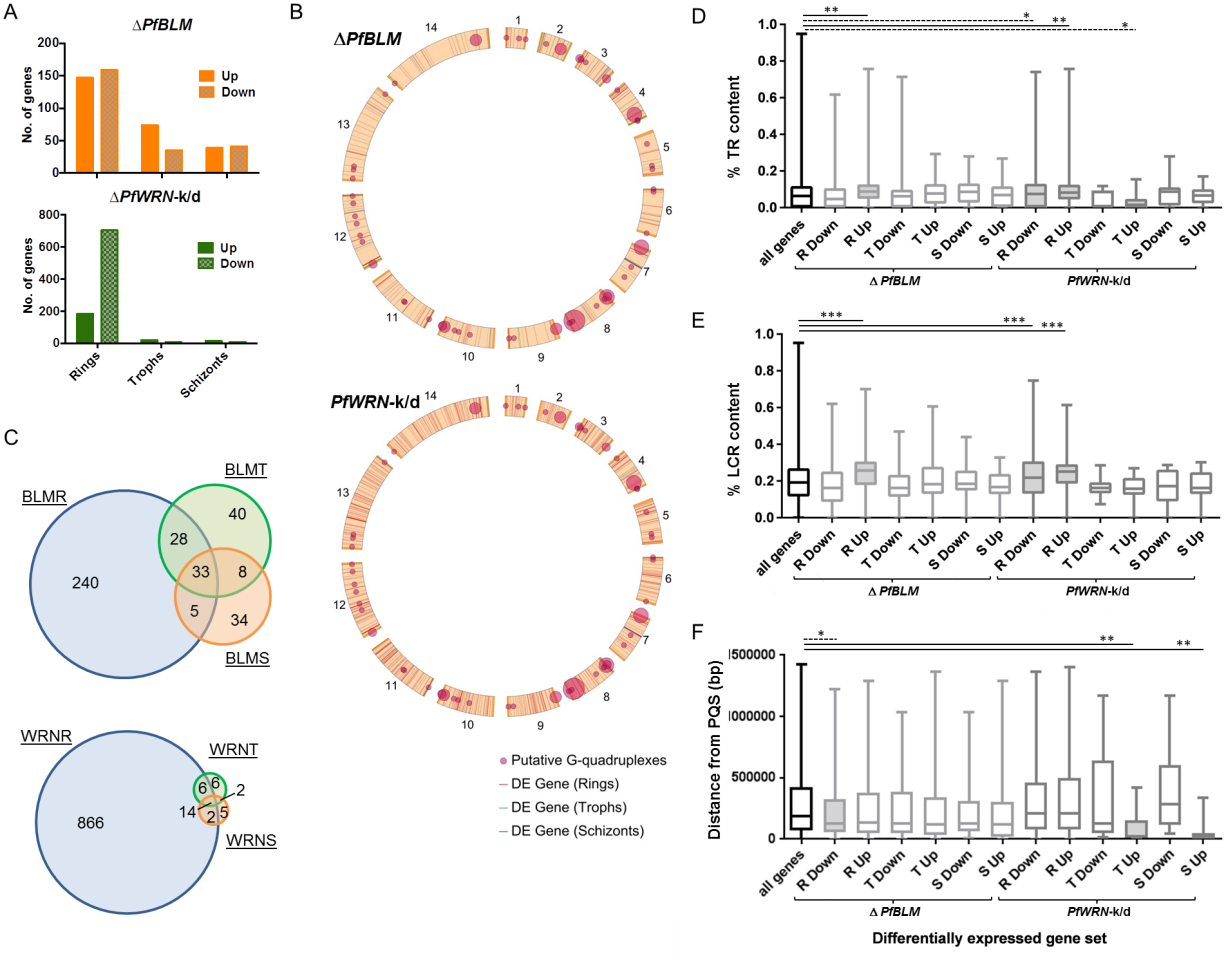
Disruption of RecQ helicases affects genome-wide transcriptional patterns. (A) Bar graphs showing the number of up-or down-regulated genes (filled and hatched bars respectively) in Δ*PfBLM* and *PfWRN-*k/d parasites at ring, trophozoite and schizont stages. (B) Plots showing the genomic locations of all differentially-expressed (DE) genes in Δ*PfBLM* and *PfWRN-*k/d parasites compared to locations of PQSs across the 14 chromosomes. Circles representing PQSs are scaled in diameter according to the number found within each ~64kb of the genome, represented by 1° of the 360° in this circular schematic. Overlapping circles occur in places where many PQSs lie within a single segment, making the circle large enough to overlap with adjacent segments. (C) Venn diagrams showing the number of genes differentially expressed in Δ*PfBLM* (upper panel) or *PfWRN-*k/d (lower panel) parasites at one or more time points (R, rings; T, trophozoites; S, schizonts). (D) Box-plot showing the percentage tandem-repeat (TR) content of differentially expressed genes in RecQ helicase mutants at rings (R), trophozoites (T) and schizonts (S). Genes are divided into Up and Down regulated subsets. Lines indicate medians, box and whiskers indicate interquartile and full ranges. Shaded boxes represent a statistically significant difference in the mean from that of the null dataset (all genes in the genome) by 2-tailed t-test: *, p<0.05; **, p<0.01; ***, p<0.001. (E) Box-plot as in (A), showing the percentage low-complexity-region (LCR) content of differentially expressed genes in RecQ helicase mutants. (F) Box-plot as in (A), showing distances between PQSs and genes differentially expressed in RecQ helicase mutants.

Moving from the genomic to the transcriptomic level, RNA-Seq was conducted in both mutant lines to investigate whether the RecQ helicases might influence the ability of parasites to transcribe through non-canonical DNA structures (S2, S3 and S4 Spreadsheets). Both mutants showed major transcriptional changes, with several hundred genes throughout the genome being differentially expressed at statistically significant levels. An additional fold-change cutoff was applied and 1434 genes showed >1.5-fold change in at least one lifecycle stage (Fig 3A), with 906 and 261 of these genes exhibiting >2-fold and >3-fold changes respectively (S3 Spreadsheet). These genes occurred on all chromosomes and were not obviously clustered at subtelomeres or near to PQSs (Fig 3B). Interestingly, deregulated expression was primarily seen in ring-stages, despite the fact that the peak of both *PfBLM* and *PfWRN* transcription in blood-stage parasites occurs in trophozoites. The sets of genes affected in the two mutant lines showed only limited overlap, either between stages (Fig 3C) or between the two mutants (S12 Fig). Many different gene ontologies were represented (S3 Spreadsheet) and there was no obvious pattern in the GO terms associated with deregulated genes.

We then assessed whether differentially-expressed genes were associated with particular genomic features that could form DNA secondary structures, using the same approach as for the recombination breakpoints. Genes deregulated in the ring stages of both mutants had markedly elevated TR and LCR content compared to all genes in the genome as a whole. This was particularly true of the gene sets *up*regulated rather than *down*regulated in rings, and did not apply to any of the gene sets identified in trophozoites or schizonts (Fig 3D and 3E, S1 Table). Elevated TR/LCR content is not an intrinsic property of genes expressed primarily at rings (S13 Fig). Therefore these data suggest that genes with a high TR/LCR content are specifically prone to deregulation in RecQ helicase mutants at the ring stage. Comparing deregulated genes with the positions of PQSs revealed a weak positive association in the Δ*PfBLM* line between PQSs and genes deregulated at all three lifecycle stages (Fig 3F). In *PfWRN-*k/d, there was no association at all between PQSs and deregulated genes in ring stages, but in trophozoites and schizonts the small numbers of upregulated genes were specifically and strikingly associated with PQSs (median gene-to-PQS distances of just 22kb in trophozoites and 11kb in schizonts).

### Disruption of RecQ helicases affects var gene transcriptional patterns

*Var* genes contain about half of all non-telomeric PQSs [8, 9] and their recombination patterns are clearly affected in the RecQ mutant lines (see Fig 2). However, RNA-Seq is not the best assay to assess changes in *var* gene transcription because these genes naturally undergo stochastic transcriptional switching and therefore the mutant lines may not have the same transcriptional ‘starting point’ as their parent. Therefore, gene-specific RT-PCR was conducted across the *var* gene family in several recent clones of each RecQ mutant line alongside similarly-aged clones of the parental line.

Changes were observed in both Δ*PfBLM* and *PfWRN-*k/d clones (S14 and S15 Figs): in Δ*PfBLM* clones, *var* genes were expressed at significantly elevated overall levels (S14A and S14B Fig) and with a fixed pattern (i.e. similar *var* genes expressed in every clone (S14A and S14B Fig) with the total variation being only 27% of that seen between clones of the parent line (S15D Fig)). In *PfWRN-*k/d clones, there was much less ‘fixing’ of the *var* genes expressed, with 74% of the wildtype level of variation (S15D Fig). In both lines, however, *var* genes having a PQS within or upstream of the gene were disproportionately affected (S14C Fig), with transcription being most strongly elevated when a PQS was on the antisense strand (S14D Fig). The deregulated expression of *var* genes in RecQ mutant lines probably relates to their PQS motifs rather than to any other RecQ-targeted motifs such as hairpins, because *var* genes do not have an unusually high TR/LCR content compared with other ring-stage-expressed genes (S13B and S13D Fig).

### RecQ helicases influence DNA replication dynamics

**Fig 4:**
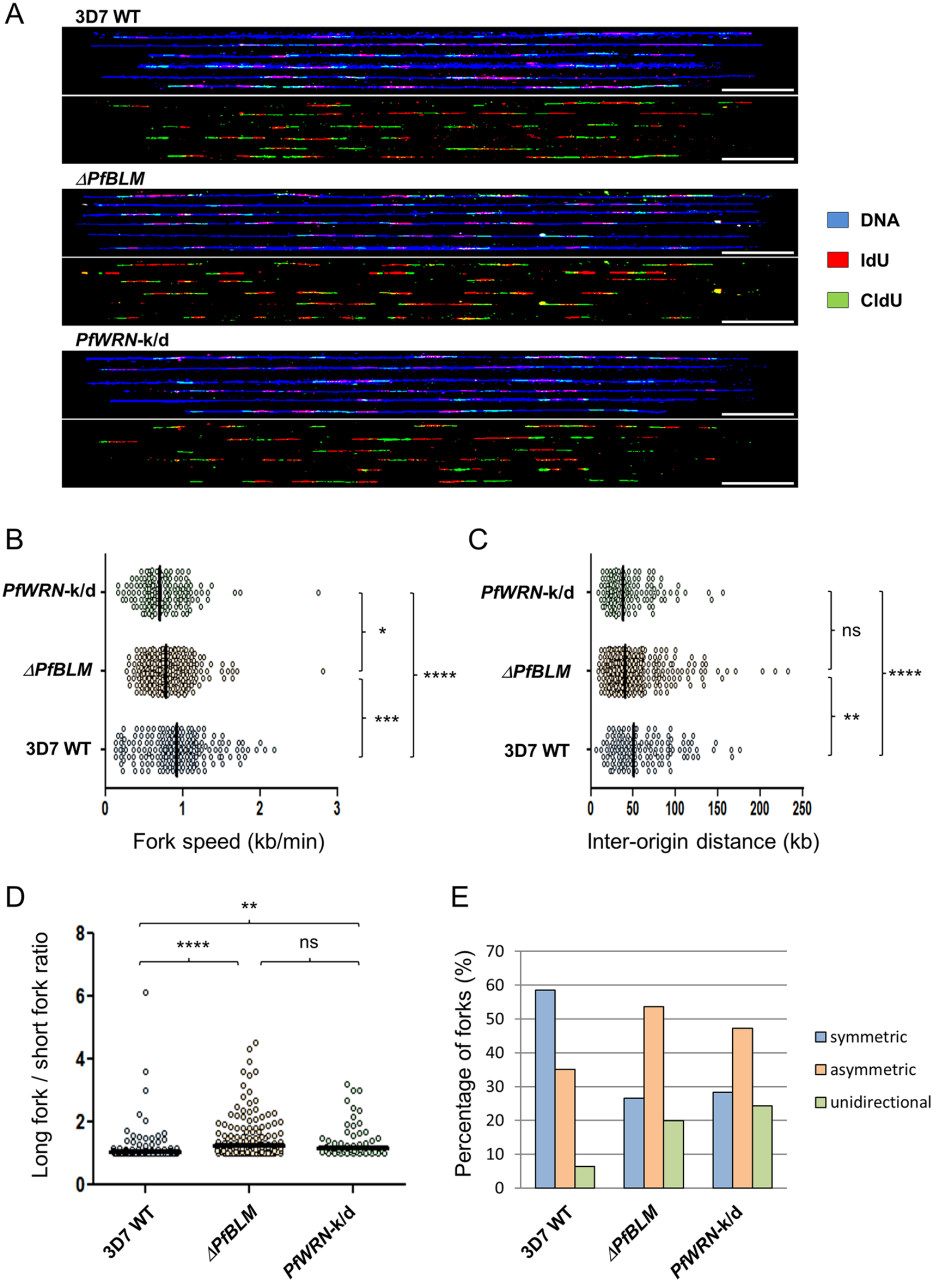
DNA replication parameters in RecQ helicase mutants. (A) Representative DNA fibres from synchronous blood-stage parasites. Upper panel: DNA fibres are in blue, IdU in red and CldU in green; lower panel: the IdU and CldU tracks extracted from the upper panel. 50 kb scale bars are indicated. (B, C and D) Comparative analysis of replication fork speed (B) inter-origin distances (C) and asymmetric forks (long fork to short fork ratios) (D) from synchronous blood-stage parasites. Black bars on dot plots indicate median values. The two-tailed Mann-Whitney test was used to calculate the corresponding P values (ns, not statistically significant P value; * P < 0.05; ** P < 0.01; *** P < 0.001; **** P < 0.0001). (E) Percentage of symmetric, asymmetric and unidirectional replication forks counted in the wildtype and RecQ helicase mutants. The groups were significantly different by Chi-square test (P < 0.00001).

Many of the phenotypes described above could be attributable to failure of RecQ-mutant parasites to resolve the stalling of RNA or DNA polymerases at secondary structures. To test this hypothesis we measured DNA replication dynamics in the mutant lines on a single-molecule level, using immunofluorescent labelling of nascent DNA replication on combed DNA molecules (Fig 4A [23]). In both the RecQ mutant lines, in two independent experiments, replication forks moved more slowly than in the wildtype parent and replication origins fired closer together (Fig 4B and 4C and S16A and S16B Fig). We previously showed that origin spacing positively correlates with fork speed across the course of S-phase [23] and this correlation was apparently maintained in the RecQ mutant lines (S17 Fig). The knockdown of *Pf*WRN had a significantly greater impact on replication fork velocity than *Pf*BLM knockout (Fig 4B, S16A Fig), and replication origins accordingly fired with the closest spacing in the *Pf*WRN-k/d line. Together with the slowing of replication forks, the movement of bidirectional fork pairs on either side of replication bubbles became less symmetric in RecQ mutant lines (Fig 4D, S16C Fig), and ‘unidirectional’ forks having no partner on the opposite side of a bubble became more frequent (Fig 4E, S16D Fig). Unidirectional and asymmetric forks can both be taken as proxies for the amount of replication fork stalling: this was similar in both RecQ mutants, and significantly greater than in the wildtype parent.

### DNA replication dynamics are adversely affected by G-quadruplex binding drugs

**Fig 5:**
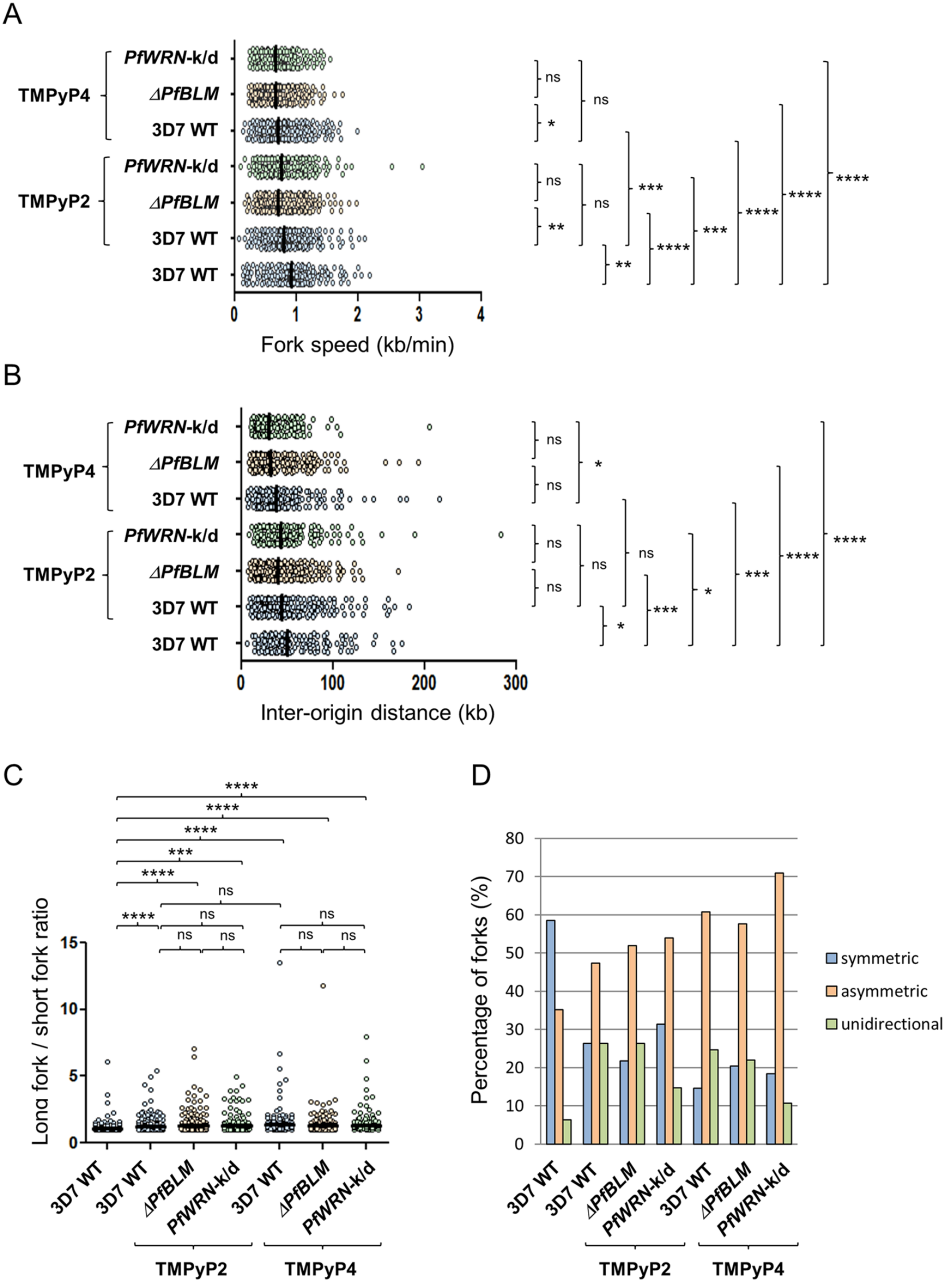
DNA replication parameters in RecQ helicase mutants challenged with G4 stabilising drugs. (A, B and C) Comparative analysis of replication fork speed (A) inter-origin distances (B) and asymmetric forks (long fork to short fork ratios) (C) from synchronous blood-stage parasites, treated with 0.75μM TMPyP4 or TMPyP2. Data from wildtype parasites untreated with drugs are also shown for comparison. Black bars on dot plots indicate median values. The two-tailed Mann-Whitney test was used to calculate the corresponding P values (ns, not statistically significant P value; * P < 0.05; ** P < 0.01; *** P < 0.001; **** P < 0.0001). (D) Percentage of symmetric, asymmetric and unidirectional replication forks counted in the wild type and RecQ helicase mutants challenged with G4 stabilising drugs. The groups were significantly different by Chi-square test (P < 0.00001).

To test whether the phenotypes observed in Fig 4 might be related to polymerase stalling at unprocessed G4 motifs, we repeated the measurements of replication dynamics in all three parasite lines challenged with the G4-binding drugs TMPyP4 and TMPyP2. In wildtype 3D7 parasites, replication dynamics were dramatically affected by these drugs, with a slowing of replication forks and a decrease in the spacing of origins, as well as increased measures of fork stalling (Fig 5A–5D, S18A–S18D Fig). These changes were more severe in TMPyP4, which is the stronger G4-binding drug, than in TMPyP2. Interestingly, the slowing and stalling that had already been observed in RecQ mutant parasites when compared to wildtype 3D7 was not further exacerbated by G4-binding drugs (Fig 5A–5D, S18A–S18D Fig).

## Discussion

We present here the first report on the biological roles of RecQ helicases in *P*. *falciparum* at the genomic and transcriptomic levels. We show that these helicases have far-reaching effects on genome stability and gene transcription, including particular effects on recombination and transcription of the important *var* family of virulence genes.

At the genomic level, we observed a marked difference between the two mutant lines: knockout of *Pf*BLM did not influence the mutation rate, while the knockdown of *Pf*WRN created a highly elevated number of micro-indels and SVs, plus a plethora of unusual recombination patterns never observed in previous clone trees that were generated from wildtype parasite strains [3, 13]. Because the growth rate of the *PfWRN-*k/d parasites is reduced, the true mutation rate could be even higher than the current estimation. Hypermutation at the level detected here is probably deleterious in the wild: no natural mutant in *PfWRN* appears in any sequenced strain and this helicase also appears to be more important for parasite survival *in vitro* than *Pf*BLM, given our failure to obtain a complete *PfWRN* knockout. Indeed, the survival of our *Pf*BLM knockout contradicts a previous report that *Pf*BLM is essential: this was established by treating parasites with a dsRNA against *PfBLM* [30], but no evidence was shown that the dsRNA actually affected the intended gene.

To our knowledge this is the first discovery of a gene directly involved in genome stability in *P*. *falciparum*: a mild mutator phenotype has previously been linked to the mismatch repair factor *Pf*MLH1 [13, 31], but this involved only an elevated SNP rate. By contrast, *Pf*WRN appears to functionally suppress all types of mutations, including small indels, SNPs and in particular, large structural variants. Its role in suppressing structural variants and indels is probably particularly crucial for a genome in which repetitive sequences, and thus risks of DNA polymerase slippage, are common. An increased SNP rate was not necessarily expected, since *Pf*WRN is reported to lack the proofreading exonuclease domain found in human WRN [22], and importantly the SNP datasets were all very small, making changes in SNP rate only marginally significant. Nevertheless, it is possible that an unidentified, divergent exonuclease domain may exist in *Pf*WRN, or that other defects causing an excess of single-stranded DNA could lead to an elevated SNP rate. Otherwise, the phenotypes seen here are consistent with RecQ functions in other organisms: for example, the yeast helicase Sgs1 is required to prevent indels and fragility in triplet repeat regions [32], while metazoan RecQ homologues in species from humans to *C*. *elegans* are required to maintain chromosome integrity and suppress genome instability and aberrant recombination [33–35]. In *P*. *falciparum*, the result appears to be that a RecQ helicase has taken on a specific, and perhaps unique, role in modulating the evolution of a major virulence gene family (albeit with the caveat that any recombination events between other, essential genes, must go unrecorded due to their lethality). Importantly, our clone tree plus whole genome sequencing approach successfully identified RecQ-associated mutations at a resolution never described in other model organisms so far.

**Fig 6:**
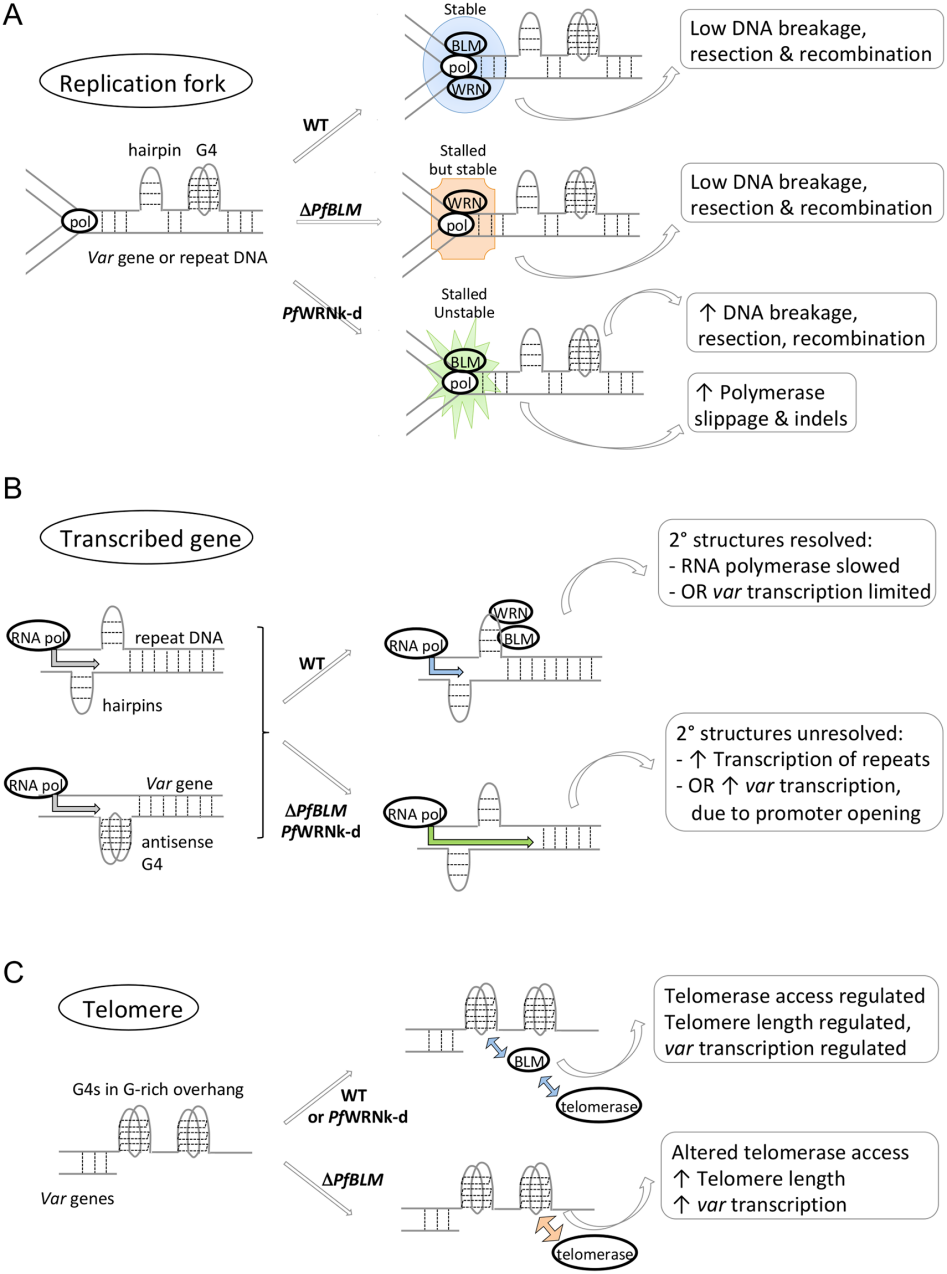
Schematic summarising the roles of RecQ helicases in *P*. *falciparum*. The schematic shows proposed helicase roles at replication forks (A), transcribed genes (B) and telomeres (C).

DNA features such as TRs, LCRs and G4s can all form secondary structures that impede the passage of polymerases, and many of the mutation events seen here correlate with such features: structural variants being particularly associated with PQSs, and to a lesser extent with TRs, while indels are strongly associated with TRs and LCRs. Mammalian RecQ homologs are known to act on stalled replication forks and recombination intermediates [36–38] and the two RecQ homologs in *Plasmodium* spp. presumably act similarly. Our evidence from DNA combing supports stalled replication forks as the mechanism underlying elevated rates of recombination in the *Pf*WRN mutant, but it is curious that only this mutant showed severe genome instability, given that both mutants showed strong replication defects on DNA fibres. In fact, stalled forks may be processed via at least two different routes: a recombinogenic route in the absence of *Pf*WRN and a less recombinogenic route in the absence of *Pf*BLM (Fig 6A). Indeed, the *Pf*WRN mutant showed a specific change in the distance between recombination breakpoints and PQSs, suggesting that replication forks are processed differently when they encounter a G4 in this mutant, with longer lengths of DNA being unwound or resected before a recombination event can occur. This is commensurate with one of the reported roles of human WRN, suppressing resection of DNA breaks [39]. Additionally, the two RecQ helicases may preferentially target particular substrates, such as regressed replication forks, recombination intermediates, telomeric G4s or R-loops: such a ‘division of labour’ occurs amongst the five-member RecQ family in mammals [19], and all these roles must presumably be condensed into a two-member family in *Plasmodium*. Recombinant protein fragments of both *Pf*BLM and *Pf*WRN have been shown to unwind partial-duplex DNA structures [21, 40] but their affinity for other substrates has not been determined and the two helicases are certainly not functionally interchangeable.

As expected, DNA replication was disrupted in wildtype parasites when they were treated with G4-binding drugs, showing that G4s are indeed replication-stalling structures. However, G4s are predicted to occur very rarely (only ~1 per 300kb [9]), so every disrupted replication fork cannot be directly caused by a G4. Instead, a few DNA breaks may induce a wider checkpoint response, slowing down all active forks *in trans* [41]. Alternatively, the TMPyP4/2 drugs may bind to DNA at other locations besides G4s: TMPyP4 is known to be a strong but not highly specific binder of *Plasmodium* G4s [42]. Finally, it is possible that the sequence-based prediction of PQSs severely underestimates the actual G4 density in the genome. However, the idea that replication stalling can induce a general checkpoint response *in trans* is further supported by the observation that in the RecQ mutants, existing disruption to replication dynamics was not further exacerbated by TMPyP4/2, suggesting that it is already at its maximal level in these mutants owing to a constantly active checkpoint response. Almost nothing is currently known about cell cycle checkpoints in *Plasmodium*.

In contrast to this possible *in trans* effect, the effects observed at the transcriptional level are more likely to occur *in cis* on a gene-by-gene basis (Fig 6B). They are probably attributable to impeded RNA transcription when helicase activity is lacking at common DNA features such as hairpins, because the genes deregulated in ring-stage mutant parasites tended to have high levels of repetitive and low-complexity sequence (TRs/LCRs), prone to form such secondary structures. In addition, specific changes were seen in *var* transcription and these may be primarily due to G4s, since *var* genes are enriched in PQSs but are not particularly rich in TRs/LCRs. Deregulated gene expression may be concentrated at the ring stage because other DNA processing proteins such as replicative helicases are not yet upregulated, making the RecQs at their least redundant in prereplicative parasites. Interestingly, a high TR/LCR content was linked primarily to up-regulation rather than down-regulation of transcripts, suggesting that the RecQ helicases normally slow down transcription when they process aberrant DNA structures, and that faster but potentially error-prone transcription can occur in their absence. Again, the effects of *Pf*WRN and *Pf*BLM mutations were largely non-overlapping, which is likewise seen in the transcriptomes of different RecQ mutants in mammalian cells [20].

Although many of the *PfWRN* and *PfBLM* mutant phenotypes showed little overlap, both helicases probably contribute to G4 processing at *var*s because both mutant lines showed altered transcription of PQS-containing *var*s in ring-stage parasites. Notably, not only was the PQSs-encoding subset of *var* genes disproportionately affected, but those *var*s with a PQS on the antisense strand were specifically upregulated. This may be because a persistent antisense G4 can keep the corresponding sense-strand free and thus accessible for transcription. Importantly, this could have a direct impact on the virulence of parasites in human hosts: if the expression or activity of RecQ helicases can vary during human infections, the expression and antigenic variation of the parasite’s primary virulence gene family could vary accordingly. Indeed, RecQ helicase expression can vary *in vivo*: it is higher in parasites from children than from pregnant women (and still lower in cultured 3D7) [43].

Finally, we propose that in addition to the roles of *Pf*BLM in modulating replication and transcription through DNA secondary structures, this helicase could have another role in telomere maintenance and in modulating subtelomeric chromatin structure (Fig 6C). This is evidenced by the lengthening of telomeres and the upregulation of overall *var* gene expression (regardless of PQS association) in the Δ*PfBLM* mutant. Subtelomeric DNA that encodes *var* genes tends to be heterochromatinised [44] and relaxation of this state is expected to boost *var* expression, so *Pf*BLM may have a specific effect in maintaining heterochromatin. This may well be linked to its effect upon telomere maintenance, because the heterochromatic state spreads inwards from the telomeres [45] and telomere repeats are intrinsically G4-rich, with incorrect processing of telomeric G4s deregulating telomerase activity [46–48].

The Δ*PfBLM* phenotype is intriguingly similar to that of a mutant in another chromatin-maintenance protein, the histone deacetylase *Pf*Sir2a [49–51]. Both mutants show telomere lengthening together with overexpression of a fixed set of *var* genes, suggesting that both enzymes affect the integrity of subtelomeric chromatin—possibly in direct collaboration. There is a precedent for this because the human sirtuin SIRT6 acts on telomeric chromatin and facilitates recruitment of human WRN to maintain telomere stability [52]. In *P*. *falciparum* the effect upon telomeres appears to be exerted primarily by *Pf*BLM, but it is important to note that the two *Plasmodium* helicases are not necessarily functional homologs of their mammalian namesakes [21].

Taking our data as a whole, we have detected several overlapping but distinct roles for the *P*. *falciparum* RecQ helicases, as summarised in Fig 6. We divide these into four overall categories: an effect on common DNA secondary structures throughout the genome; a second effect specific to G4-containing variant gene sequences, a third effect on chromatin structure and a fourth on telomere maintenance. Together these effects give rise to complex changes in both genome replication and transcription when the helicases are lacking. This study reveals the *P*. *falciparum* RecQ helicases as important modulators of virulence phenotypes and genome evolution in this major human pathogen.

## Materials and methods

### Parasite culture and transgenic parasites

The 3D7 strain of *P*. *falciparum* was obtained from the Malaria Research and Reference Reagent Resource Center (MR4). Parasites were cultured as previously described [53], in gassed chambers at 1% O_2_, 3% CO_2_, and 96% N_2_. Synchronised parasite cultures were obtained by treatment with 5% D-sorbitol [54]. For cloning by limiting dilution, parasites were diluted to a theoretical concentration of 0.1–2.0 parasites per well of a 96-well plate at 2% haematocrit. Parasites were fed with fresh 0.4% haematocrit medium on days 7 and 14. Positive wells were identified microscopically between days 14 and 21. To disrupt the *PfBLM and PfWRN* loci, gene targeting plasmids were constructed as previously described [55] and transfected into *P*. *falciparum* 3D7. Further detail and primer sequences are given below. For DNA combing experiments, the mutant lines were additionally transfected with plasmids based on the same ‘pTK’ gene-targeting plasmid, and thus expressing thymidine kinase, with a second drug-selectable marker, Blasticidin S-deaminase, cloned in the place of the original dihydrofolate reductase marker. These lines were compared with parental 3D7 parasites that has also been transfected with a pTK plasmid and thus expressed thymidine kinase.

### Plasmid construction and parasite transfection

5’ and a 3’ segments of the *PfBLM* gene (*PF3D7_0918600*) were amplified using the primer pairs *PfBLM*5’F/R and *PfBLM*3’F/R, respectively (see Table 3), and subcloned into the double-selectable gene-targeting pHHT-TK plasmid (MRA-448, MR4) [55]. A plasmid to target the *PfWRN* locus (*PF3D7_1429900*) was similarly constructed using primer pairs *PfWRN*5’F/R and *PfWRN*3’F/R (Table 3). Transgenic parasites were generated by allowing synchronous late-stage parasite cultures to invade erythrocytes pre-loaded with 50–100 μg plasmid DNA as previously described [56]. Plasmid-carrying parasites were positively selected with 5 nM WR99210. Double crossover knockout events were selected from transfected parasite populations by treatment with 20 μM ganciclovir.

**Table 3:** Primers used in this study.

Primer	Sequence (5’-3’)	Use
*PfBLM*5’F	ATCCGCGGGGAGTATTACAAAACAAA	Amplify 5’*PfBLM* segment
*PfBLM*5’R	ACAGATCTCTTAAAGCTTGTATACCT	
*PfBLM*3’F	CATGAATTCCATGCTGGATTAACA	Amplify 3’*PfBLM* segment
*PfBLM*3’R	CATCCTAGGTGCTTCCTCTTTACA	
*PfWRN*5’F	CATCCTAGGTGCTTCCTCTTTACA	Amplify 5’*PfWRN* segment
*PfWRN*5’R	CATAGATCTAGAGTATAGCTTCAGCATC	
*PfWRN*3’F	CTGGAATTC ACACAAACAATATGATACA	Amplify 3’*PfWRN* segment
*PfWRN*3’R	CA CCTAGGTACGTCTCTATAAACGAA	
*PfWRNjp2F*	TCTTCCTTAAAAGGGAAAACAGG	Realtime PCR of *PfWRN* locus
*PfWRNjp2R*	TCCTCTCCTTTGTCCGTAACA	
*PfBLM_F*	TGGATTTCTGGGAAGACCAA	Realtime PCR of *PfBLM* locus
*PfBLM_R*	TCCACTTCTTCCACATCTTCC	

### Southern blotting

Genomic DNA was extracted from parasites using the QIAamp DNA Blood Mini Kit (Qiagen) and digested with restriction enzymes. Digested genomic DNA was resolved on a 1% agarose gel, transferred to GenScreen Plus (PerkinElmer) and hybridised with alkaline phosphatase-labelled probe (AlkPhos Direct Labelling and Detection system, GE Healthcare). For confirmation of mutant lines, DNA was digested with *Sac*II, *Eco*RI and *Hpa*I (Δ*PfBLM*) or *Pml*I and *Sna*BI (*PfWRN*-k/d). For telomere restriction fragment Southern blotting genomic DNA was digested with *Alu*I, *Dde*I, *Mbo*II and *Rsa*I and the membrane was probed with alkaline-phosphatase labelled probe specific for telomeres [57]. ImageJ software was used to quantify telomere length.

### Gene expression analysis

Total RNA was extracted from parasites as previously described [58]. Extracted RNA was treated with DNaseI and cDNA was subsequently synthesised using the iScript cDNA Synthesis Kit (BIO-RAD). cDNA was checked for genomic DNA contamination by PCR across the intron of the gene *PF3D7_0424300*, as previously described [59]. Relative gene expression was determined by real-time PCR using a StepOnePlus Real-TIme PCR machine (ThermoFisher Scientific) and the SensiFAST SYBR Hi-ROX kit (Bioline) on synthesised cDNAs. Cycling conditions were 95°C for 3 minutes, 40 cycles of 95°C for 15 seconds, 54°C for 40 seconds, 60°C for 1 minute. All primers used are either cited elsewhere or listed herein.

Realtime PCR for 3D7 *var* gene transcription was carried out as previously described [50], using the same control genes: five housekeeping genes and also two ring-stage-specific genes. For this analysis, ring-stage RNA was taken from four separate recent clones of each line (4–5 weeks from cloning). Variation between the *var* gene expression patterns in different clones of 3D7 WT, Δ*PfBLM* and *PfWRN*-k/d was quantified as follows. The percentage contribution of each *var* gene to the total *var* RCN in each clone was calculated. Variation in this percentage was then calculated by making pairwise comparisons between all four clones (A-D) of each line, i.e. {|A-B|+|A-C|+|A-D|+|B-C|+|B-D|+|C-D|}. The sum of the ‘variation values’ for all *var* genes in the family was then calculated for each parasite line.

### Parasite growth assays

Growth over one 48h growth cycle was measured by Malaria SYBR Green I-based fluorescence (MSF) assay, essentially as previously described [60]. Trophozoite-stage cultures of all parasite lines being tested were seeded in triplicate into 96-well plates at 1% parasitaemia, 4% haematocrit. The outer wells of each plate were filled with medium to prevent evaporation. Plates were incubated for 48h in a gassed chamber at 37°C. Following this, 100 μL of sample from each well was transferred to plate wells containing 100 μL MSF lysis buffer (20 mM Tris pH 7.5, 5 mM EDTA, 0.008% saponin, 0.8% Triton X-100) supplemented with 0.2 μL mL^-1^ of SYBR Green I (Sigma). After a 1h incubation in the dark at room temperature, SYBR Green I fluorescence was measured using the blue fluorescent module (excitation 490 nm: emission 510–570 nm) of a GloMax multidetection system (Promega). Percentage parasite growth was calculated as follows: 100x[μ_(s)_—μ_(-)_/μ_(+)_—μ_(-)_] where μ_(s)_, μ_(-)_ and μ_(+)_ are the means of the fluorescent readouts from sample wells (μ_(s)_), control wells with 100μM chloroquine (μ_(-)_, representing 0% growth), and control wells with wildtype 3D7 parasites (μ_(+)_, 100% growth).

Growth over two 48h cycles was measured by seeding parasites at 0.1% parasitaemia and then counting the parasitaemia via blood smear microscopy (with a minimum of 100 parasites) at 48h intervals. All growth assays were conducted in triplicate.

Parasite DNA content was measured by two methods: merozoite-counting by microscopy, and flow cytometry. Flow cytometry was carried out on a Guava easyCyte system, using parasites isolated by Percoll and then held for 4h in the egress inhibitor E64 (10μM) before staining with SYBR Green 1, fixing with formaldehyde, and quantifying the fluorescence of 5000 parasites.

### Clone trees and whole genome sequencing

Clone trees, DNA extraction and sequencing were performed as described in [13]. Briefly, cultures of 3D7, Δ*PfBLM* and *PfWRN*-k/d were periodically cloned out by limiting dilution and one clone was arbitrarily picked at each stage for the next round of clonal dilution. At least 2μg of DNA per sample was used for PCR-free library generation at the Wellcome Trust Sanger Institute. 100bp paired-end reads were generated by Illumina sequencing, as previously described [61].

### Detection of SNPs, micro-indels and structural variants

SNPs, micro-indels and structural variants were called as previously described [3, 13]. Briefly, sequencing reads were mapped to the *P*. *falciparum* 3D7 genome with BWA. SAMtools mpileup detected SNPs, GATK UnifiedGenotyper detected micro-indels, DELLY2 detected structural variants (translocations, inversions, duplications and deletions > 300bp). A downstream analysis using R scripts identified *de novo* mutations as genetic variants found in a “progeny” sample but not in its parent. All mutations were visualised with Savant [62]. 563 hits were manually inspected and 393 false hits were discarded (the same false hits were often found in all subclones, hence the relatively large number) (S5A Fig). The False/True call was not biased towards any clone tree (S5B Fig). Translocations (or ectopic recombinations) tend to occur in clusters, i.e. a translocation between two subtelomeres highly increases the chance of detecting further recombinations nearby. The short distance between two breakpoints (sometimes < 100bp) and the repetitive nature of subtelomeres makes it difficult to detect the exact number of recombinations. Therefore multiple recombinations between two subtelomeres within a sample were considered a single mutational event. All 85 clonal genomes are available on the ENA (S1 Spreadsheet).

### RNA-seq

Highly synchronous clonal cultures of 3D7, Δ*PfBLM* and *PfWRN*-k/d were split into 3 separate dishes once invasion was complete (confirmed by light microscopy) and cultured under standard conditions. 3–5 μg total RNA was harvested from each of the 9 cultures, as previously described [58], at the following time points of the subsequent developmental cycle: 14.5–16.5, 24.5–26.5 and 38.5–40.5 h post invasion, giving 3 biological replicates of each line at ring, trophozoite and schizont stages. S19 Fig shows the strength of correlation between biological replicates. A Bioanalyzer Nano chip (Agilent) was used to QC and quantify total RNA. A modified RNA-seq protocol (“DAFT-seq”, Chappell *et al*., in preparation) was used to account for the extreme AT-content of the *P*. *falciparum* transcriptome. Briefly, polyA+ RNA (mRNA) was selected using magnetic oligo-d(T) beads. Reverse transcription using Superscript III (LifeTechnologies) was primed using oligo d(T) primers, then second strand cDNA synthesis included dUTP. The resulting cDNA was fragmented using a Covaris AFA sonicator. A “with-bead” protocol was used for dA-tailing, end repair and adapter ligation (NEB) using “PCR-free” barcoded sequencing adaptors (Bioo Scientific, similar to the method of Korarewa *et al*. [63]). After 2 rounds of SPRI clean-up the libraries were eluted in EB buffer and USER enzyme mix (NEB) was used to digest the second strand cDNA, generating directional libraries. The libraries were quantified by qPCR and sequenced on an Illumina HiSeq2000. Sequence data have been submitted to the ENA database under accession number ERP021698.

TopHat2 [64] was used to map reads against the *P*. *falciparum* 3D7 reference genome. Read counts and fragments per kilobase of transcript per million mapped reads (FPKM) values were calculated for each gene using HT-seq count [65] and Cufflinks [66], respectively. Differential expression was analysed using EdgeR [67], using a threshold of 1.5-fold difference in expression from the wildtype 3D7 (See S4 Spreadsheet). Data plots were created in R and Artemis [68] or Integrated Genome Browser (IGB) were used to visualise mapped reads.

### Analysis of clone tree data

The proximity of all mutations found in the clone trees to PQSs (which were located previously using the tool QGRS mapper) was determined as previously described [9]. Median breakpoint-to-PQS distances were determined for each dataset, together with 95% confidence intervals (C.I.). A significant difference was reported if a particular median fell outside the confidence interval for the control dataset, i.e. the median distance of random simulated breakpoints from PQSs, taken from our previous study [9].

The percentage content of tandem repeats and low-complexity regions (TRs and LCRs) in the 1kb surrounding each mutation was calculated after downloading the locations of these two features throughout the genome from PlasmoDB.org. PlasmoDB defines TRs as previously described [69] and LCRs according to the DUST algorithm (for details see [70]). Overlapping regions of each type were amalgamated into single blocks to avoid double-counting them. The mean TR and LCR content of 1kb windows across the whole genome was calculated, as well as the mean TR and LCR content of coding genes (i.e. the number of TR or LCR base-pairs that overlapped with each gene-coding region, divided by the total gene length to yield the percentage TR/LCR content of that gene). The mean TR/LCR content of the 1kb windows surrounding each set of mutations was then compared to the whole-genome average, and significant differences were assessed by 2-tailed t-test.

### Analysis of RNA-seq data

To determine the proximity of differentially expressed (DE) genes to PQSs, a similar approach was taken as for the clone-tree data, using the algorithm detailed in Supplementary Methods. In brief, the distances from each end of a gene to all the PQSs that share its chromosome were calculated and the smallest distance was recorded. For each set of DE genes, their mean and median proximity to PQSs was then calculated. As a comparator, the PQS proximity of all genes in the genome was calculated. The statistical significance of differences between the proximity of DE gene sets and the proximity of all genes in the genome was assessed by comparing means via two-tailed t-tests.

The mean TR/LCR content of each DE gene set was compared to that of all genes in the genome, as described above for 1kb windows around clone-tree mutations. Statistically significant differences were assessed by two-tailed t-tests.

### DNA molecular combing

Agarose plugs for DNA molecular combing were prepared as previously described [23]. In brief, two modified nucleosides were used to label parasites: iodo-deoxyuridine (IdU, Sigma) and chloro-deoxyuridine (CldU, Sigma). Parasites were sequentially labelled for 10 minutes with 25 μM IdU, then for 10 minutes with 200 μM CldU, added directly to the culture without intermediate washing. After labelling, the cultures were immediately placed on ice to stop DNA replication; parasites were removed from cells using saponin and embedded in agarose as previously described. The drugs 5,10,15,20-tetra-(N-methyl-4-pyridyl)porphine (TMPyP4) and 5,10,15,20-tetra-(N-methyl-2-pyridyl)porphine (TMPyP2) (Frontier Scientific) was added at 0.75μM as in previous work [27], together with the first (IdU) label.

Two independent labelling and combing experiments were performed, using wildtype, Δ*PfBLM and PfWRN*-k/d lines (all three lines additionally expressing thymidine kinase), synchronised in parallel to a ~6hr window by double-sorbitol treatment as previously described [23]. Data from the second experiment are shown in S16 and S18 Figs; DNA replication parameters from both experiments are collated in S2 Table. Stage-matching was confirmed by counting the morphology of 50 parasites in each line by blood-smear microscopy (S20 Fig). The three parasite lines were then harvested in parallel at the latest stage of the replicative period, measured in our previous work as ‘Stage 3’ (predominantly schizonts and some late trophozoites).

DNA molecular combing and immunofluorescent labelling of replication tracks was conducted exactly as previously described [23]. Antibodies were: anti-ssDNA antibody (1/100 dilution, Chemicon), mouse anti-BrdU antibody (1/20 dilution, clone B44 from Becton Dickinson), rat anti-BrdU antibody (1/20 dilution, clone BU1/75 (ICR1) from Sera Lab), which recognize IdU and CldU respectively. Secondary antibodies were goat anti-rat antibody coupled to Alexa 488 (1/50 dilution, Molecular Probes), goat anti-mouse IgG1 coupled to Alexa 546 (1/50 dilution, Molecular Probes), and goat anti-mouse IgG2a coupled to Alexa 647 (1/100 dilution, Molecular Probes). Coverslips were mounted in Prolong Gold Antifade (Molecular Probes) before image acquisition via a fully motorized Leica DM6000 microscope equipped with a CoolSNAP HQ2 1 CCD camera and controlled by MetaMorph (Roper Scientific). Statistical analyses of inter-origin distances and velocities of replication forks were performed using Prism 5.0 (GraphPad).

### Statistical analysis of DNA fibre data

Data were analysed as previously described [23]. In brief, replication fork velocity was estimated on individual forks displaying an IdU track flanked by a CldU track; only intact forks were analysed, as ascertained by DNA counterstaining. Fork asymmetry was calculated as the ratio of the longer track over the shorter track in pairs of progressing divergent forks. A longer fork/shorter fork ratio >1 indicates asymmetry. Inter-origin distances were measured as the distance (in kb, from the stretching factor of 2 kb/μm) between the centres of two adjacent progressing forks located on the same DNA fibre. DNA replication parameters generally do not display a Gaussian distribution [71] so statistical comparisons were carried out using the nonparametric Mann–Whitney two-tailed tests.

## Supporting information

S1 Fig The *BLM* gene is disrupted by double homologous recombination. (A) The *PfBLM* gene is knocked out by a double-crossover event. Southern blot analysis of genomic DNAs from untransfected 3D7 and Δ*PfBLM* 3D7 parasites, digested with *Sac*II, *EcoR*I and *Hpa*I. The Southern blot was probed with the 3’ flank of the *PfBLM* gene used in the targeting plasmid. A schematic of the digestion pattern and probe location is shown below. Samples are untransfected 3D7 (3D7 WT), transfected parasites prior to ganciclovir selection (Δ*PfBLM* M) showing mixed populations of knockout and untargeted parasites, transfected parasites after ganciclovir selection (Δ*PfBLM* G) and Δ*PfBLM* parasite lines cloned from Δ*PfBLM* G. (B) RNA-seq data showing disruption of *PfBLM* gene. Reads from *ΔPfBLM* and 3D7 WT trophozoites-stage parasites were aligned to the *PfBLM* locus, both in the native 3D7 genome (left side) and in its disrupted form, which was provided as a modified reference genome for alignment of the RNA-Seq data (right side). Each image displays stacks of aligned reads. In 3D7 WT parasites there is coverage across the whole of the *PfBLM* open reading frame (ORF) but no coverage of the *hDHFR* gene in the modified locus because this cassette does not exist in the WT genome. In contrast, RNA from *ΔPfBLM* parasites shows transcription across the entire inserted *hDHFR* cassette, and zero coverage over the small central region of the *PfBLM* ORF that was excised by the double homologous recombination event inserting the cassette.
(PDF)

S2 Fig The *WRN* gene has undergone disruption via plasmid insertion. (A) Disruption of the *PfWRN* gene. Southern blot analysis of untransfected 3D7 and *PfWRN-*targeted parasites. Genomic DNAs were digested with *Pml*I and *SnaB*I. The Southern blot was probed with the *PfWRN* 5’ flank used in the targeting plasmid. A schematic of the digestion pattern and probe location(s) is shown below, demonstrating how a two-plasmid concatamer has integrated within the *WRN* locus. Lane 1, *PfWRN* targeting plasmid (P); lane 2, untransfected wildtype 3D7 (3D7 WT); lanes 3 and 4, mixed populations of transfected parasites after selection with ganciclovir (*PfWRN*-k/d M) (the population in lane 4 has additionally been cycled off WR for one month); lanes 5–8,clonal *PfWRN*-k/d parasites after negative selection with ganciclovir (*PfWRN*-k/d clones). (B) Whole genome sequencing evidence for the nature of the locus in *PfWRN*-k/d. Upper panel: sequencing reads from *PfWRN*-k/d and 3D7 mapped to the 3D7 reference genome. A large increase of coverage can be seen at the 5’ and 3’ ends of *PfWRN* in the transfected line *PfWRN*-k/d. Lower panel: Two-plasmid concatamer sequence was inserted in the 3D7 reference genome and all reads were re-mapped on this modified reference genome. Each plasmid domain is labelled, with a “-2” to indicate the second copy of the plasmid. Reads from *PfWRN*-k/d now match this modified reference sequence. Note that reads mapping to human genes such as *hDHFR* were automatically discarded, as per Sanger Institute policy. (C) RNA-seq data showing disruption of *PfWRN* gene. As in S1B Fig, reads from *PfWRN*-k/d and 3D7 WT were aligned to the *PfWRN* locus, both in the native 3D7 genome (top) and in its disrupted form, provided as a modified reference genome (bottom). Each image displays stacks of aligned reads. In 3D7 WT parasites there is coverage across the whole of the *PfWRN* open reading frame (ORF) but no coverage of the plasmid sequences in the modified locus because they does not exist in the WT genome. By contrast, RNA from *PfWRN*-k/d parasites shows transcription across the entire inserted plasmids, confirming their presence, although it is transcription of the TK negative-selection markers is notably low, consistent with these genes being largely silenced. The truncated WRN gene that is reconstituted at the 3’ end of the modified locus (ORFs are shown as yellow bars) is transcribed at a low level, consistent with RT-PCR data shown in Fig 1.
(PDF)

S3 Fig The cell cycle in RecQ mutant lines is normal but marginally fewer merozoites are produced per schizont. (A) Cell cycle of the Δ*PfBLM*, *PfWRN-*k/d and parent lines, assessed as previously described [72]–i.e. tight synchronisation of parasites at early rings and then staging of 100 parasites by morphology every 8h. No major or consistent change in the length of each morphological stage was observed: representative data shown from one of three biological replicates. (B) Numbers of merozoites per mature segmented schizont in the Δ*PfBLM*, *Pf*WRN-k/d and parent lines. Approximately 20 schizonts were counted in each line. Differences did not attain statistical significance by one-tailed t-test, but merozoite numbers trended down in both mutants, approaching significance (p = 0.07) in *PfWRN-*k/d. (C) DNA content of mature schizonts in the Δ*PfBLM*, *Pf*WRN-k/d and parent lines measured by SYBR Green 1 DNA staining and flow cytometry (5000 parasites per sample). Dotted line marks the peak of fluorescence in the parent line, for comparison with that in each mutant. (D) Data from the PlasmoGEM project (http://plasmogem.sanger.ac.uk/phenotypes) showing the growth phenotypes of *P*. *berghei BLM* and *WRN* knockouts.
(PDF)

S4 Fig Clone trees. Clone trees were built by regularly subcloning individual parasites from the 3D7 wild-type (A), Δ*PfBLM* (B) and *PfWRN*-k/d (C) lines. Asterisks on the time line indicate the day of the cloning by limiting dilution. Note that the *PfWRN*-k/d parental line was cloned out before generating the first generation of subclones. By contrast, the first generation of 3D7 and Δ*PfBLM* subclones cannot be used to calculate mutation rates as the mutations identified within these genomes would have occurred before the start of the clone tree.
(PDF)

S5 Fig Validation of micro-indels. (A) VQSlod score (from GATK’s VQSR, y-axis) is plotted versus the number of reads indicating the putative micro-indel. If the read pileup visualised in Savant did not unequivocally show an indel in a progeny sample and its absence in its parent, it was labelled “False”, as described in [3]. As expected, hits with higher VQSlod score and number of Alt reads were more likely to be “True”. (B) Scatter plot as in (A), displaying only the “True” micro-indels hits, with no bias towards any clone tree. In both graphs the X-axis is on a log scale.
(PDF)

S6 Fig Characterisation of micro-indels. (A, B, C) The types and chromosomal locations of micro-indels were similar in all clone trees. (D, E, F) The proportion of indel length divisible by 2 or 3 was comparable with previous clone trees [3]; however, there was a relatively high proportion of ‘indivisible by 3’ micro-indels (6 out of 12) in exons from the *PfWRN*-k/d line. These lead to frameshifts, but as expected they were found in non-essential genes. (G) As a convention, micro-indels are left-aligned in a repetitive region, i.e. the inserted / deleted sequence is upstream of the repeat. We analysed the 15bp downstream sequences into 3 categories: homorepeat ([A]^n^ or [T]^n^), TA repeat ([TA]^n^) or ‘Complex’ for any other type of repeats. Homorepeats were proportionally 3.1 times more common in *PfWRN*-k/d compared to 3D7.
(PDF)

S7 Fig Recombination between group A and group B *var* genes, or with a pseudogene. (A) Example of a recombination between tail-to-tail group A and group B *var* genes identified in subclone WRN-2e. In the upper panel, the yellow arc is reads with one end mapping to *PF3D7_0800100* and the other to *PF3D7_0800200*. The coverage is doubled over the same region. Thus in our model (lower panel) both parental *var* genes are still present, with an extra chimeric pseudogene sequence made from the 3’ ends of *PF3D7_0800100* and *PF3D7_0800200*. (B) The conserved pseudogene *var1csa* (*PF3D7_0533100*), with an upstream A sequence, is located at the very end of chromosome 5. In subclone WRN-1g, translocation reads indicate a recombination with *PF3D7_0400400*, a group A *var* gene located near the start of chromosome 5. This resulted in a chimeric pseudogene *0400400-Var1*. A *rif* and a group B *var* genes got deleted in the process.
(PDF)

S8 Fig Recombination between an internal and a subtelomeric *var* gene. In subclone WRN-2g, we detected a double recombination between the exon 2s of subtelomeric group B *var PF3D7_1100100* and internal group B *var PF3D7_1240400*. Note that an odd number of cross-over events would have led to a fusion between the two chromosomes, likely non-viable nor detectable.
(PDF)

S9 Fig Inversion within the invasion-related genes *RH2a* and *RH2b*. *Rh2a* and *Rh2b* (reticulocyte binding protein homologue) sequences show 91.8% identity and are located on opposite strands in head-to-head configuration, thus they are almost mirror-image of each other. They are both implicated in binding to the erythrocytes for invasion [73]. In subclone WRN-2b reads (designated by black arrows) indicate an inversion spanning 18kb centred halfway between the two genes. This unusual event might be reminiscent of how one gene was originally generated from the other.
(PDF)

S10 Fig Deletion/Duplication hotspot in *Pf11-1* and *LSA1*. (A) Read coverage of the liver-stage antigen (*LSA1*, *PF3D7_1036400*). Four subclones with a mutation are highlighted in bold. (B) Read coverage over the *Pf11-1* gene (*PF3D7_1038400*). Eight mutant subclones are highlighted. WRN-1a is the parent of the second generation, thus all progeny subclones inherited the *Pf11-1* deletion present in WRN-1a as expected.
(PDF)

S11 Fig RecQ helicases influence G-quadruplex-related phenotypes in *P*. *falciparum*. (A) Drug sensitivity of Δ*PfBLM* and *PfWRN*-k/d parasite lines to the G4-stabilising drugs TMPyP2 and TMPyP4. Mean IC50 values (μM) of TMPyP2 and TMPyP4 from 3 independent assays are shown. Δ*PfBLM* was significantly more sensitive to both TMPyP2 (p = 0.032) and TmPyP4 (p = 0.016). Error bars show standard error of the mean; statistical significance was determined using one-tailed t-tests (*, p<0.05). (B) Telomere restriction fragment (TRF) Southern blot showing telomere lengths in genomic DNA from 3D7 WT clones and Δ*PfBLM* clones. The empty lanes contained DNA ladder, which must be regularly interspersed between lanes to allow accurate measurement of the median DNA fragment size in adjacent telomere smears. The graph shows median telomere lengths calculated from this blot: ImageJ software was used to determine the median point of each smear. Δ*PfBLM* clones have significantly longer telomeres than wildtype 3D7 clones (p = 0.0001, two-tailed t-test). Data from one of two replicate TRF Southern blots are shown. (C) TRF Southern blot displaying telomere lengths from 3D7 WT and *PfWRN*-k/d clones. The graph shows median telomere lengths calculated from this blot using ImageJ software. Telomere length in *PfWRN*-k/d clones does not significantly differ from telomere length in 3D7 WT clones (p = 0.24, two-tailed t-test). Data shown are from one of two replicate TRF Southern blots.
(PDF)

S12 Fig Genes deregulated in the two RecQ mutants lines show only limited overlap at ring, trophozoite and schizont stages. Venn diagrams showing the numbers of genes differentially expressed in Δ*PfBLM* (blue circles) and *PfWRN-*k/d (pink circles) at each time point (R, rings; T, trophozoites; S, schizonts). Percentage overlap is calculated as the number of genes that are in common (deregulated in both Δ*PfBLM* and *PfWRN-*k/d) divided by the total number of deregulated genes in the two lines.
(PDF)

S13 Fig Genes maximally expressed in rings, trophozoites or schizonts do not differ in their content of tandem repeats and low-complexity regions. (A) Box-plot shows the percentage tandem-repeat (TR) content of all genes in the genome, versus that of those genes expressed at 80–100% maximum levels in rings (R, n = 1447), trophozoites (T, n = 1325) and schizonts (S, n = 1498). These gene sets were derived from transcriptomic data from [74], available in PlasmoDB [75]. Timepoints within this dataset were chosen to match the time-windows harvested for RNA-seq in RecQ mutants: 8-16h hpi (R), 24-30h (T) and 40-48h (S). Lines indicate medians, box and whiskers indicate interquartile and full ranges. (B) Table shows mean TR content for each gene set: there was no significant difference between the R, T, and S datasets as assessed by ANOVA. Interestingly, the most highly-expressed genes at all three stages have a higher TR content than the average for all genes in the genome (statistically significant differences as tested by 2-tailed T-test). However, all three differences are only about half as large as the difference between all-genes and the gene sets upregulated in ring stages of RecQ mutant lines (shown for comparison). The table also shows the TR content of *var* genes, grouped into all *var*s, and *var*s containing a PQS on the sense or antisense strand: in no group is the TR content different from that of all ring-stage genes. (C) Box-plot as in (A), showing the percentage low-complexity-region (LCR) content of the same gene sets. (D) Table shows mean LCR content for each gene set: there was no significant difference between the R, T, and S datasets as assessed by ANOVA. The most highly-expressed genes at all stages have a lower LCR content than the average for all genes in the genome. The table also shows the LCR content of *var* genes, which is lower than that of all ring-stage genes.
(PDF)

S14 Fig RecQ helicases influence var gene transcription. (A) *Var* gene expression patterns in four clones of each line: WT 3D7 (clones 1–4), Δ*PfBLM* (clones 5–8) and *PfWRN*-k/d (clones 9–12). Genes are grouped according to their upstream (*ups*) classification, A-E. Each segment of the pie chart shows the expression level of a single *var* gene and the total volume of the pie chart is proportional to the total level of *var* gene transcription. (B) Total level of *var* gene expression for WT 3D7, Δ*PfBLM* and *PfWRN*-k/d clones. Total transcript level is significantly higher in Δ*PfBLM* clones than in 3D7 WT clones (p = 0.0015, two-tailed t-test). (C) The level of *var* gene expression from PQS-containing *var* genes expressed as a percentage of the total *var* gene expression. This percentage is significantly higher in *PfWRN*-k/d clones when compared to 3D7 WT clones (p = 0.0392, two-tailed t-test). (D) Where *var* genes contain a PQS (in their coding or upstream regions), the PQS can occur on either the sense or antisense strand. The level of *var* gene expression from PQS-containing *var* genes which have a PQS on the antisense strand is expressed as a percentage of the total level of PQS-containing *var* gene expression. In Δ*PfBLM* clones compared to 3D7 WT clones a significantly higher proportion of PQS-containing *var* gene transcription occurs in genes where the PQS is on the antisense strand (p = 0.0068, two-tailed t-test).
(PDF)

S15 Fig Individual var gene expression levels in WT 3D7, Δ*PfBLM* and *PfWRN*-k/d clones. Graphs showing the relative copy number (RCN) of each individual *var* gene in four WT 3D7 (A), Δ*PfBLM* (B) and *PfWRN*-k/d (C) clones. Genes are grouped according to their upstream (*ups*) classification, A-E. The numbers in the graph titles (1–12) correspond to the numbers of the pie charts displayed in S14 Fig. (D) Quantification of the variation in *var* expression patterns between the 4 clones of each line shown in (A-C).
(PDF)

S16 Fig DNA replication parameters in RecQ helicase mutants (replicate experiment). (A, B and C) Comparative analysis of replication fork speed (B) inter-origin distances (C) and asymmetric forks (long fork to short fork ratios) (D) from synchronous blood-stage parasites, in an experiment conducted separately from the experiment illustrated in Figs 4 and 5. Black bars on dot plots indicate median values. The two-tailed Mann-Whitney test was used to calculate the corresponding P values (ns, not statistically significant P value; * P < 0.05; ** P < 0.01; *** P < 0.001; **** P < 0.0001). (D) Percentage of symmetric, asymmetric and unidirectional replication forks counted in the wild type and RecQ helicase mutants. The groups were significantly different by Chi-square test (P = 0.00605).
(PDF)

S17 Fig Inter-origin distances and fork velocities are positively correlated. Positive correlation between median inter-origin distances (IODs) and median fork velocities in wild type and RecQ mutants, unchallenged and challenged with G4 stabilising drugs. Coefficient of determination (R^2^) is indicated. (A) First DNA combing experiment, shown in Figs 4 and 5 (B) Second DNA combing experiment, shown in S16 Fig.
(PDF)

S18 Fig DNA replication parameters in RecQ helicase mutants challenged with G4 stabilising drugs (replicate experiment). (A, B and C) Comparative analysis of replication fork speed (A) inter-origin distances (B) and asymmetric forks (long fork to short fork ratios) (C) from synchronous blood-stage parasites, in an experiment conducted separately from the experiment illustrated in Figs 4 and 5. Black bars on dot plots indicate median values. The two-tailed Mann-Whitney test was used to calculate the corresponding P values (ns, not statistically significant P value; * P < 0.05; ** P < 0.01; *** P < 0.001; **** P < 0.0001). (D) Percentage of symmetric, asymmetric and unidirectional replication forks counted in the wild type and RecQ helicase mutants challenged with G4 stabilising drugs. The groups were significantly different by Chi-square test (P = 0.0025).
(PDF)

S19 Fig Data from replicate RNA-seq experiments are highly reproducible at each timepoint. Scatter plots show the correlation in FPKM reads in each of three replicate RNA-seq experiments conducted at each of three timepoints (rings, R; trophozoites, T and schizonts, S) in the three lines 3D7 wildtype, ΔPfBLM and PfWRN-k/d.
(PDF)

S20 Fig Developmental stages of parasites harvested for DNA combing. 50 parasites were counted from each line and classified by morphological stages, then the cultures were labelled and harvested in parallel for DNA combing. Staging is shown in experiment 1 (A) and experiment 2 (B). Parasites were classified as: ER small rings, LR large rings, ET early trophozoites (less than half the width of host cell), MT middle trophozoites (more than half the width of, but not entirely filling, host cell), LT late trophozoite (parasite filling all or nearly all of host cell), ES early schizont (discrete nuclear masses visible within parasite), LS late schizont (defined merozoites visible). Parasites were harvested when they were predominantly early schizonts with some late schizonts and late trophozoites.
(PDF)

S1 Table Deregulated genes in RecQ helicase mutants are associated with sequences having the potential to form unusual DNA structures. For each set of deregulated genes in rings, trophozoites and schizonts of the RecQ helicase mutants, mean and median distance-to-nearest-PQS, tandem-repeat content and low-complexity-region content are shown. These values are plotted in Fig 4.
(PDF)

S2 Table DNA replication parameters (column statistics) in RecQ helicase mutants unchallenged and challenged with G4 stabilising drugs. The parameters from two independent experiments of DNA molecular combing are shown.
(PDF)

S1 Spreadsheet Assembled mutation data from clone trees. Sheet 1: Indicates, for each sample, the percentage of the 3D7 reference genome that is covered by at least 5 or 10 reads. European Nucleotide Archive accession numbers are given for 85 3D7-derived genomes. Sheet 2: All Base Pair Subsitutions (BPS) identified, with a summary table of the type of substitutions. Sheet 3: All micro-indels identified, with summary tables per clone and per generation. Sheet 4: All Structural Variants identified, with summary tables per clone and per generation. Sheet 5: All Structural Variants within *var* genes, including the domain names where the recombination occurred, and the precise coordinates of the ‘Identity Block’ (sequence 100% identical between the two recombining var genes in which the cross-over must have occurred). Sheet 6: Mutation rate calculations.
(XLSX)

S2 Spreadsheet Lists of raw read counts for all RNA-seq samples. Sample names are displayed in column headers. R, T or S in the sample name refers to a ring, trophozoite or schizont time point respectively. 1, 2 or 3 in the sample name refers to the replicate number.
(XLSX)

S3 Spreadsheet Sets of genes deregulated in RecQ helicase mutants. Lists of differentially expressed genes identified by RNA-seq analysis in Δ*PfBLM* or *PfWRN-*k/d parasites. The first sheet lists the numbers of up- and down-regulated genes in each mutant. The next 6 sheets are labelled BLM or WRN, with R, T and S referring to a ring, trophozoite or schizont time point respectively: the genomic location and product description of each gene is listed. GO terms obtained from PlasmoDB.org for each gene set are also listed in a separate set of 6 sheets.
(XLSX)

S4 Spreadsheet Statistics for deregulated genes in RecQ helicase mutants. Statistics pertaining to all deregulated genes in Δ*PfBLM* or *PfWRN-*k/d parasites compared to wildtype 3D7: sheets are labelled BLM or WRN, with R, T and S referring to a ring, trophozoite or schizont time point respectively. LogFoldChange, log_2_ fold change in expression cf wildtype; P-value, statistical significance of the differential expression. The cutoff for fold-change in this analysis was set at 1.5 and only genes with a fold-change of above 1.5 are listed. In all cases, P-value was below 0.05 and False Discovery Rate was also below 0.05.
(XLSX)

S1 Method (DOCX)
