# Supplementary figures and images for: RecQ helicases in the malaria parasite Plasmodium falciparum affect genome stability, gene expression patterns and DNA replication dynamics
Source: PLoS Genet. 2018 Jul 2;14(7):e1007490. doi: 10.1371/journal.pgen.1007490 (PMC6044543; doi:10.1371/journal.pgen.1007490)

Figure S1

A

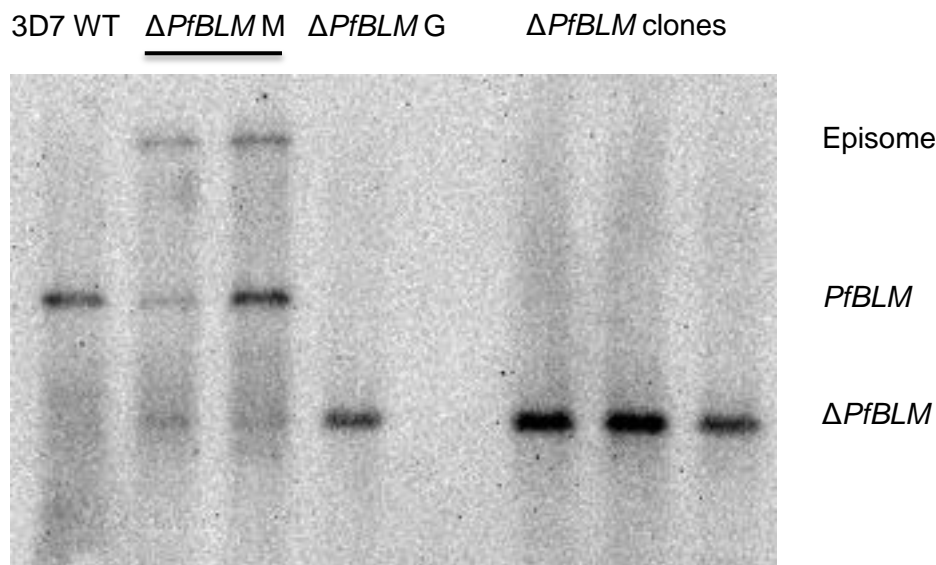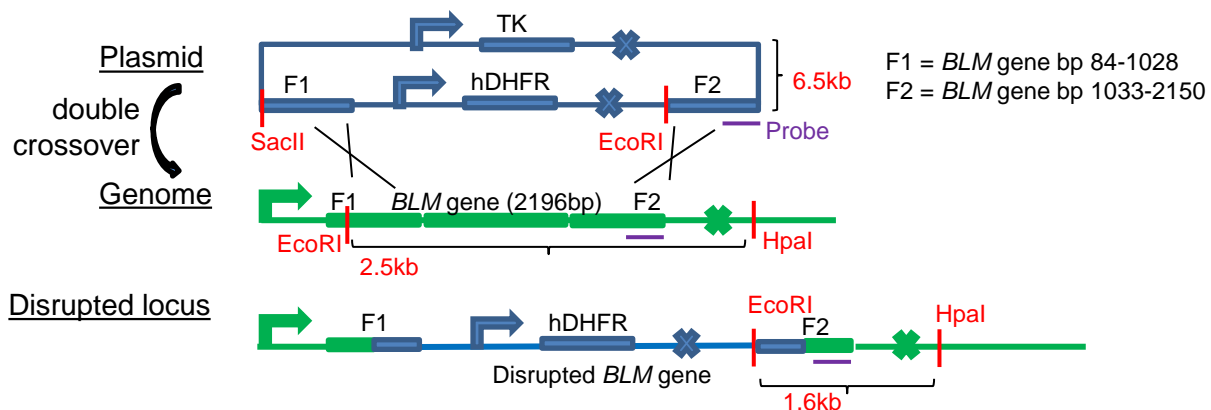

B

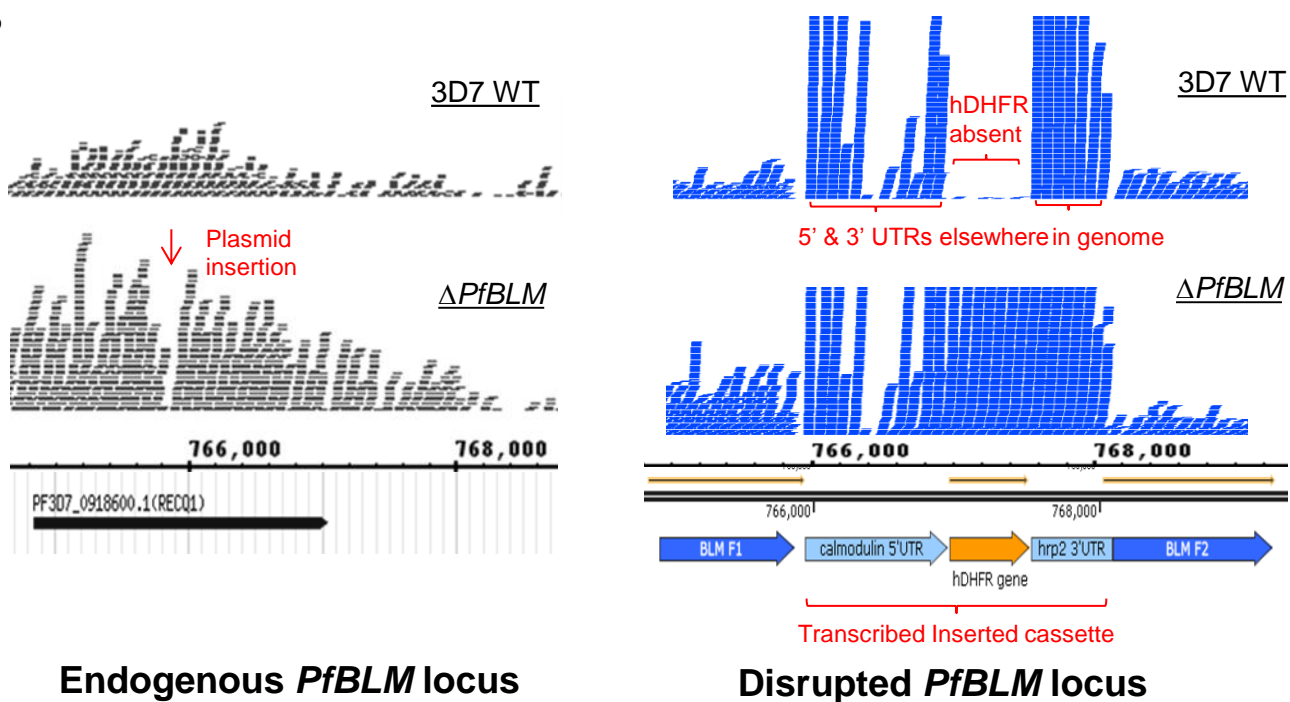

Supplement: S1 Fig — (A) The PfBLM gene is knocked out by a double-crossover event. Southern blot analysis of genomic DNAs from untransfected 3D7 and ΔPfBLM 3D7 parasites, digested with SacII, EcoRI and HpaI. The Southern blot was probed with the 3’ flank of the PfBLM gene used in the targeting plasmid. A schematic of the digestion pattern and probe location is shown below. Samples are untransfected 3D7 (3D7 WT), transfected parasites prior to ganciclovir selection (ΔPfBLM M) showing mixed populations of knockout and untargeted parasites, transfected parasites after ganciclovir selection (ΔPfBLM G) and ΔPfBLM parasite lines cloned from ΔPfBLM G. (B) RNA-seq data showing disruption of PfBLM gene. Reads from ΔPfBLM and 3D7 WT trophozoites-stage parasites were aligned to the PfBLM locus, both in the native 3D7 genome (left side) and in its disrupted form, which was provided as a modified reference genome for alignment of the RNA-Seq data (right side). Each image displays stacks of aligned reads. In 3D7 WT parasites there is coverage across the whole of the PfBLM open reading frame (ORF) but no coverage of the hDHFR gene in the modified locus because this cassette does not exist in the WT genome. In contrast, RNA from ΔPfBLM parasites shows transcription across the entire inserted hDHFR cassette, and zero coverage over the small central region of the PfBLM ORF that was excised by the double homologous recombination event inserting the cassette. (PDF) [file pgen.1007490.s001.pdf]

Figure S2

A

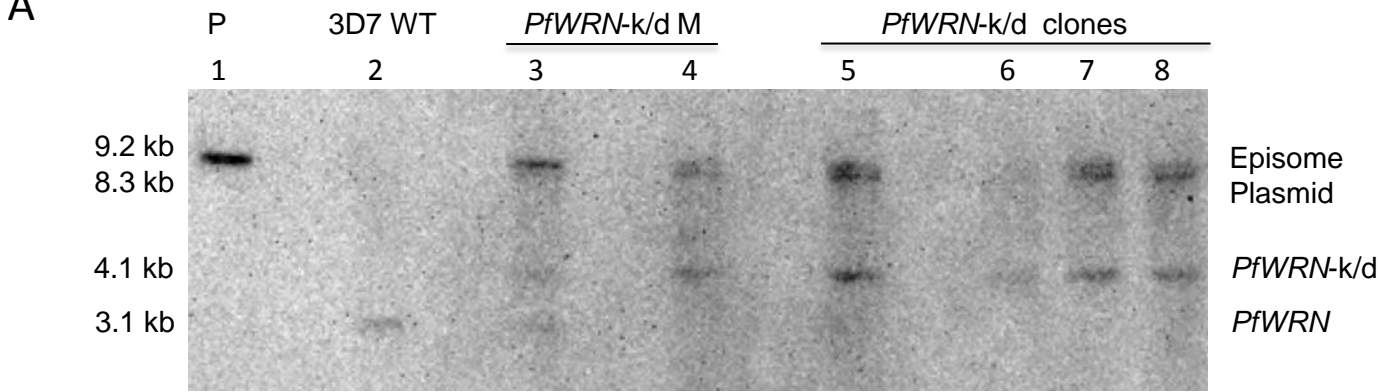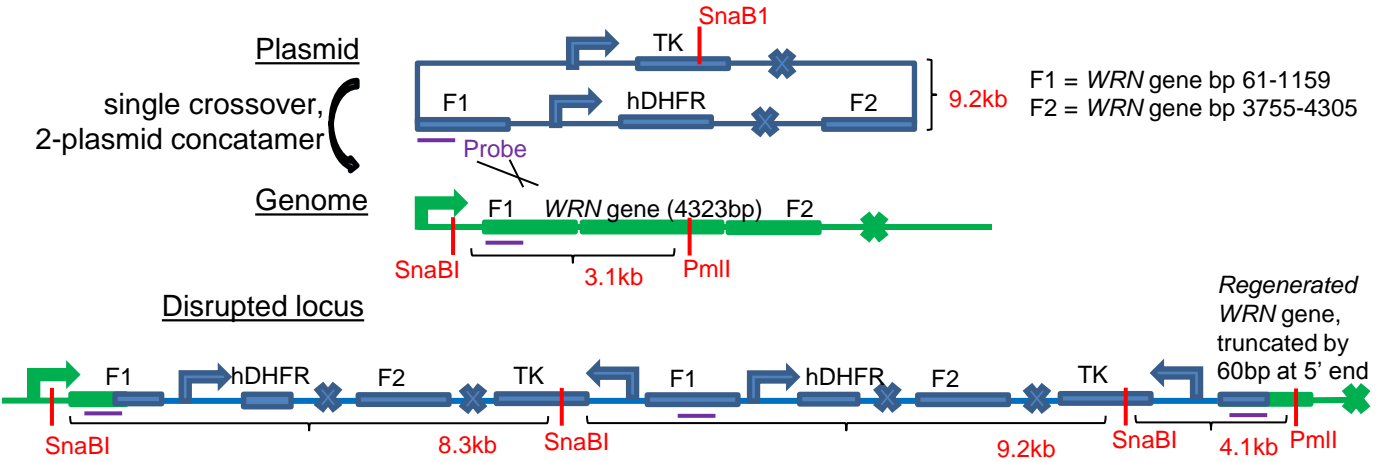

B

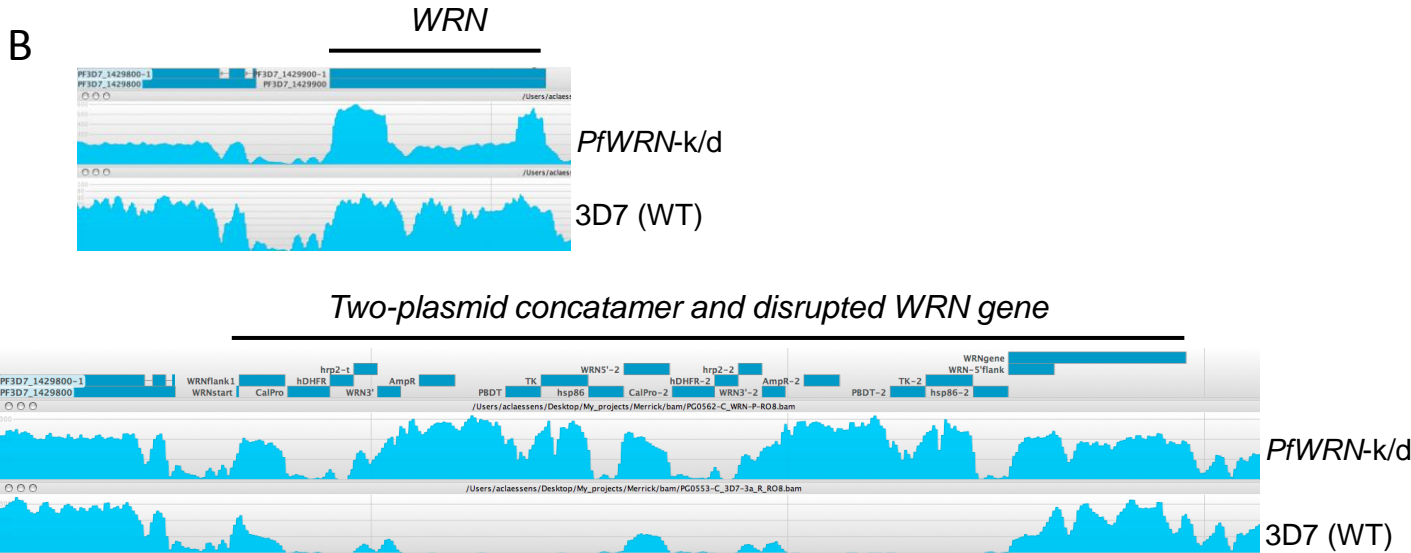

Figure S2 (cont.)

C

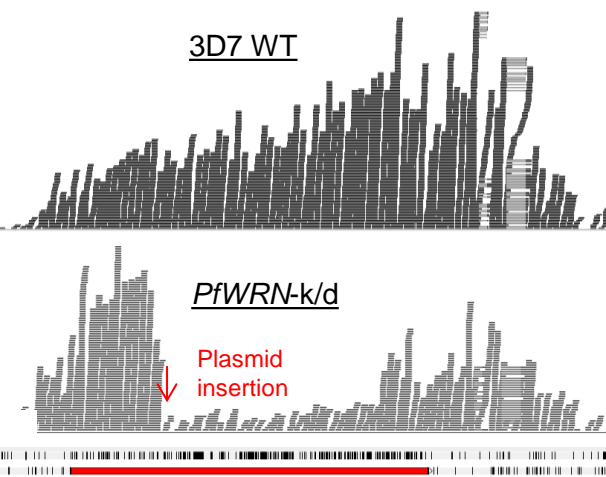

Endogenous *PfWRN* locus

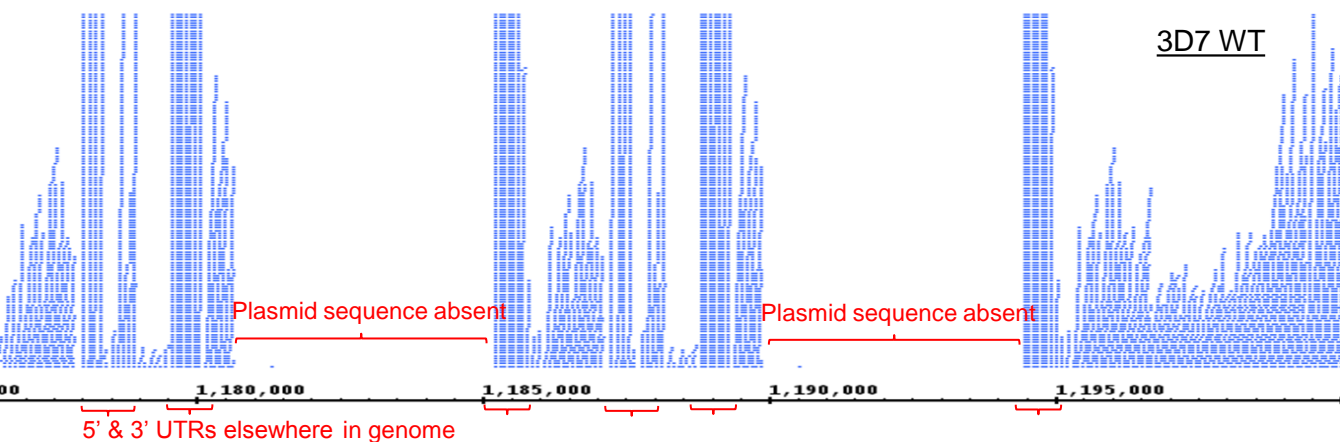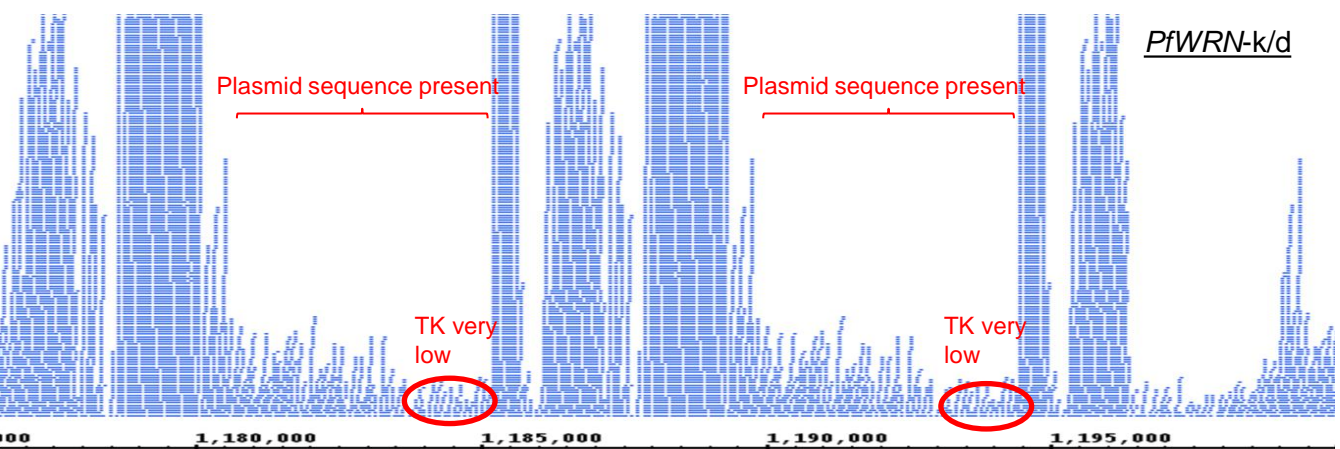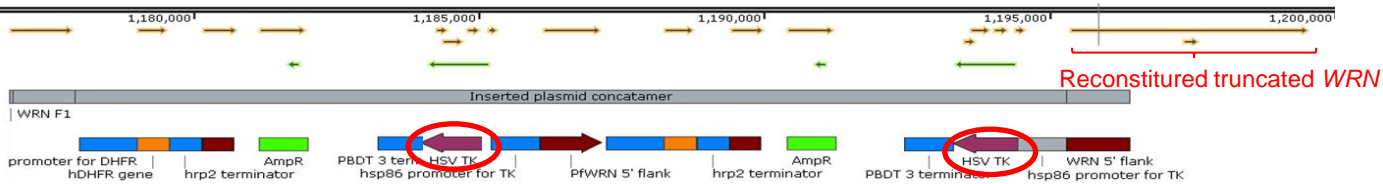

Disrupted *PfWRN* locus

Supplement: S2 Fig — (A) Disruption of the PfWRN gene. Southern blot analysis of untransfected 3D7 and PfWRN-targeted parasites. Genomic DNAs were digested with PmlI and SnaBI. The Southern blot was probed with the PfWRN 5’ flank used in the targeting plasmid. A schematic of the digestion pattern and probe location(s) is shown below, demonstrating how a two-plasmid concatamer has integrated within the WRN locus. Lane 1, PfWRN targeting plasmid (P); lane 2, untransfected wildtype 3D7 (3D7 WT); lanes 3 and 4, mixed populations of transfected parasites after selection with ganciclovir (PfWRN-k/d M) (the population in lane 4 has additionally been cycled off WR for one month); lanes 5–8,clonal PfWRN-k/d parasites after negative selection with ganciclovir (PfWRN-k/d clones). (B) Whole genome sequencing evidence for the nature of the locus in PfWRN-k/d. Upper panel: sequencing reads from PfWRN-k/d and 3D7 mapped to the 3D7 reference genome. A large increase of coverage can be seen at the 5’ and 3’ ends of PfWRN in the transfected line PfWRN-k/d. Lower panel: Two-plasmid concatamer sequence was inserted in the 3D7 reference genome and all reads were re-mapped on this modified reference genome. Each plasmid domain is labelled, with a “-2” to indicate the second copy of the plasmid. Reads from PfWRN-k/d now match this modified reference sequence. Note that reads mapping to human genes such as hDHFR were automatically discarded, as per Sanger Institute policy. (C) RNA-seq data showing disruption of PfWRN gene. As in S1B Fig, reads from PfWRN-k/d and 3D7 WT were aligned to the PfWRN locus, both in the native 3D7 genome (top) and in its disrupted form, provided as a modified reference genome (bottom). Each image displays stacks of aligned reads. In 3D7 WT parasites there is coverage across the whole of the PfWRN open reading frame (ORF) but no coverage of the plasmid sequences in the modified locus because they does not exist in the WT genome. By contrast, RNA from PfWRN-k/d parasites shows transcri [file pgen.1007490.s002.pdf]

Figure S3

A

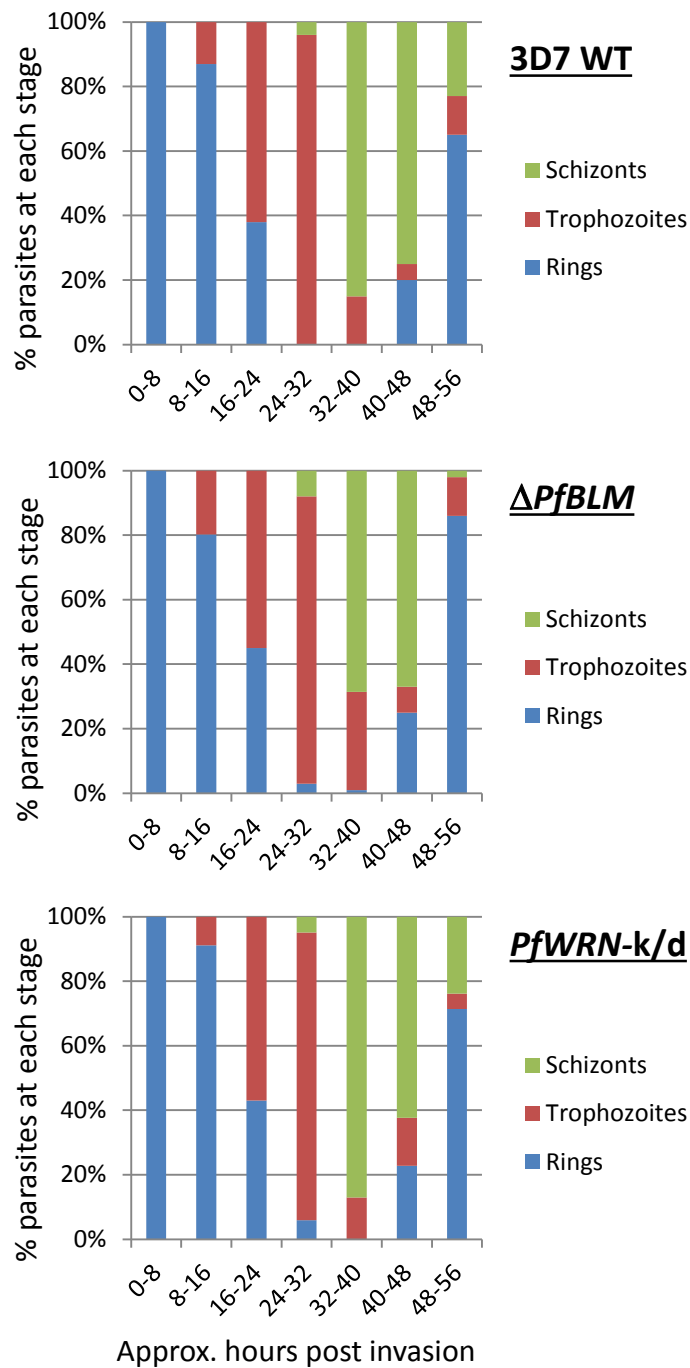

B

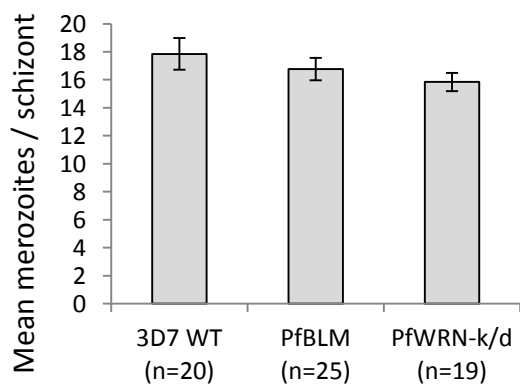

D

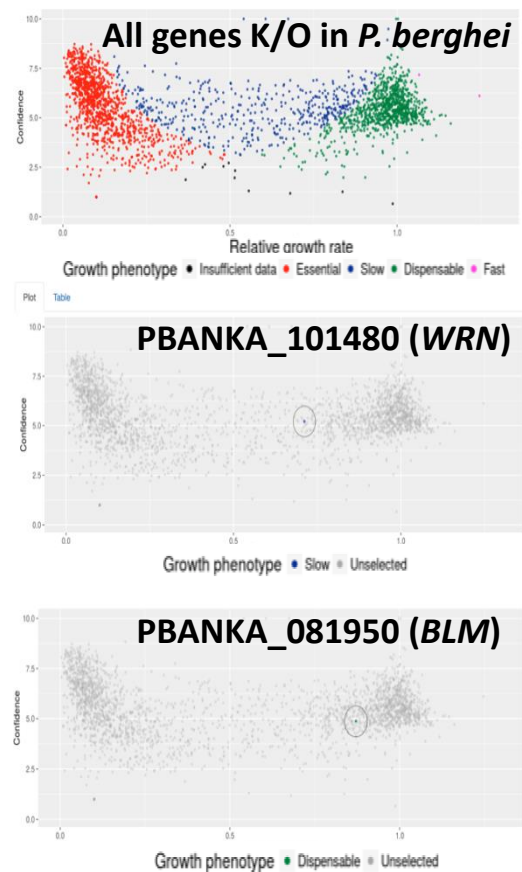

C

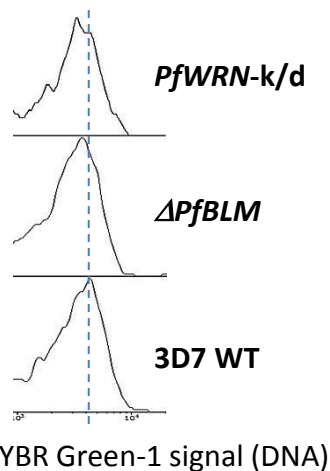

Supplement: S3 Fig — (A) Cell cycle of the ΔPfBLM, PfWRN-k/d and parent lines, assessed as previously described [72]–i.e. tight synchronisation of parasites at early rings and then staging of 100 parasites by morphology every 8h. No major or consistent change in the length of each morphological stage was observed: representative data shown from one of three biological replicates. (B) Numbers of merozoites per mature segmented schizont in the ΔPfBLM, PfWRN-k/d and parent lines. Approximately 20 schizonts were counted in each line. Differences did not attain statistical significance by one-tailed t-test, but merozoite numbers trended down in both mutants, approaching significance (p = 0.07) in PfWRN-k/d. (C) DNA content of mature schizonts in the ΔPfBLM, PfWRN-k/d and parent lines measured by SYBR Green 1 DNA staining and flow cytometry (5000 parasites per sample). Dotted line marks the peak of fluorescence in the parent line, for comparison with that in each mutant. (D) Data from the PlasmoGEM project (http://plasmogem.sanger.ac.uk/phenotypes) showing the growth phenotypes of P. berghei BLM and WRN knockouts. (PDF) [file pgen.1007490.s003.pdf]

Figure S4

A

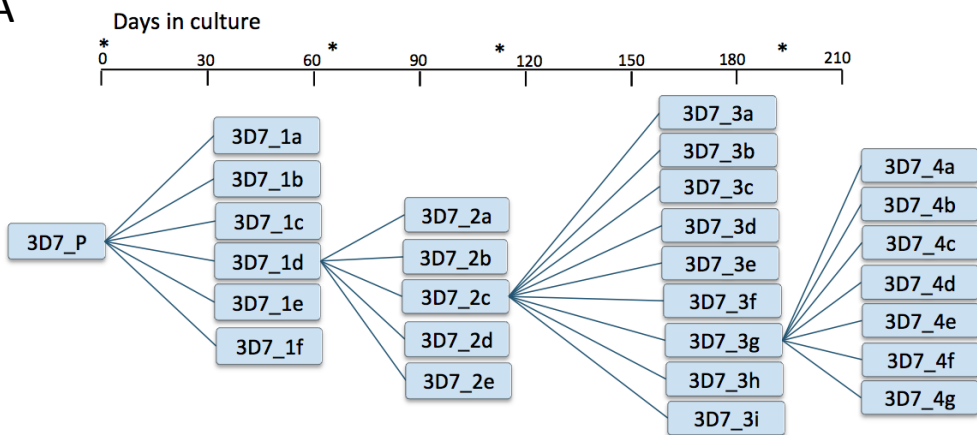

B

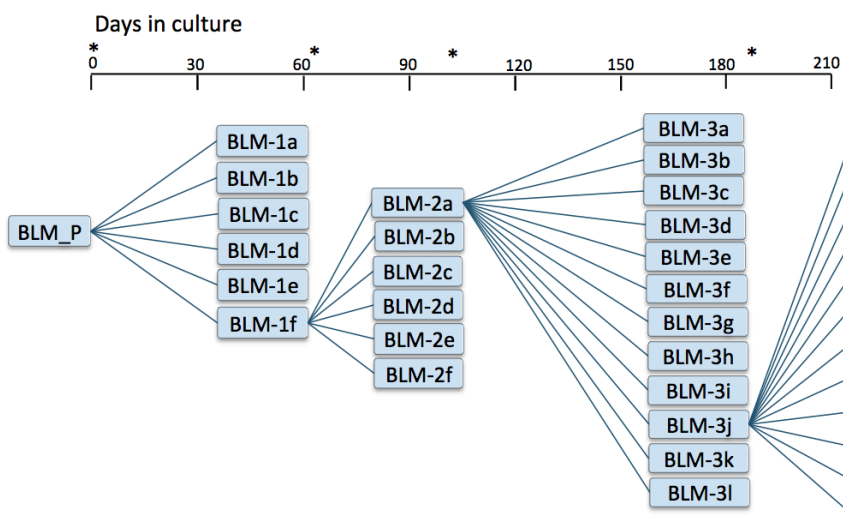

C

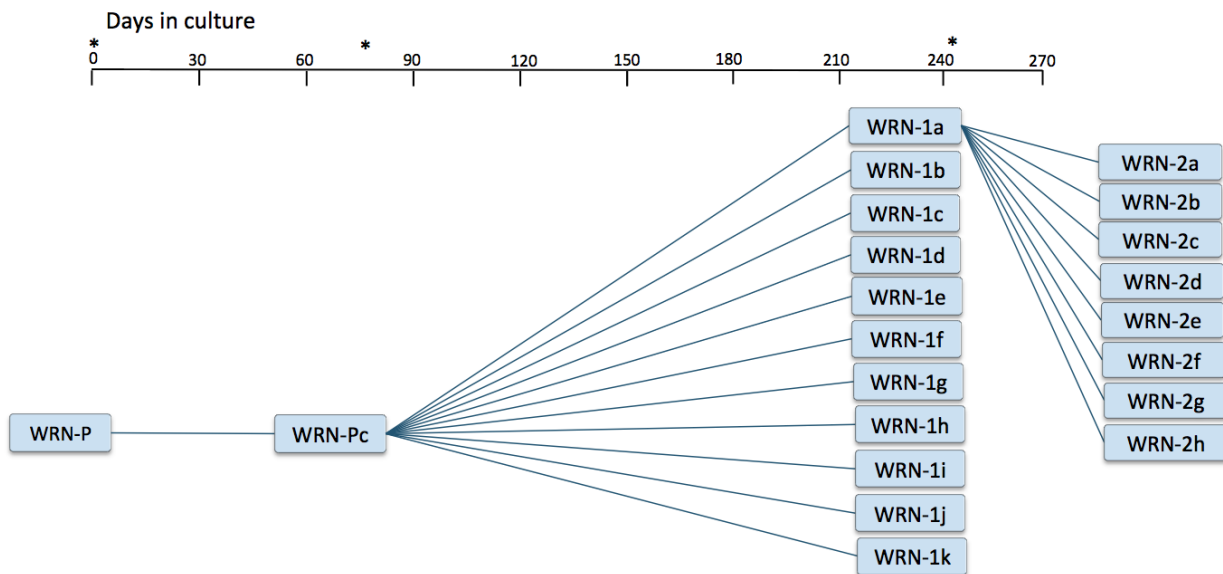

Supplement: S4 Fig — Clone trees were built by regularly subcloning individual parasites from the 3D7 wild-type (A), ΔPfBLM (B) and PfWRN-k/d (C) lines. Asterisks on the time line indicate the day of the cloning by limiting dilution. Note that the PfWRN-k/d parental line was cloned out before generating the first generation of subclones. By contrast, the first generation of 3D7 and ΔPfBLM subclones cannot be used to calculate mutation rates as the mutations identified within these genomes would have occurred before the start of the clone tree. (PDF) [file pgen.1007490.s004.pdf]

Figure S5

A

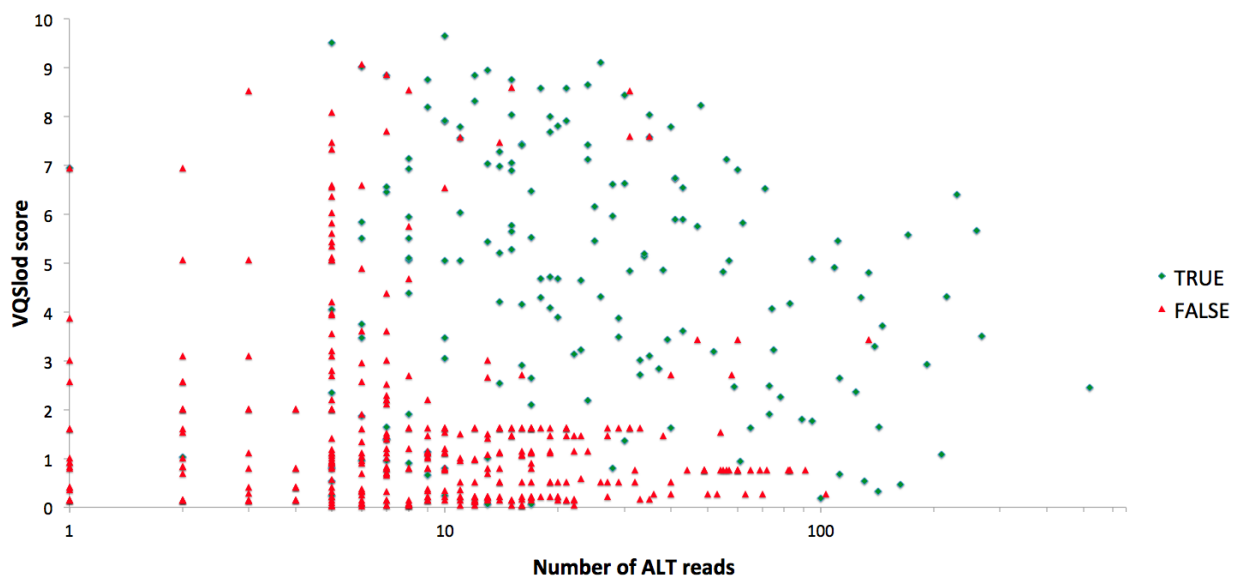

B

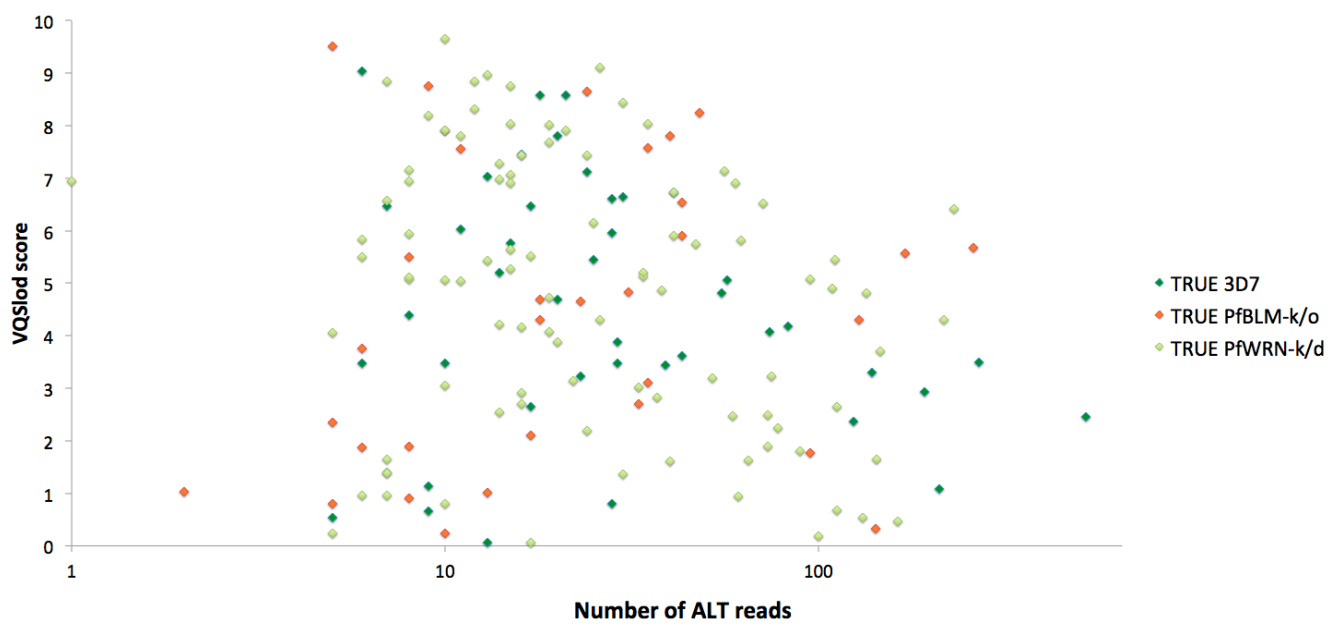

Supplement: S5 Fig — (A) VQSlod score (from GATK’s VQSR, y-axis) is plotted versus the number of reads indicating the putative micro-indel. If the read pileup visualised in Savant did not unequivocally show an indel in a progeny sample and its absence in its parent, it was labelled “False”, as described in [3]. As expected, hits with higher VQSlod score and number of Alt reads were more likely to be “True”. (B) Scatter plot as in (A), displaying only the “True” micro-indels hits, with no bias towards any clone tree. In both graphs the X-axis is on a log scale. (PDF) [file pgen.1007490.s005.pdf]

Figure S8

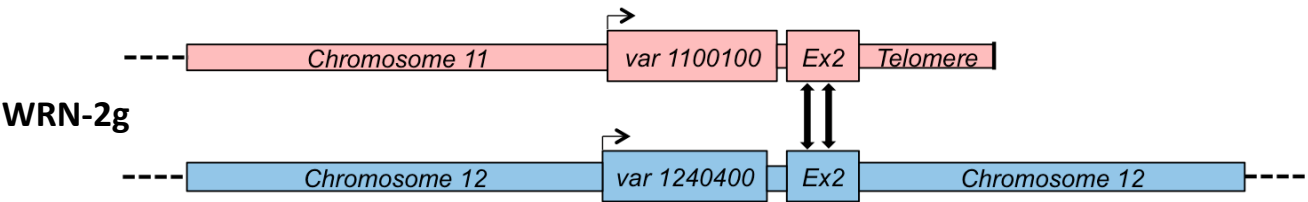

Supplement: S8 Fig — In subclone WRN-2g, we detected a double recombination between the exon 2s of subtelomeric group B var PF3D7_1100100 and internal group B var PF3D7_1240400. Note that an odd number of cross-over events would have led to a fusion between the two chromosomes, likely non-viable nor detectable. (PDF) [file pgen.1007490.s008.pdf]

Figure S9

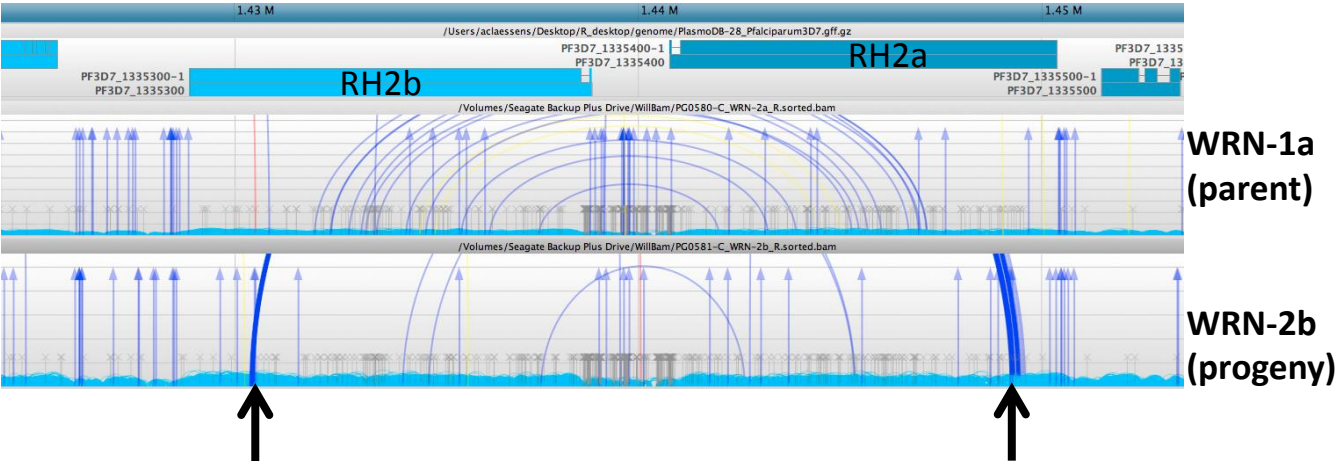

Supplement: S9 Fig — Rh2a and Rh2b (reticulocyte binding protein homologue) sequences show 91.8% identity and are located on opposite strands in head-to-head configuration, thus they are almost mirror-image of each other. They are both implicated in binding to the erythrocytes for invasion [73]. In subclone WRN-2b reads (designated by black arrows) indicate an inversion spanning 18kb centred halfway between the two genes. This unusual event might be reminiscent of how one gene was originally generated from the other. (PDF) [file pgen.1007490.s009.pdf]

Figure S10

A

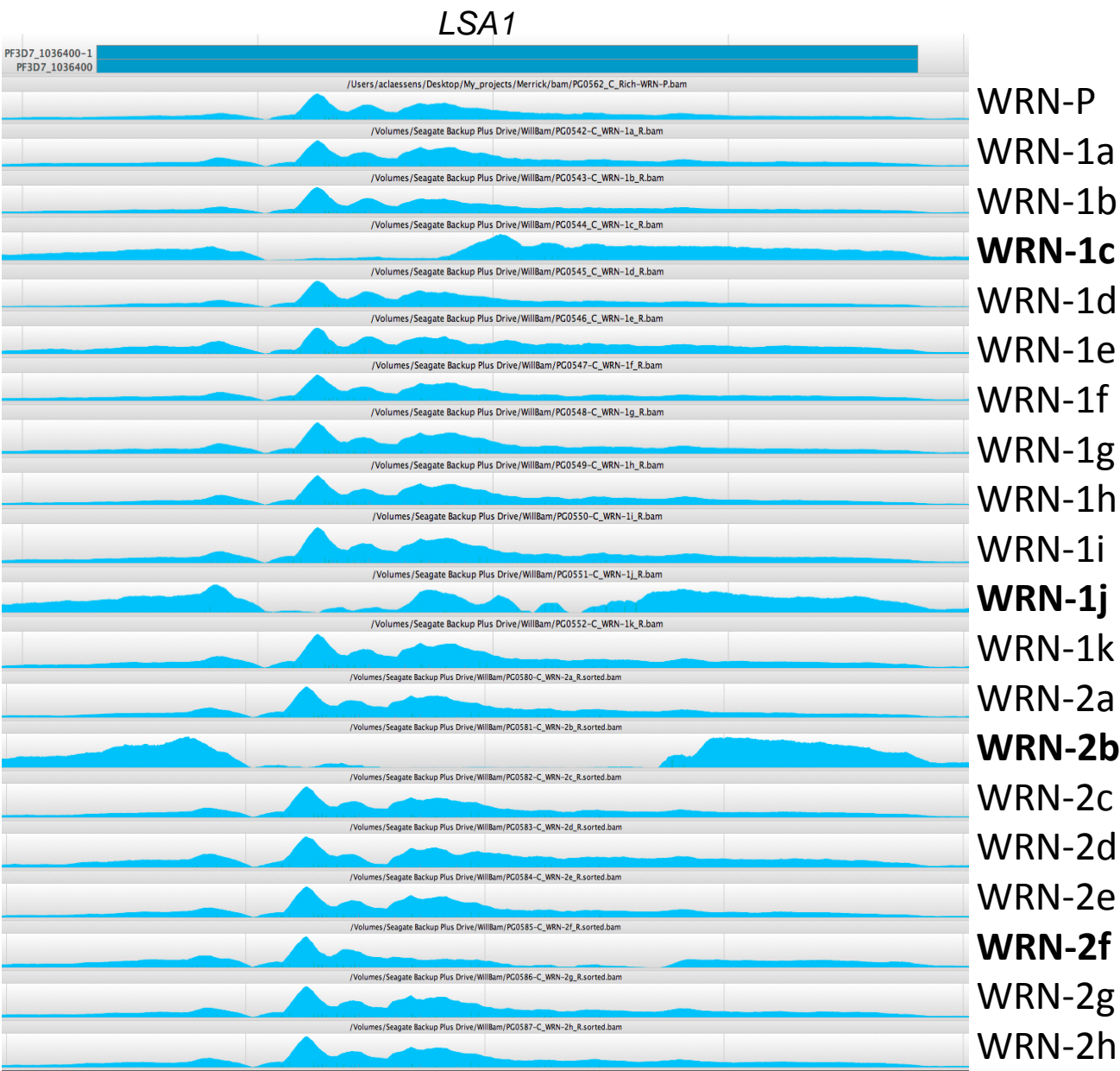

Figure S10 (cont).

B

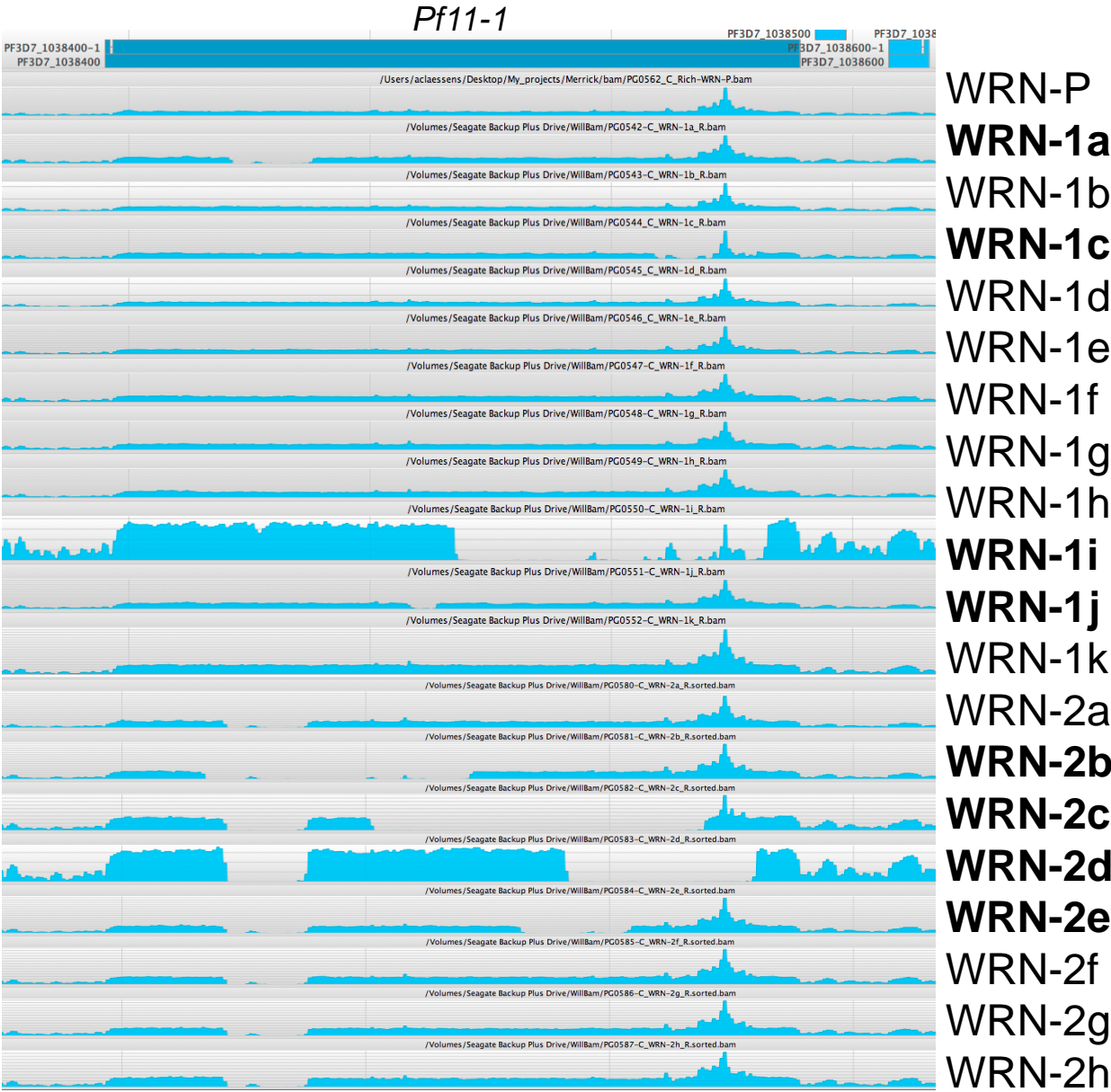

Supplement: S10 Fig — (A) Read coverage of the liver-stage antigen (LSA1, PF3D7_1036400). Four subclones with a mutation are highlighted in bold. (B) Read coverage over the Pf11-1 gene (PF3D7_1038400). Eight mutant subclones are highlighted. WRN-1a is the parent of the second generation, thus all progeny subclones inherited the Pf11-1 deletion present in WRN-1a as expected. (PDF) [file pgen.1007490.s010.pdf]

Figure S11

A

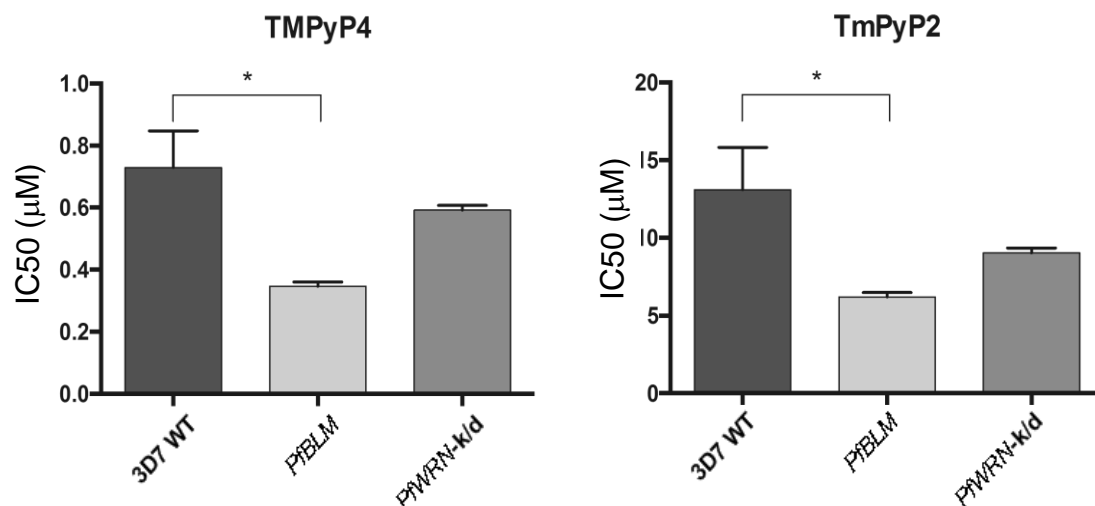

B

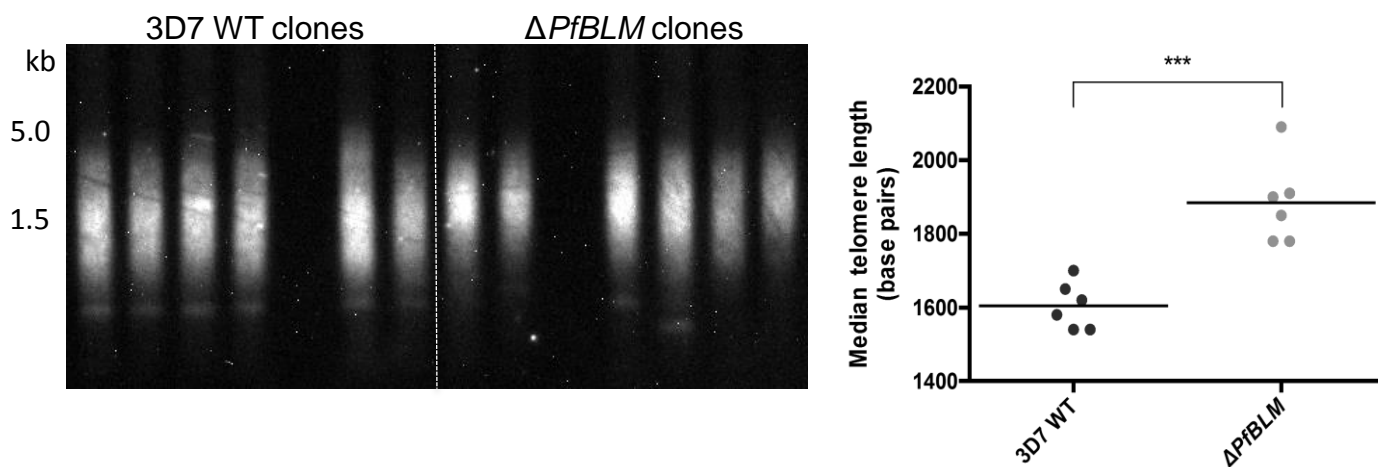

C

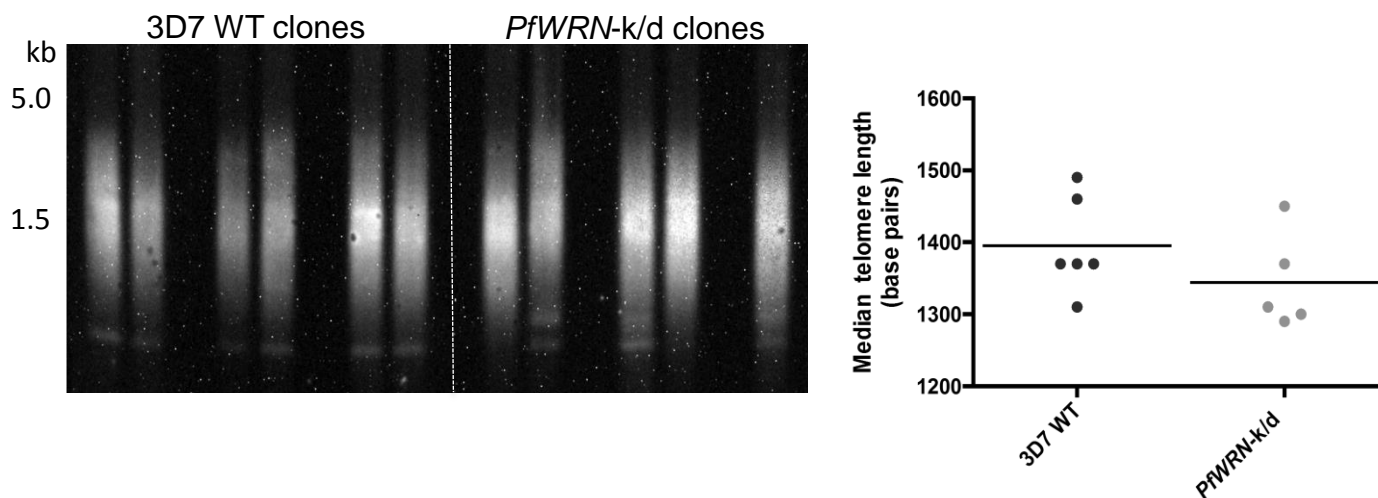

Supplement: S11 Fig — (A) Drug sensitivity of ΔPfBLM and PfWRN-k/d parasite lines to the G4-stabilising drugs TMPyP2 and TMPyP4. Mean IC50 values (μM) of TMPyP2 and TMPyP4 from 3 independent assays are shown. ΔPfBLM was significantly more sensitive to both TMPyP2 (p = 0.032) and TmPyP4 (p = 0.016). Error bars show standard error of the mean; statistical significance was determined using one-tailed t-tests (*, p<0.05). (B) Telomere restriction fragment (TRF) Southern blot showing telomere lengths in genomic DNA from 3D7 WT clones and ΔPfBLM clones. The empty lanes contained DNA ladder, which must be regularly interspersed between lanes to allow accurate measurement of the median DNA fragment size in adjacent telomere smears. The graph shows median telomere lengths calculated from this blot: ImageJ software was used to determine the median point of each smear. ΔPfBLM clones have significantly longer telomeres than wildtype 3D7 clones (p = 0.0001, two-tailed t-test). Data from one of two replicate TRF Southern blots are shown. (C) TRF Southern blot displaying telomere lengths from 3D7 WT and PfWRN-k/d clones. The graph shows median telomere lengths calculated from this blot using ImageJ software. Telomere length in PfWRN-k/d clones does not significantly differ from telomere length in 3D7 WT clones (p = 0.24, two-tailed t-test). Data shown are from one of two replicate TRF Southern blots. (PDF) [file pgen.1007490.s011.pdf]

Figure S12

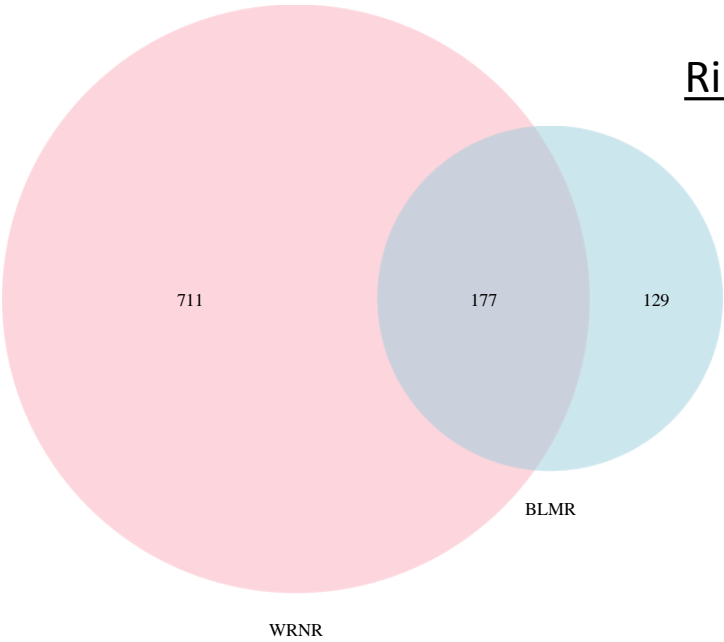

Ring stages: 21% overlap

Trophozoite stages:  
14% overlap

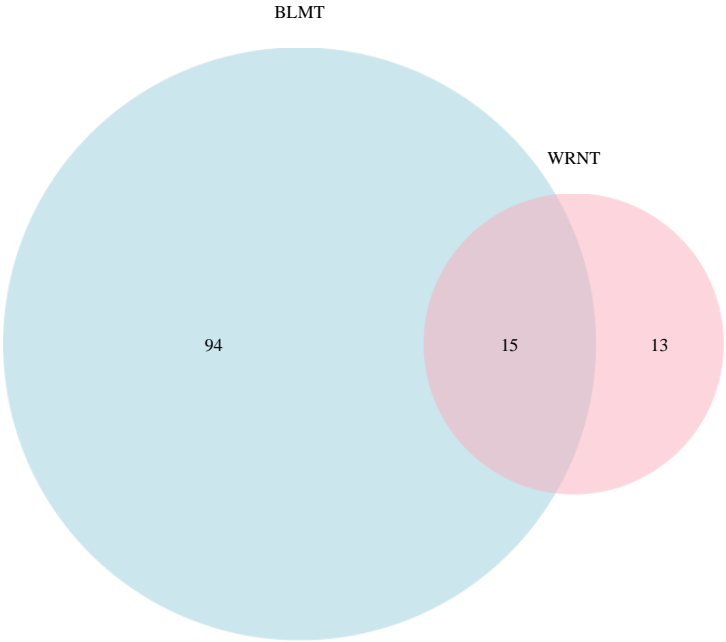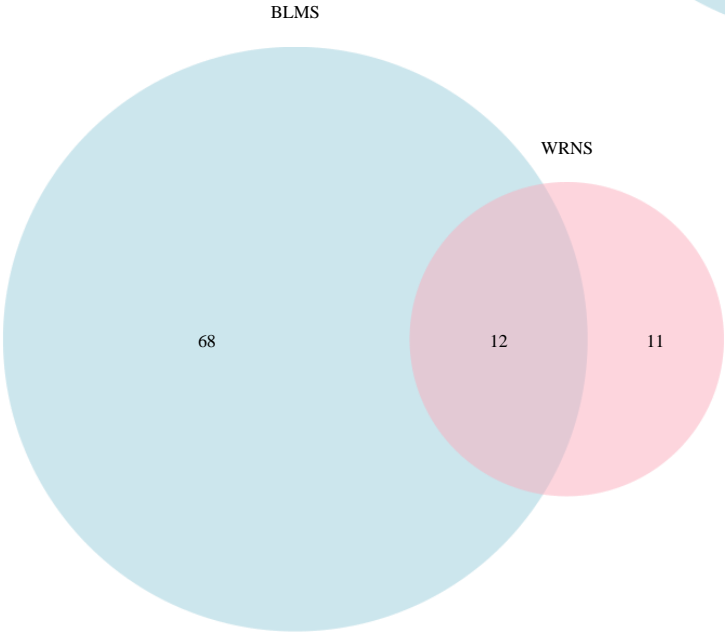

Schizont stages:  
15% overlap

Supplement: S12 Fig — Venn diagrams showing the numbers of genes differentially expressed in ΔPfBLM (blue circles) and PfWRN-k/d (pink circles) at each time point (R, rings; T, trophozoites; S, schizonts). Percentage overlap is calculated as the number of genes that are in common (deregulated in both ΔPfBLM and PfWRN-k/d) divided by the total number of deregulated genes in the two lines. (PDF) [file pgen.1007490.s012.pdf]

Figure S14

A

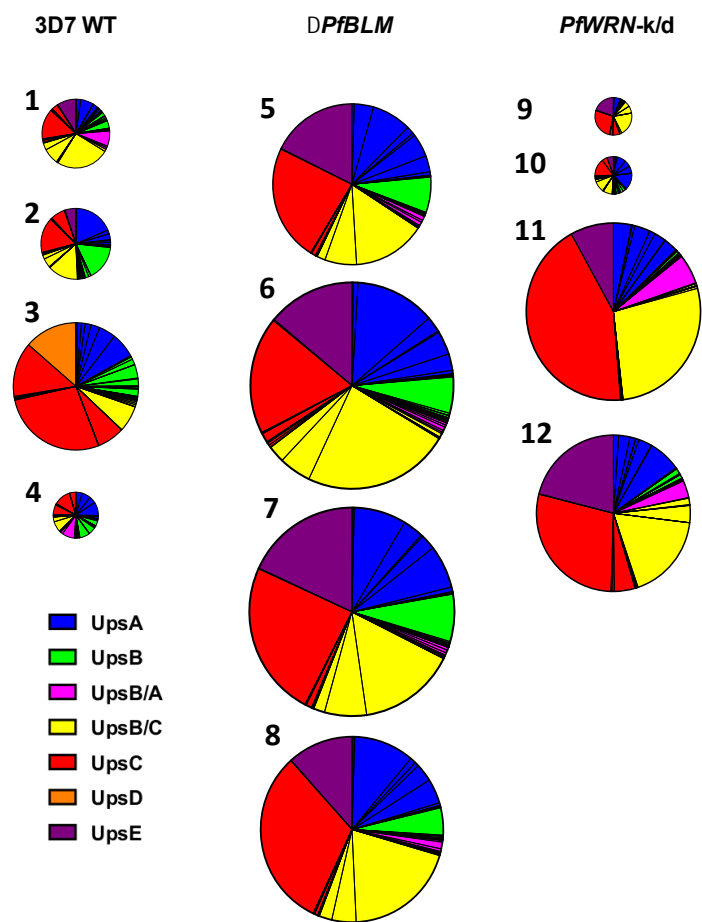

B

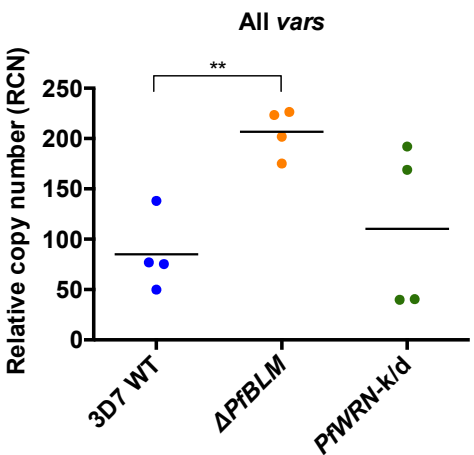

C

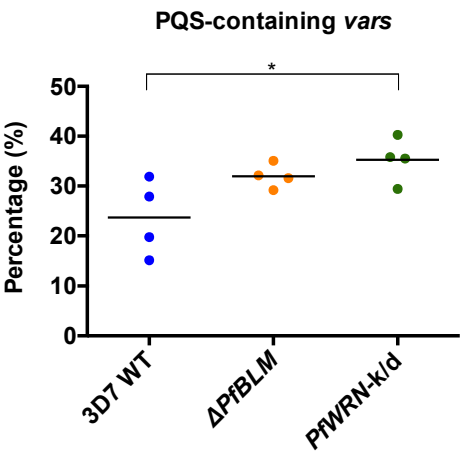

D

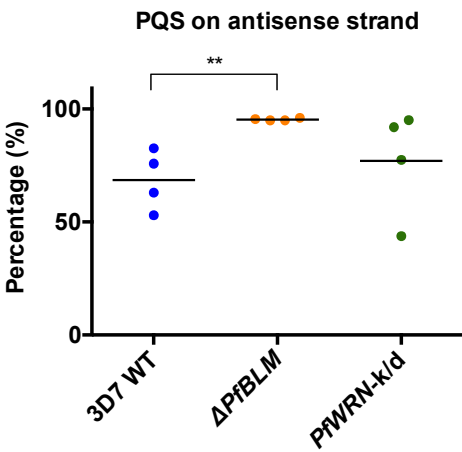

Supplement: S14 Fig — (A) Var gene expression patterns in four clones of each line: WT 3D7 (clones 1–4), ΔPfBLM (clones 5–8) and PfWRN-k/d (clones 9–12). Genes are grouped according to their upstream (ups) classification, A-E. Each segment of the pie chart shows the expression level of a single var gene and the total volume of the pie chart is proportional to the total level of var gene transcription. (B) Total level of var gene expression for WT 3D7, ΔPfBLM and PfWRN-k/d clones. Total transcript level is significantly higher in ΔPfBLM clones than in 3D7 WT clones (p = 0.0015, two-tailed t-test). (C) The level of var gene expression from PQS-containing var genes expressed as a percentage of the total var gene expression. This percentage is significantly higher in PfWRN-k/d clones when compared to 3D7 WT clones (p = 0.0392, two-tailed t-test). (D) Where var genes contain a PQS (in their coding or upstream regions), the PQS can occur on either the sense or antisense strand. The level of var gene expression from PQS-containing var genes which have a PQS on the antisense strand is expressed as a percentage of the total level of PQS-containing var gene expression. In ΔPfBLM clones compared to 3D7 WT clones a significantly higher proportion of PQS-containing var gene transcription occurs in genes where the PQS is on the antisense strand (p = 0.0068, two-tailed t-test). (PDF) [file pgen.1007490.s014.pdf]

Figure S16

A

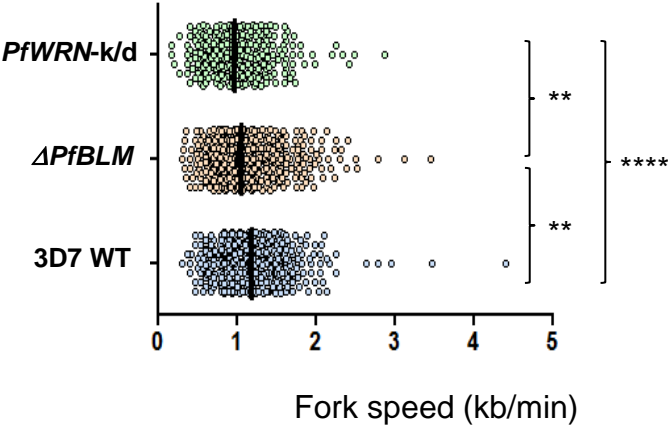

B

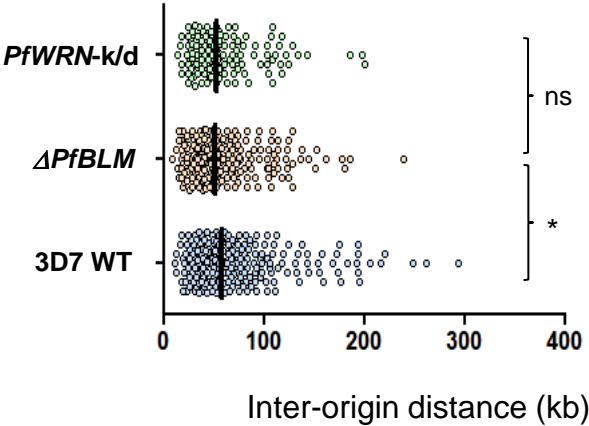

C

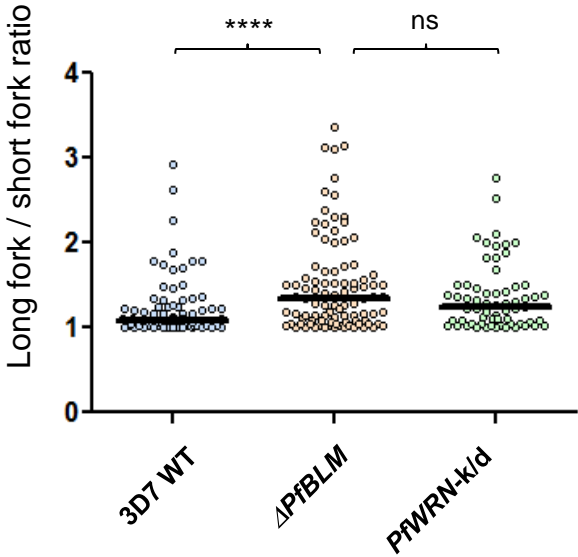

D

Percentage of forks (%)

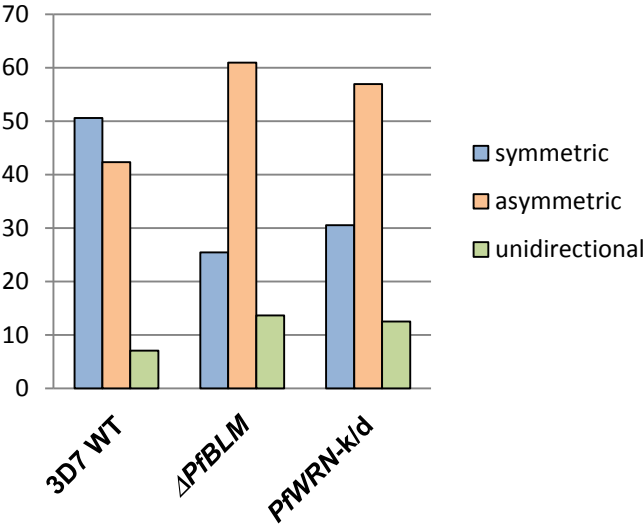

Supplement: S16 Fig — (A, B and C) Comparative analysis of replication fork speed (B) inter-origin distances (C) and asymmetric forks (long fork to short fork ratios) (D) from synchronous blood-stage parasites, in an experiment conducted separately from the experiment illustrated in Figs 4 and 5. Black bars on dot plots indicate median values. The two-tailed Mann-Whitney test was used to calculate the corresponding P values (ns, not statistically significant P value; * P < 0.05; ** P < 0.01; *** P < 0.001; **** P < 0.0001). (D) Percentage of symmetric, asymmetric and unidirectional replication forks counted in the wild type and RecQ helicase mutants. The groups were significantly different by Chi-square test (P = 0.00605). (PDF) [file pgen.1007490.s016.pdf]

Figure S17

A

First experiment

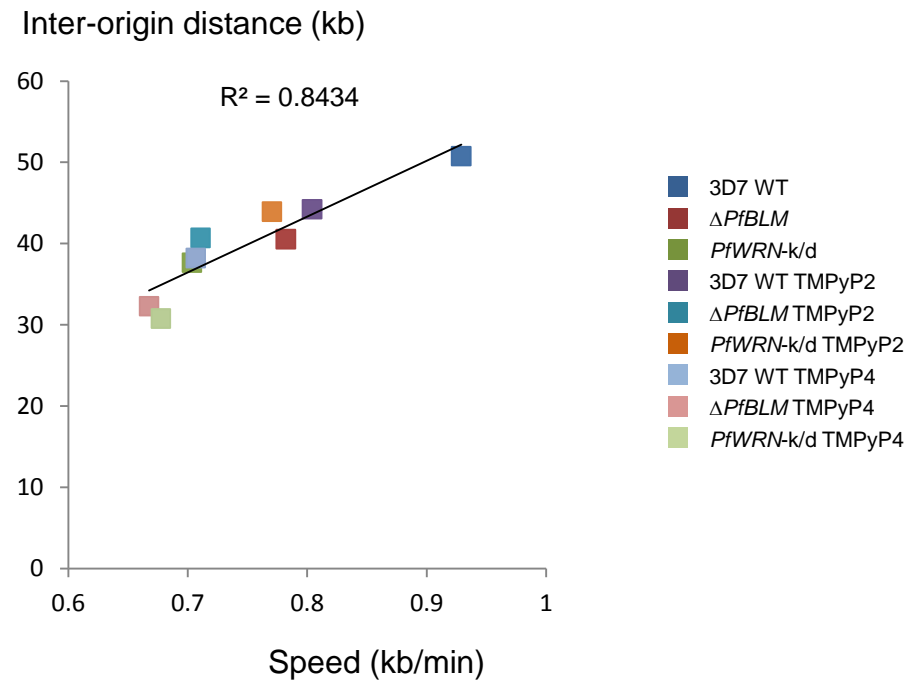

B

Second experiment

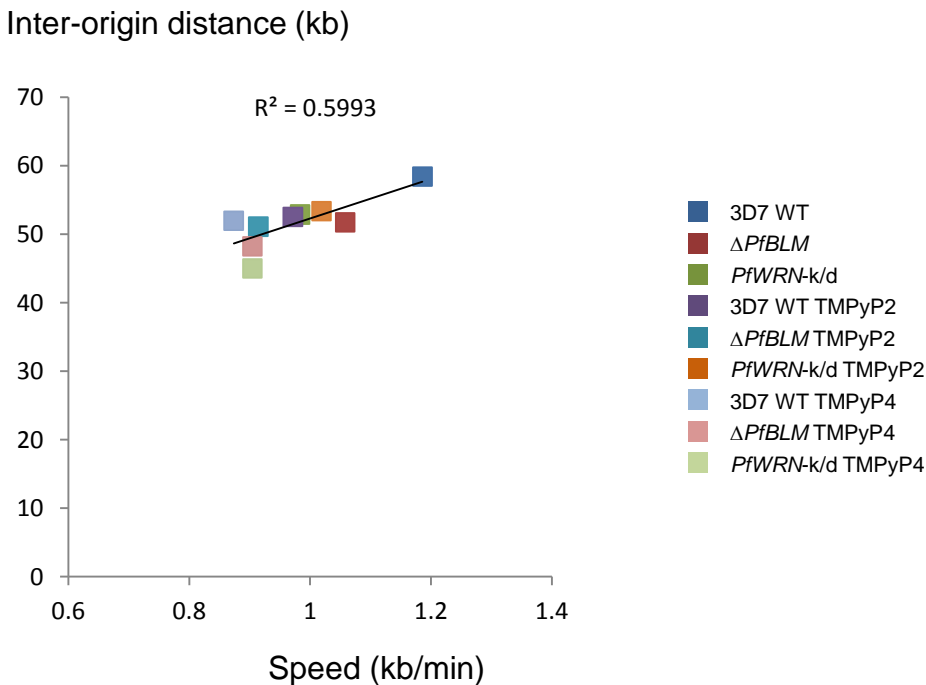

Supplement: S17 Fig — Positive correlation between median inter-origin distances (IODs) and median fork velocities in wild type and RecQ mutants, unchallenged and challenged with G4 stabilising drugs. Coefficient of determination (R2) is indicated. (A) First DNA combing experiment, shown in Figs 4 and 5 (B) Second DNA combing experiment, shown in S16 Fig. (PDF) [file pgen.1007490.s017.pdf]

Figure S18

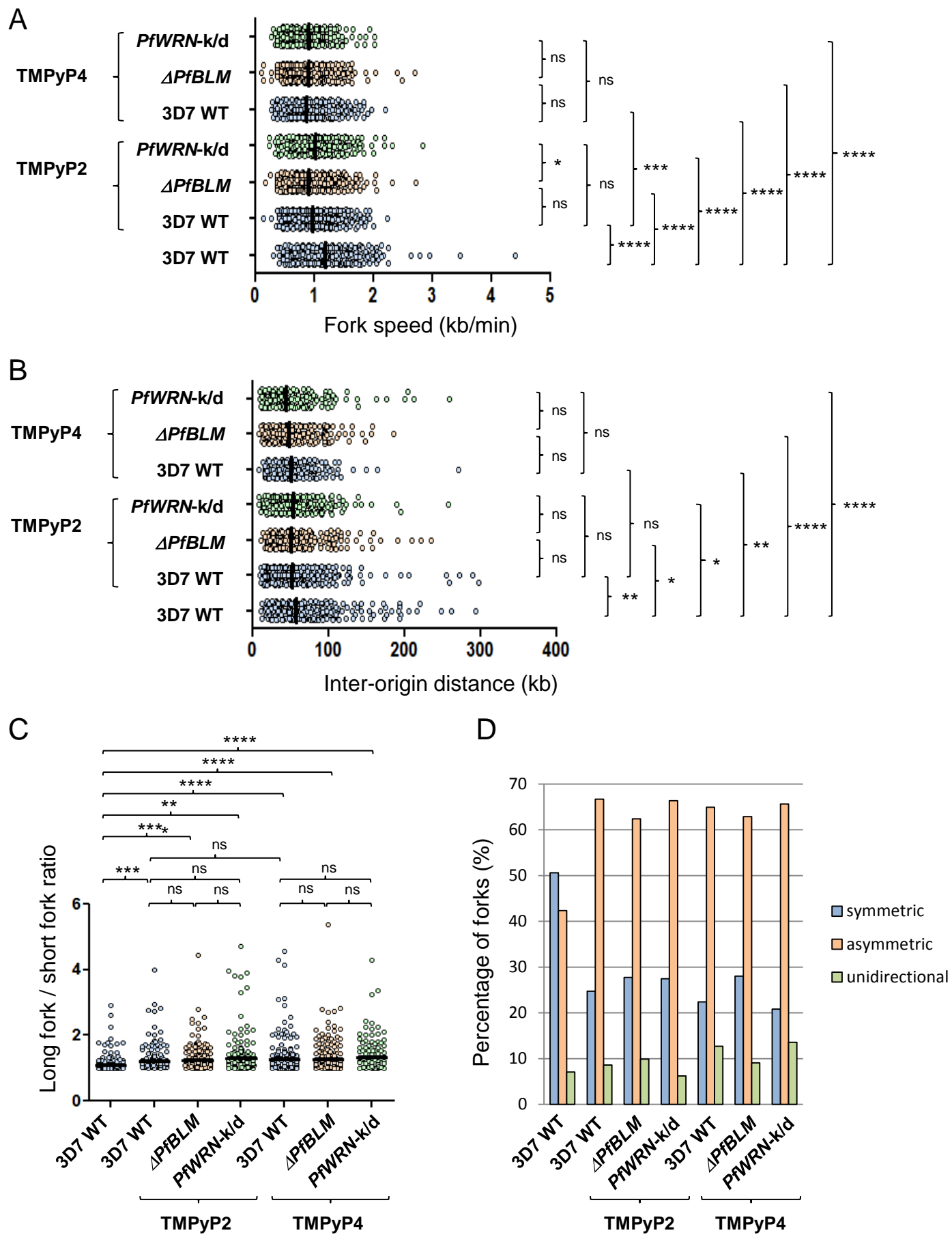

Supplement: S18 Fig — (A, B and C) Comparative analysis of replication fork speed (A) inter-origin distances (B) and asymmetric forks (long fork to short fork ratios) (C) from synchronous blood-stage parasites, in an experiment conducted separately from the experiment illustrated in Figs 4 and 5. Black bars on dot plots indicate median values. The two-tailed Mann-Whitney test was used to calculate the corresponding P values (ns, not statistically significant P value; * P < 0.05; ** P < 0.01; *** P < 0.001; **** P < 0.0001). (D) Percentage of symmetric, asymmetric and unidirectional replication forks counted in the wild type and RecQ helicase mutants challenged with G4 stabilising drugs. The groups were significantly different by Chi-square test (P = 0.0025). (PDF) [file pgen.1007490.s018.pdf]

Figure S19

3D7 R

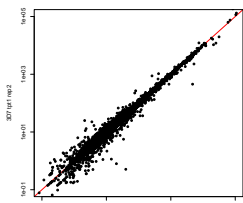

Replicate  
1 vz 2

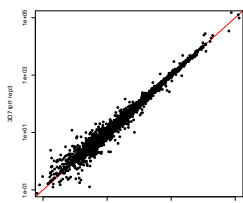

Replicate  
2 vz 3

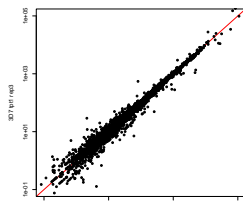

Replicate  
1 vz 3

3D7 T

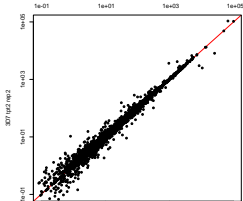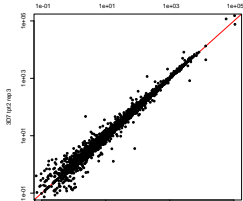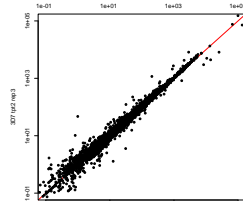

3D7 S

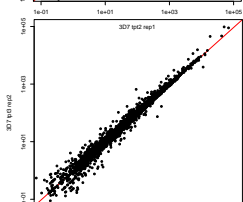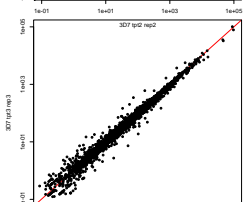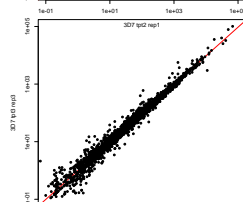

BLM R

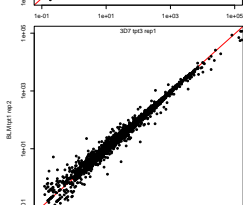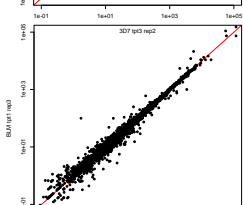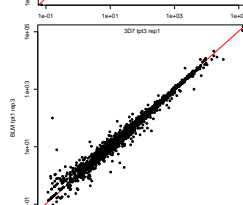

BLM T

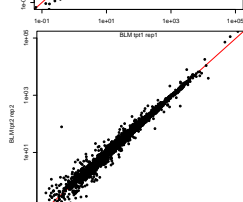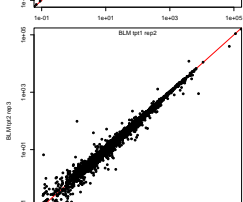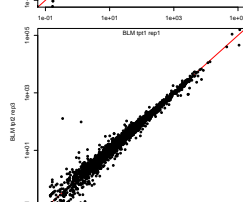

BLM S

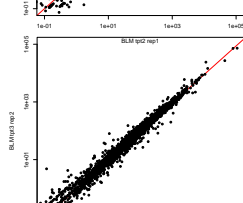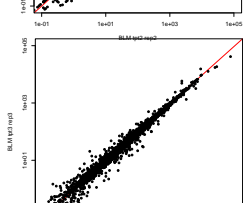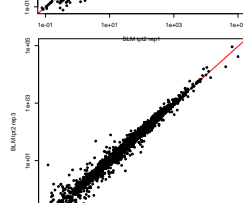

WRN R

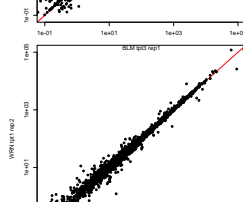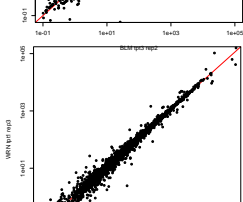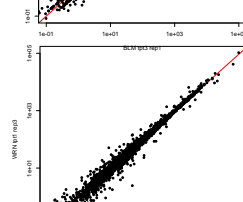

WRN T

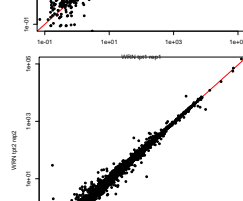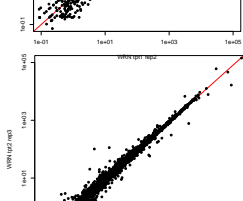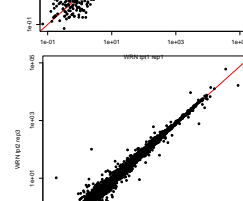

WRN S

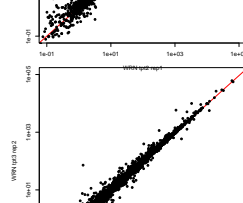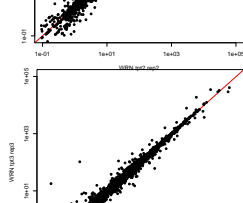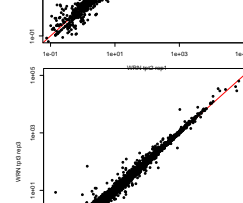

Supplement: S19 Fig — Scatter plots show the correlation in FPKM reads in each of three replicate RNA-seq experiments conducted at each of three timepoints (rings, R; trophozoites, T and schizonts, S) in the three lines 3D7 wildtype, ΔPfBLM and PfWRN-k/d. (PDF) [file pgen.1007490.s019.pdf]

Figure S20

A

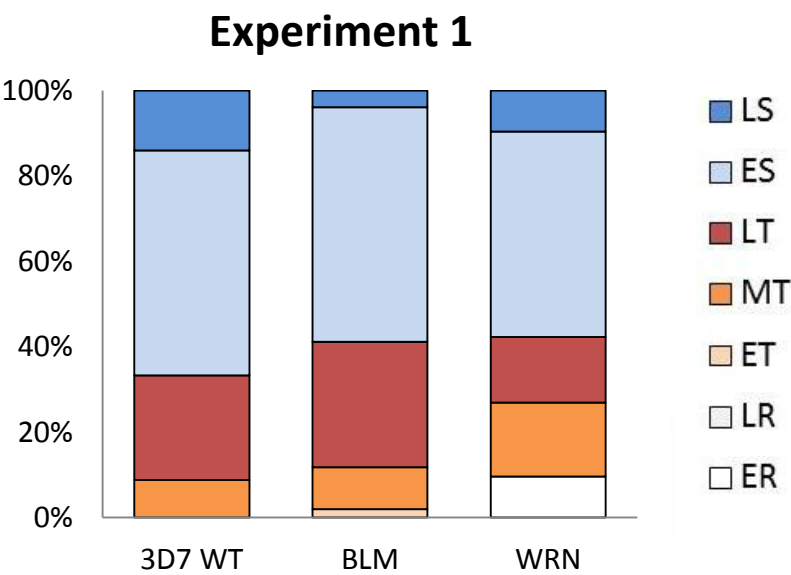

B

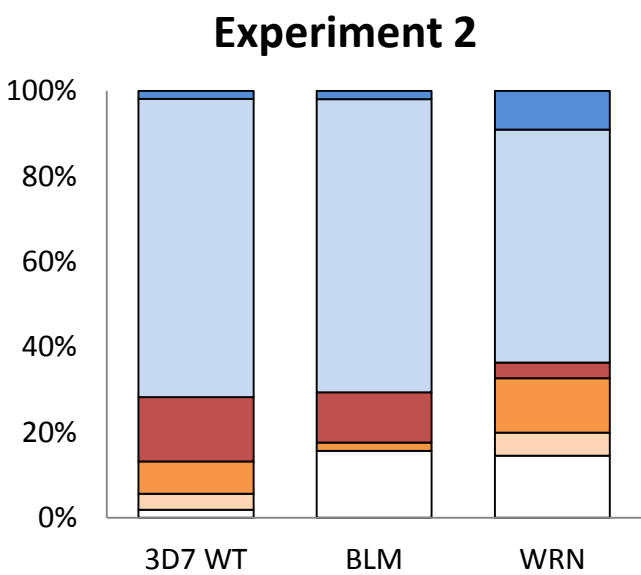

Supplement: S20 Fig — 50 parasites were counted from each line and classified by morphological stages, then the cultures were labelled and harvested in parallel for DNA combing. Staging is shown in experiment 1 (A) and experiment 2 (B). Parasites were classified as: ER small rings, LR large rings, ET early trophozoites (less than half the width of host cell), MT middle trophozoites (more than half the width of, but not entirely filling, host cell), LT late trophozoite (parasite filling all or nearly all of host cell), ES early schizont (discrete nuclear masses visible within parasite), LS late schizont (defined merozoites visible). Parasites were harvested when they were predominantly early schizonts with some late schizonts and late trophozoites. (PDF) [file pgen.1007490.s020.pdf]
