# Supplementary material for: RecQ helicases in the malaria parasite Plasmodium falciparum affect genome stability, gene expression patterns and DNA replication dynamics
Source: PLoS Genet. 2018 Jul 2;14(7):e1007490. doi: 10.1371/journal.pgen.1007490 (PMC6044543; doi:10.1371/journal.pgen.1007490)
Supplement: S6 Fig — (A, B, C) The types and chromosomal locations of micro-indels were similar in all clone trees. (D, E, F) The proportion of indel length divisible by 2 or 3 was comparable with previous clone trees [3]; however, there was a relatively high proportion of ‘indivisible by 3’ micro-indels (6 out of 12) in exons from the PfWRN-k/d line. These lead to frameshifts, but as expected they were found in non-essential genes. (G) As a convention, micro-indels are left-aligned in a repetitive region, i.e. the inserted / deleted sequence is upstream of the repeat. We analysed the 15bp downstream sequences into 3 categories: homorepeat ([A]n or [T]n), TA repeat ([TA]n) or ‘Complex’ for any other type of repeats. Homorepeats were proportionally 3.1 times more common in PfWRN-k/d compared to 3D7. (PDF) [file pgen.1007490.s006.pdf]

Figure S6

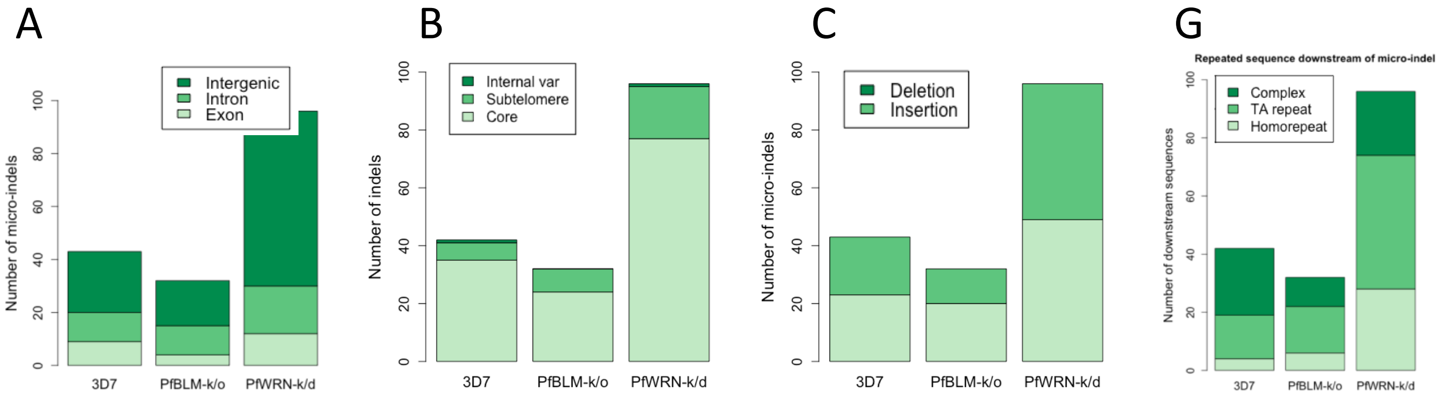

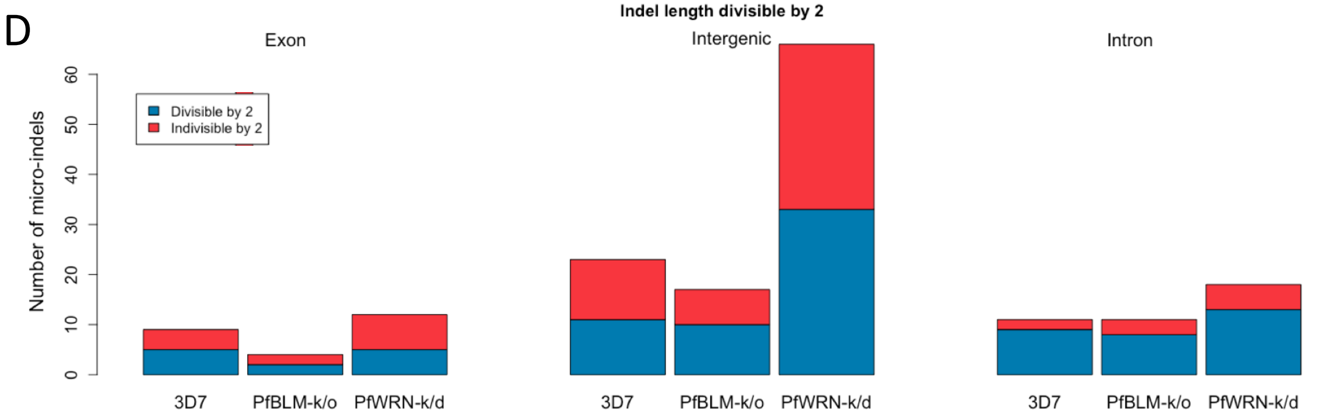

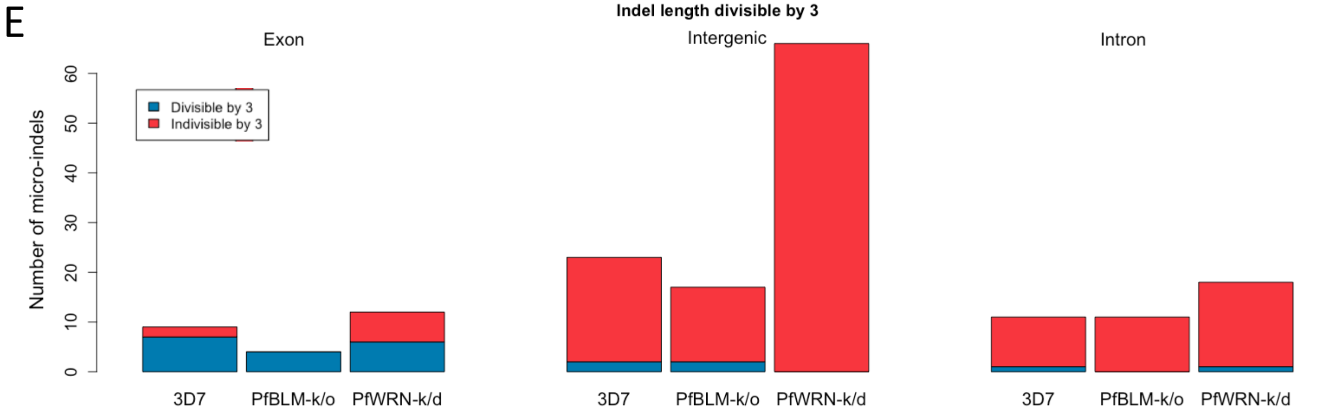

F

Strain	Progeny	Chr	POS	REF	ALT	Indel length	Variant	GeneID	Annotation
**3D7**	3D7-1c	05	1330704	TTTTA	TTTTATTTA	4	Insertion	PF3D7_0533000	Rifin pseudogene
**3D7**	3D7-1c	14	2429016	AC	A	-1	Deletion	PF3D7_1459200	WD repeat-containing protein, putative
**WRN**	WRN-1j	10	216986	TA	T	-1	Deletion	PF3D7_1004600	Conserved Plasmodium membrane protein unknown function
**WRN**	WRN-1j	08	1274902	GT	G	-1	Deletion	PF3D7_0829800	Unspecified product
**WRN**	WRN-1j	03	83468	TA	T	-1	Deletion	PF3D7_0301300	Epoxide hydrolase 1
**WRN**	WRN-2b, WRN-2d	04	672758	GT	G	-1	Deletion	PF3D7_0415200	Conserved Plasmodium protein unknown function
**WRN**	WRN-2g	04	170289	C	CAT	2	Insertion	PF3D7_0402800	Erythrocyte membrane protein 1 PfEMP1 pseudogene
**WRN**	WRN-2h	12	191947	C	CAT	2	Insertion	PF3D7_1203800	Unspecified product
