# Supplementary material for: RecQ helicases in the malaria parasite Plasmodium falciparum affect genome stability, gene expression patterns and DNA replication dynamics
Source: PLoS Genet. 2018 Jul 2;14(7):e1007490. doi: 10.1371/journal.pgen.1007490 (PMC6044543; doi:10.1371/journal.pgen.1007490)
Supplement: S7 Fig — (A) Example of a recombination between tail-to-tail group A and group B var genes identified in subclone WRN-2e. In the upper panel, the yellow arc is reads with one end mapping to PF3D7_0800100 and the other to PF3D7_0800200. The coverage is doubled over the same region. Thus in our model (lower panel) both parental var genes are still present, with an extra chimeric pseudogene sequence made from the 3’ ends of PF3D7_0800100 and PF3D7_0800200. (B) The conserved pseudogene var1csa (PF3D7_0533100), with an upstream A sequence, is located at the very end of chromosome 5. In subclone WRN-1g, translocation reads indicate a recombination with PF3D7_0400400, a group A var gene located near the start of chromosome 5. This resulted in a chimeric pseudogene 0400400-Var1. A rif and a group B var genes got deleted in the process. (PDF) [file pgen.1007490.s007.pdf]

Figure S7

A

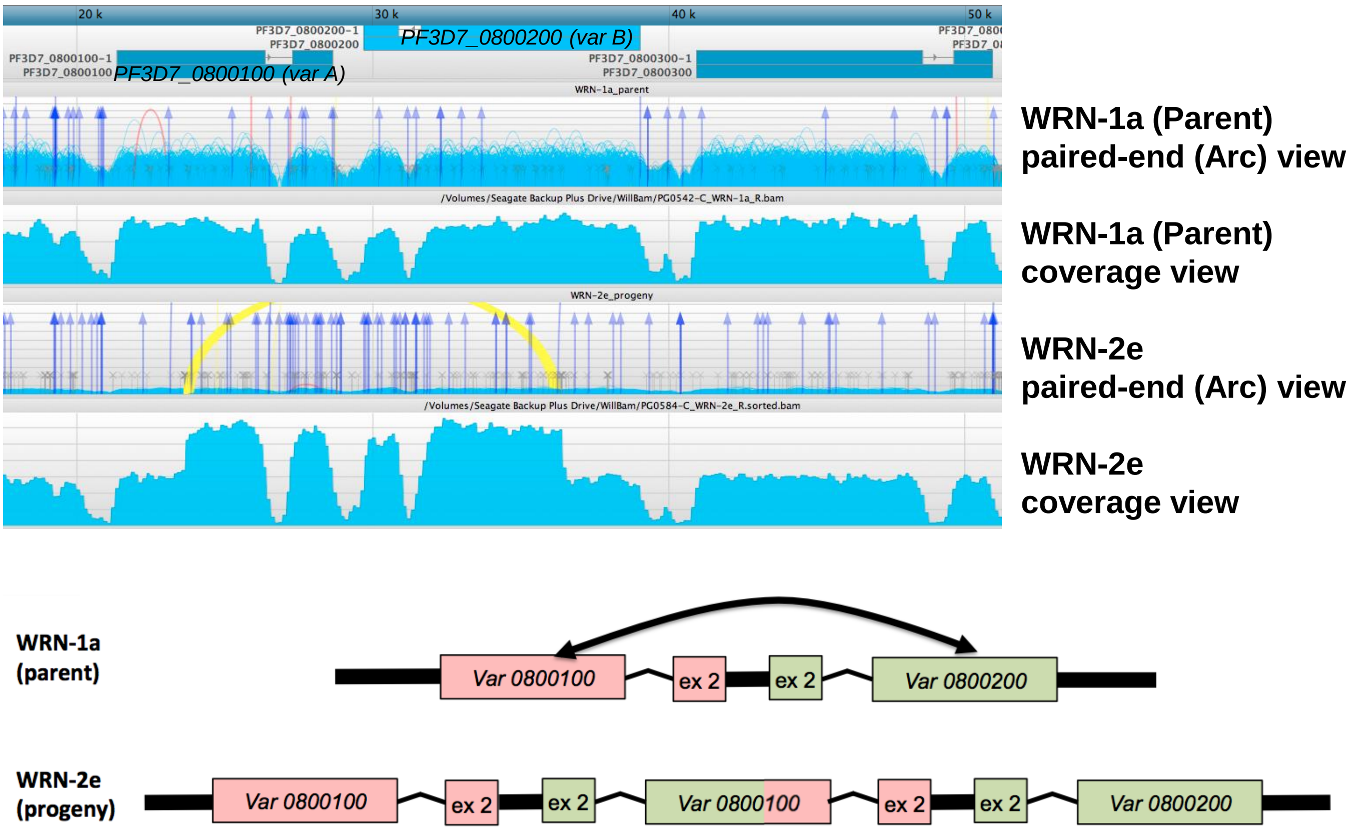
20 k
30 k
40 k
50 k
PF3D7_0800200-1
PF3D7_0800200
PF3D7_0800200 (var B)
PF3D7_0800100-1
PF3D7_0800100
PF3D7_0800100 (var A)
PF3D7_0800300-1
PF3D7_0800300
WRN-1a_parent
/Volumes/Seagate Backup Plus Drive/WillBam/PG0542-C_WRN-1a_R.bam
WRN-2e_progeny
/Volumes/Seagate Backup Plus Drive/WillBam/PG0584-C_WRN-2e_R.sorted.bam
WRN-1a (Parent) paired-end (Arc) view
WRN-1a (Parent) coverage view
WRN-2e paired-end (Arc) view
WRN-2e coverage view
WRN-1a (parent)
Var 0800100
ex 2
ex 2
Var 0800200
WRN-2e (progeny)
Var 0800100
ex 2
ex 2
Var 0800100
ex 2
ex 2
Var 0800200

B

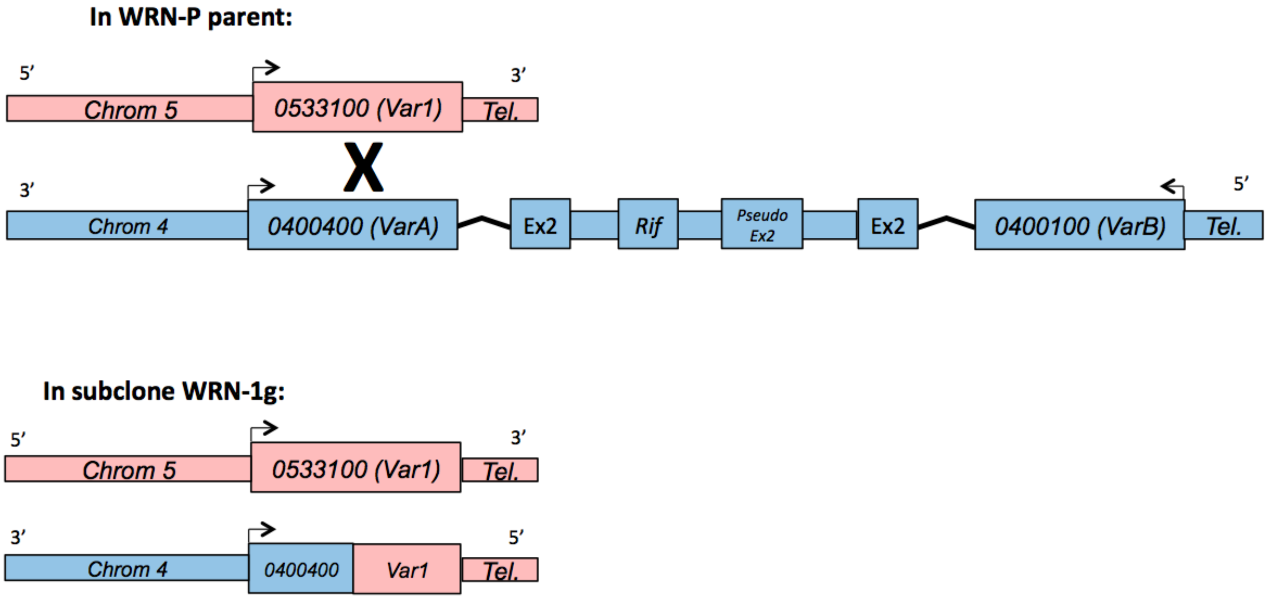
In WRN-P parent:
5′
3′
Chrom 5
0533100 (Var1)
Tel.
X
3′
5′
Chrom 4
0400400 (VarA)
Ex2
Rif
Pseudo Ex2
Ex2
0400100 (VarB)
Tel.
In subclone WRN-1g:
5′
3′
Chrom 5
0533100 (Var1)
Tel.
3′
5′
Chrom 4
0400400
Var1
Tel.
