# Supplementary material for: RecQ helicases in the malaria parasite Plasmodium falciparum affect genome stability, gene expression patterns and DNA replication dynamics
Source: PLoS Genet. 2018 Jul 2;14(7):e1007490. doi: 10.1371/journal.pgen.1007490 (PMC6044543; doi:10.1371/journal.pgen.1007490)
Supplement: S13 Fig — (A) Box-plot shows the percentage tandem-repeat (TR) content of all genes in the genome, versus that of those genes expressed at 80–100% maximum levels in rings (R, n = 1447), trophozoites (T, n = 1325) and schizonts (S, n = 1498). These gene sets were derived from transcriptomic data from [74], available in PlasmoDB [75]. Timepoints within this dataset were chosen to match the time-windows harvested for RNA-seq in RecQ mutants: 8-16h hpi (R), 24-30h (T) and 40-48h (S). Lines indicate medians, box and whiskers indicate interquartile and full ranges. (B) Table shows mean TR content for each gene set: there was no significant difference between the R, T, and S datasets as assessed by ANOVA. Interestingly, the most highly-expressed genes at all three stages have a higher TR content than the average for all genes in the genome (statistically significant differences as tested by 2-tailed T-test). However, all three differences are only about half as large as the difference between all-genes and the gene sets upregulated in ring stages of RecQ mutant lines (shown for comparison). The table also shows the TR content of var genes, grouped into all vars, and vars containing a PQS on the sense or antisense strand: in no group is the TR content different from that of all ring-stage genes. (C) Box-plot as in (A), showing the percentage low-complexity-region (LCR) content of the same gene sets. (D) Table shows mean LCR content for each gene set: there was no significant difference between the R, T, and S datasets as assessed by ANOVA. The most highly-expressed genes at all stages have a lower LCR content than the average for all genes in the genome. The table also shows the LCR content of var genes, which is lower than that of all ring-stage genes. (PDF) [file pgen.1007490.s013.pdf]

Figure S13

A

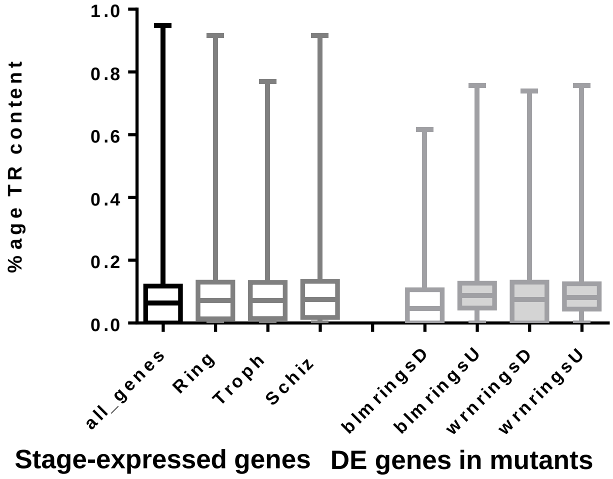

C

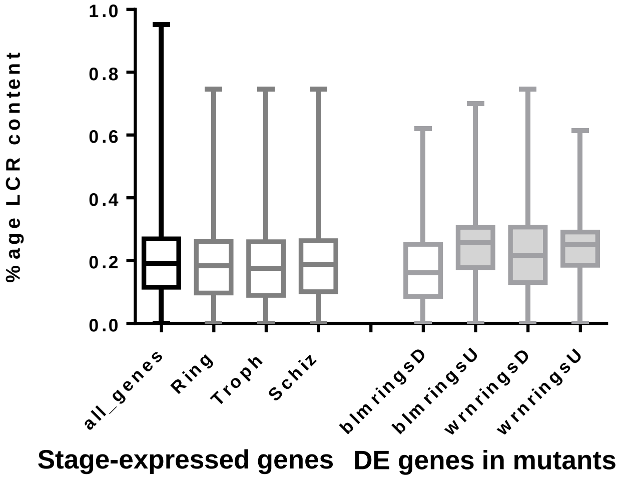

B

Gene set			Tandem Repeats (TRs)		
		Number	Mean TR content (%)	Significant difference from all genes?	Significant difference between stages
All genes		5602	7.8		
	80-100% in Rings	1447	9.1	**Y (p = 0.001)**	N - ANOVA p = 0.72
	80-100% in Trophs	1325	8.8	**Y (p = 0.001)**	
	80-100% in Schiz	1498	9.1	**Y (p = 0.001)**	
ΔBLM	Rings Down	159	7.0	N (p = 0.24)	
	Rings Up	146	10.1	**Y (p = 0.001)**	
WRN-k/d	Rings Down	702	8.6	**Y (p = 0.012)**	
	Rings Up	185	9.7	**Y (p = 0.002)**	
Vars	all vars	62	8.6	N (p = 0.37)	
	PQS sense	16	10.4	N (p = 0.08)	
	PQS antisense	13	8.5	N (p = 0.56)	

D

Gene set			Low complexity regions (LCRs)		
		Number	Mean LCR content (%)	Significant difference from all genes?	Significant difference between stages
All genes		5602	19.5		
	80-100% in Rings	1447	18.6	**Y (p = 0.006)**	N - ANOVA p = 0.25
	80-100% in Trophs	1325	18.1	**Y (p < 0.0001)**	
	80-100% in Schiz	1498	18.8	**Y (p = 0.041)**	
ΔBLM	Rings Down	18.1	16.2	N (p = 0.14)	
	Rings Up	**25.6**	**25.7**	**Y (p < 0.0001)**	
WRN-k/d	Rings Down	**22**	**21.8**	**Y (p < 0.0001)**	
	Rings Up	**24.7**	**25.1**	**Y (p < 0.0001)**	
Vars	all vars	62	14.1	**Y (p < 0.0001)**	
	PQS sense	16	15.2	**Y (p < 0.0001)**	
	PQS antisens	13	14.0	**Y (p = 0.002)**	
