# Supplementary material for: RecQ helicases in the malaria parasite Plasmodium falciparum affect genome stability, gene expression patterns and DNA replication dynamics
Source: PLoS Genet. 2018 Jul 2;14(7):e1007490. doi: 10.1371/journal.pgen.1007490 (PMC6044543; doi:10.1371/journal.pgen.1007490)
Supplement: S15 Fig — Graphs showing the relative copy number (RCN) of each individual var gene in four WT 3D7 (A), ΔPfBLM (B) and PfWRN-k/d (C) clones. Genes are grouped according to their upstream (ups) classification, A-E. The numbers in the graph titles (1–12) correspond to the numbers of the pie charts displayed in S14 Fig. (D) Quantification of the variation in var expression patterns between the 4 clones of each line shown in (A-C). (PDF) [file pgen.1007490.s015.pdf]

Figure S15A

A

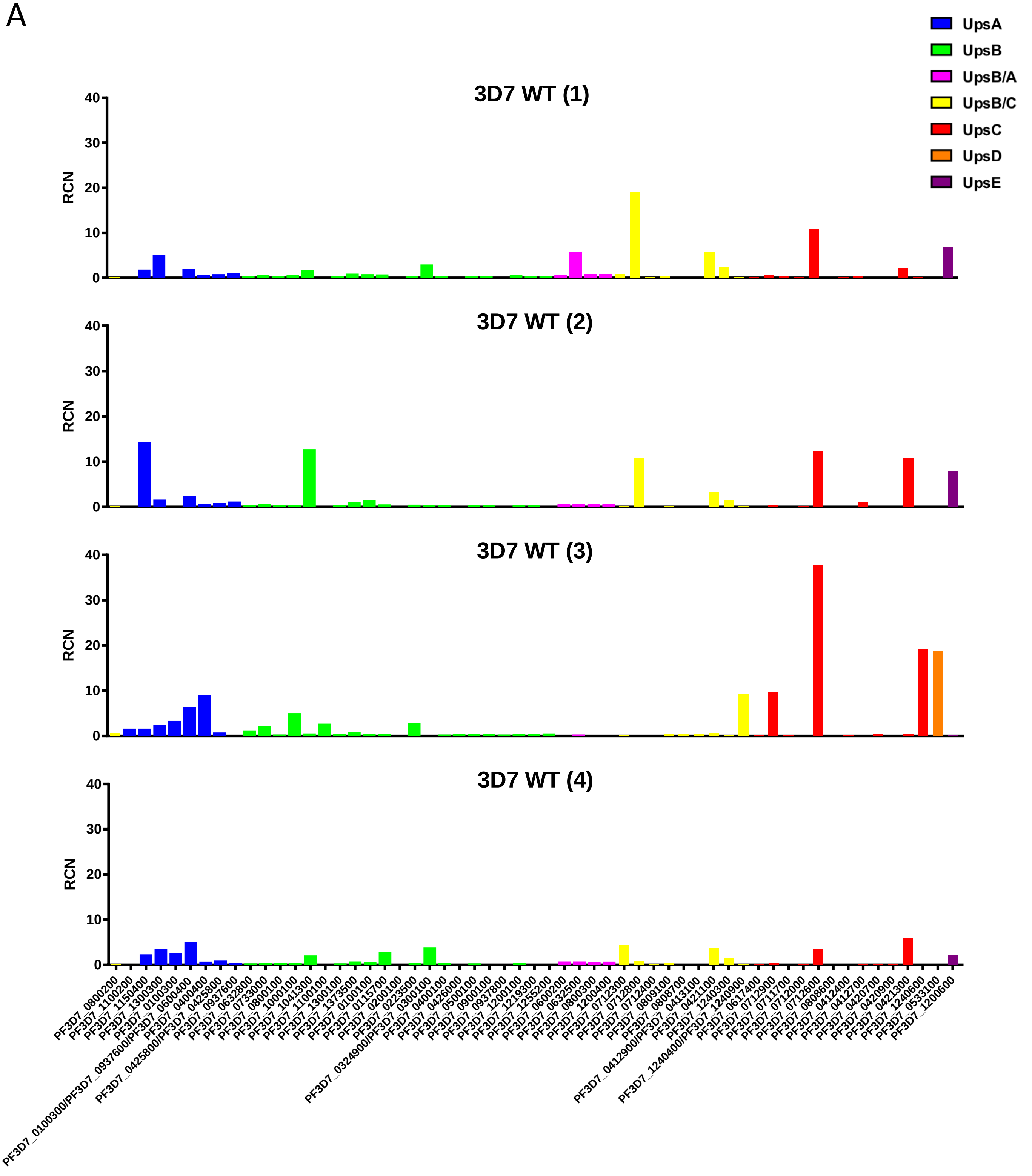

Figure S15B

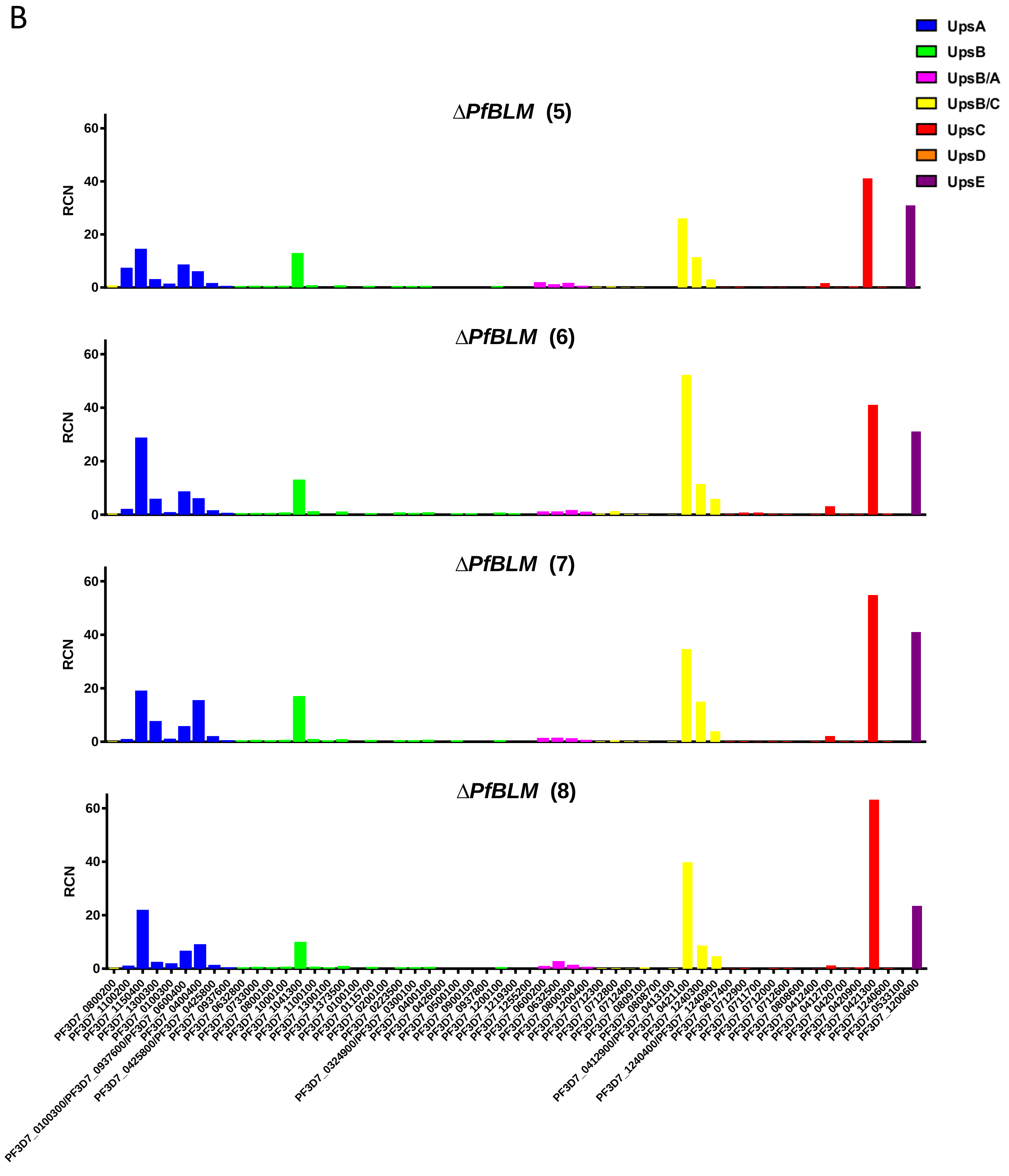
B
UpsA
UpsB
UpsB/A
UpsB/C
UpsC
UpsD
UpsE
ΔPfBLM (5)
ΔPfBLM (6)
ΔPfBLM (7)
ΔPfBLM (8)
RCN
0
20
40
60
PF3D7_0800200
PF3D7_1100200
PF3D7_1150400
PF3D7_1300300
PF3D7_0100300
PF3D7_0100300/PF3D7_0937600/PF3D7_0600400
PF3D7_0400400
PF3D7_0425800/PF3D7_0425800
PF3D7_0937600
PF3D7_0632800
PF3D7_0733000
PF3D7_0800100
PF3D7_1000100
PF3D7_1041300
PF3D7_1100100
PF3D7_1300100
PF3D7_1373500
PF3D7_0100100
PF3D7_0115700
PF3D7_0200100
PF3D7_0324900/PF3D7_0223500
PF3D7_0300100
PF3D7_0400100
PF3D7_0426000
PF3D7_0500100
PF3D7_0900100
PF3D7_0937800
PF3D7_1200100
PF3D7_1219300
PF3D7_1255200
PF3D7_0600200
PF3D7_0632500
PF3D7_0800300
PF3D7_1200400
PF3D7_0712300
PF3D7_0712800
PF3D7_0712400
PF3D7_0809100
PF3D7_0412900/PF3D7_0808700
PF3D7_0413100
PF3D7_0421100
PF3D7_1240400/PF3D7_1240300
PF3D7_1240900
PF3D7_0617400
PF3D7_0712900
PF3D7_0711700
PF3D7_0712000
PF3D7_0712600
PF3D7_0808600
PF3D7_0412400
PF3D7_0412700
PF3D7_0420700
PF3D7_0420900
PF3D7_0421300
PF3D7_1240600
PF3D7_0533100
PF3D7_1200600

Figure S15 C, D

C

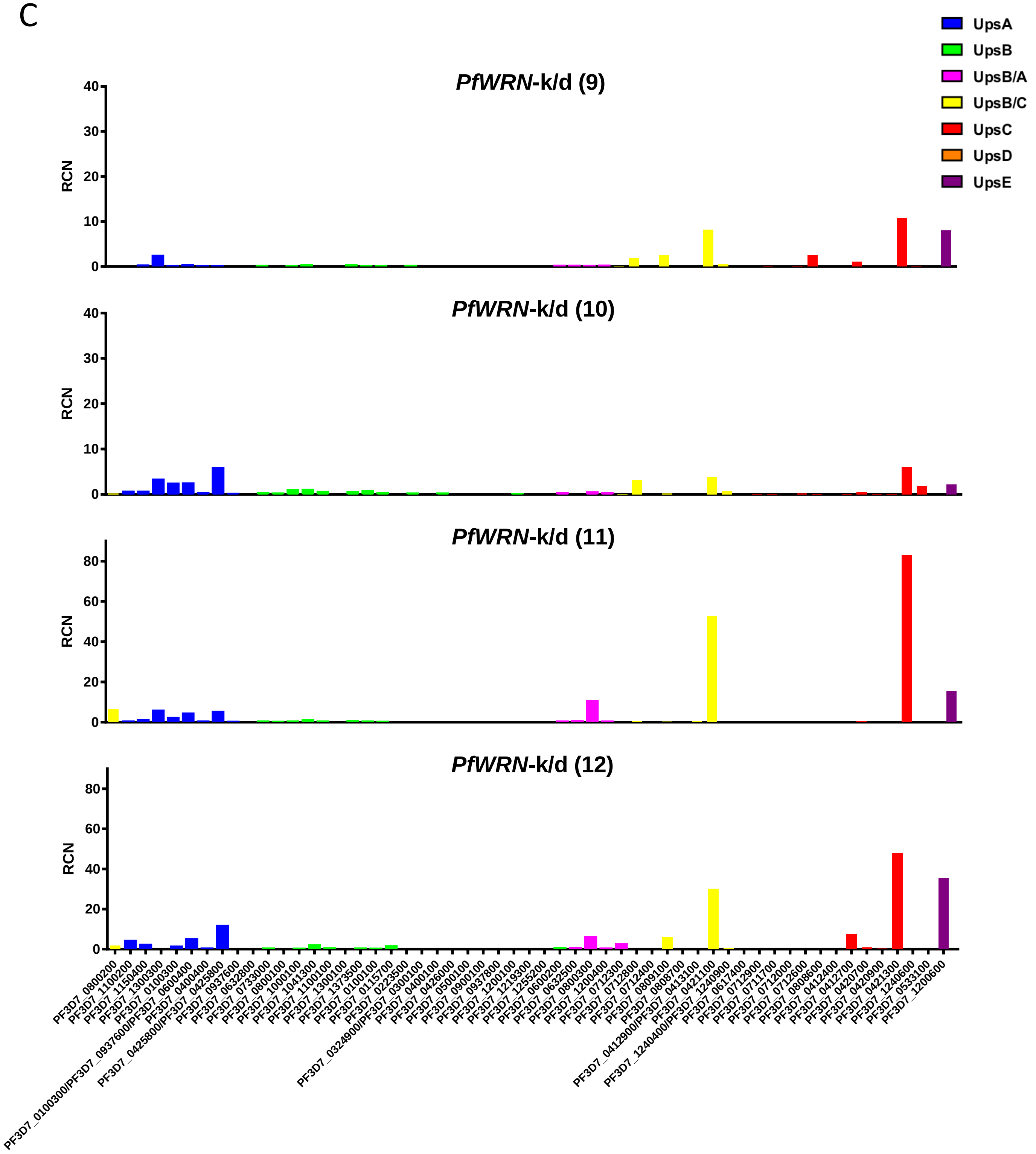

D

Parasite line	Sum of var variation	Percentage of variation in WT
3D7 wildtype	707.6	100%
ΔPfBLM	192.2	27%
PfWRN-k/d	521.8	74%
