# Supplementary material for: RecQ helicases in the malaria parasite Plasmodium falciparum affect genome stability, gene expression patterns and DNA replication dynamics
Source: PLoS Genet. 2018 Jul 2;14(7):e1007490. doi: 10.1371/journal.pgen.1007490 (PMC6044543; doi:10.1371/journal.pgen.1007490)
Supplement: S1 Table — For each set of deregulated genes in rings, trophozoites and schizonts of the RecQ helicase mutants, mean and median distance-to-nearest-PQS, tandem-repeat content and low-complexity-region content are shown. These values are plotted in Fig 4. (PDF) [file pgen.1007490.s021.pdf]

Table S1

Deregulated genes in RecQ helicase mutants are associated with sequences having the potential to form unusual DNA structures

Gene set			Putative Quadruplex Sequence (PQS)			Tandem Repeats (TRs)			Low-complexity Regions (LCRs)		
		Number	Mean distance from PQS (kb)	Median distance from PQS (kb)	Significant PQS association?	Mean TR content (%)	Median TR content (%)	Significant difference in TR content?	Mean LCR content (%)	Median LCR content (%)	Significant difference in LCR content?
All genes		5602	307.7	190.5		7.8	6.4		19.5	19.2	
∆BLM	Rings Down	159	252.5	130.9	Y (p = 0.03)	7.0	4.7	N (p = 0.24)	18.1	16.2	N (p = 0.14)
	Rings Up	146	263	138.0	N (p = 0.09)	10.1	8.7	Y (p = 0.001)	25.6	25.7	Y (p < 0.0001)
	Trophs Down	35	235.6	126.0	N (p = 0.18)	7.3	6.2	N (p = 0.74)	18.4	17.0	N (p = 0.60)
	Trophs Up	74	258	118.6	N (p = 0.18)	8.3	7.7	N (p = 0.56)	20.6	17.8	N (p = 0.41)
	Schiz Down	41	224.6	125.9	N (p = 0.10)	8.6	8.5	N (p = 0.51)	19.5	18.4	N (p = 0.99)
	Schiz Up	38	251.7	118.6	N (p = 0.28)	7.1	7.1	N (p = 0.63)	17.6	17.5	N (p = 0.32)
WRN-k/d	Rings Down	702	318.9	213.0	N (p = 0.38)	8.6	7.5	Y (p = 0.012)	22	21.8	Y (p < 0.0001)
	Rings Up	185	322.4	208.5	N (p = 0.54)	9.7	8.2	Y (p = 0.002)	24.7	25.1	Y (p < 0.0001)
	Trophs Down	7	331	130.2	N (p = 0.85)	6.2	8.6	N (p = 0.61)	16.8	16.2	N (p = 0.53)
	Trophs Up	21	100.7	22.1	Y (p = 0.003)	3.2	1.5	Y (p = 0.011)	15.6	17.2	N (p = 0.12)
	Schiz Down	8	394.2	286.8	N (p = 0.44)	8.9	8.7	N (p = 0.70)	16.4	16.2	N (p = 0.46)
	Schiz Up	15	54.8	11.0	Y (p = 0.002)	7.0	6.6	N (p = 0.73)	17.7	17.5	N (p = 0.54)

For each set of deregulated genes in rings, trophozoites and schizonts of the RecQ helicase mutants, mean and median distance-to-nearest-PQS, tandem-repeat content and low-complexity-region content are shown. These values are plotted in Figure 6.
