# Supplementary material for: RecQ helicases in the malaria parasite Plasmodium falciparum affect genome stability, gene expression patterns and DNA replication dynamics
Source: PLoS Genet. 2018 Jul 2;14(7):e1007490. doi: 10.1371/journal.pgen.1007490 (PMC6044543; doi:10.1371/journal.pgen.1007490)
Supplement: S2 Table — The parameters from two independent experiments of DNA molecular combing are shown. (PDF) [file pgen.1007490.s022.pdf]

Table S2

FIRST EXPERIMENT

	3D7 WT	Δ*PfBLM*	*PfWRN*-k/d	3D7 WT TMPyP2	Δ*PfBLM* TMPyP2	*PfWRN*-k/d TMPyP2	3D7 WT TMPyP4	Δ*PfBLM* TMPyP4	*PfWRN*-k/d TMPyP4
**Velocity (kb/min)**									
Number of values	204	255	152	302	347	230	289	295	212
Minimum	0,134	0,2762	0,1675	0,067	0,1675	0,1005	0,134	0,2345	0,2439
25% Percentile	0,7005	0,6067	0,5695	0,6365	0,5695	0,5695	0,5453	0,5025	0,4854
Median	0,9289	0,7821	0,7035	0,804	0,7106	0,7705	0,7067	0,6675	0,6775
75% Percentile	1,143	0,9767	0,9368	1,006	0,9593	1,055	0,907	0,8435	0,8729
Maximum	2,188	2,815	2,75	2,117	1,979	3,042	1,995	1,748	1,559
Mean	0,9281	0,8132	0,7531	0,8479	0,7773	0,8239	0,7643	0,6942	0,7055
**IOD (kb)**									
Number of values	139	319	137	241	218	171	158	212	154
Minimum	6,365	8,482	9,07	7,821	7,37	8,87	6,505	8,613	10,47
25% Percentile	33	28,24	26,41	28,66	24,3	30,18	25,81	21,53	21,97
Median	50,77	40,55	37,65	44,22	40,7	43,93	38,22	32,29	30,78
75% Percentile	79,4	60,27	55,94	64,41	61,18	60,44	59,14	56,17	51,39
Maximum	175,1	232,4	155,1	183,6	171,7	283,6	216,5	193,6	205,7
Mean	59,29	50,27	44,15	51,64	46,53	51,09	47,18	42,31	38,42
**Asymmetrical forks (long fork to short fork ratio)**									
Number of values	88	133	56	98	115	87	98	103	92
Minimum	1	1	1	1	1	1	1	1	1
25% Percentile	1,019	1,055	1,038	1,043	1,084	1,026	1,11	1,092	1,13
Median	1,063	1,242	1,163	1,212	1,259	1,264	1,384	1,257	1,299
75% Percentile	1,178	1,795	1,546	1,81	1,853	1,737	1,733	1,674	1,714
Maximum	6,109	4,541	3,193	5,4	7,04	4,99	13,5	11,79	7,994
Mean	1,254	1,536	1,414	1,544	1,656	1,633	1,745	1,566	1,639
**Number of forks**									
symmetric	55	44	21	35	34	32	19	27	19
asymmetric	33	89	35	63	81	55	79	76	73
unidirectional	6	33	18	35	41	15	32	29	11
total number	94	166	74	133	156	102	130	132	103

SECOND EXPERIMENT

	3D7 WT	Δ*PfBLM*	*PfWRN*-k/d	3D7 WT TMPyP2	Δ*PfBLM* TMPyP2	*PfWRN*-k/d TMPyP2	3D7 WT TMPyP4	Δ*PfBLM* TMPyP4	*PfWRN*-k/d TMPyP4
**Velocity (kb/min)**									
Number of values	372	406	274	383	326	247	333	344	277
Minimum	0,3089	0,3015	0,1675	0,134	0,1675	0,2345	0,2762	0,1005	0,268
25% Percentile	0,9104	0,8151	0,74	0,7491	0,7067	0,7161	0,7035	0,6733	0,6717
Median	1,186	1,058	0,9833	0,9715	0,9144	1,019	0,8736	0,9045	0,9045
75% Percentile	1,483	1,385	1,28	1,196	1,153	1,31	1,082	1,146	1,139
Maximum	4,413	3,454	2,881	2,246	2,724	2,848	2,203	2,714	2,02
Mean	1,225	1,143	1,03	0,9954	0,9661	1,045	0,9139	0,934	0,9116
**IOD (kb)**									
Number of values	226	193	124	250	213	189	163	214	167
Minimum	11,39	8,443	13,4	9,593	10,8	8,103	9,144	9,07	9,38
25% Percentile	39,17	35,88	34,05	31,13	35,22	31,67	33,83	30,59	30,49
Median	58,39	51,73	52,86	52,52	51,08	53,35	51,95	48,2	44,99
75% Percentile	91,87	82,6	74,29	84,43	76,77	79,76	74,7	73,92	70,03
Maximum	294,2	239,7	200,4	297,7	235,8	258,3	271,7	185,8	258,7
Mean	73,58	62,82	61,43	63,13	61,62	59,96	57,31	54,94	55,65
**Asymmetrical forks (long fork to short fork ratio)**									
Number of values	79	95	63	85	91	106	117	120	83
Minimum	1	1	1	1	1	1	1	1	1
25% Percentile	1,026	1,064	1,043	1,088	1,056	1,079	1,093	1,085	1,101
Median	1,081	1,335	1,241	1,206	1,22	1,293	1,269	1,253	1,316
75% Percentile	1,241	1,655	1,48	1,644	1,637	1,533	1,528	1,642	1,754
Maximum	2,923	3,362	2,761	3,981	4,455	4,717	4,552	5,388	4,286
Mean	1,228	1,495	1,356	1,412	1,419	1,503	1,476	1,435	1,499
**Number of forks**									
symmetric	43	28	22	23	28	31	30	37	20
asymmetric	36	67	41	62	63	75	87	83	63
unidirectional	6	15	9	8	10	7	17	12	13
total number	85	110	72	93	101	113	134	132	96
