# Supplementary material for: RecQ helicases in the malaria parasite Plasmodium falciparum affect genome stability, gene expression patterns and DNA replication dynamics
Source: PLoS Genet. 2018 Jul 2;14(7):e1007490. doi: 10.1371/journal.pgen.1007490 (PMC6044543; doi:10.1371/journal.pgen.1007490)
Supplement: S1 Method — (DOCX) [file pgen.1007490.s027.docx]

***Algorithm for analysing RNAseq data for proximity of differentially expressed genes to PQSs***

algorithm **g4_distances(F, G)**

input: F list of genes of interest as tuples (chromosome, start, end),

G list of G4 regions as tuples (chromosome, start, end)

output: D list of distances to nearest PQS for each gene in F

D <- empty list

for each gene f in F:

distance <- find_nearest_to_gene(f, G)

append distance to D

function **find_nearest_to_gene(f, G)**

input: f gene of interest,

G list of G4 regions,

output: X distance from f to nearest G

start_to_g4_distance <- find_nearest_to_end(f.chromosome, f.start, G)

end_to_g4_distance <- find_nearest_to_end(f.chromosome, f.end, G)

if start_to_g4_distance > end_to_g4_distance:

return end_to_g4_distance

else:

return start_to_g4_distance

function **find_nearest_to_end(c, p, G)**

input: c chromosome of target,

p position of target on chromosome,

G list of G4 regions as tuples (chromosome, start, end)

output: X distance from (c,p) to nearest in G

closest <- +Infinity

potentially_nearest <- filter G where G.chromosome = c

for each g in potentially_nearest:

if abs(p-g.start) < closest:

closest <- abs(p-g.start)

if abs(p-g.end) < closest:

closest <- abs(p-g.end)

return closest
